# Magnetically and optically active edges in phosphorene nanoribbons

**DOI:** 10.1038/s41586-024-08563-x

**Published:** 2025-03-12

**Authors:** Arjun Ashoka, Adam J. Clancy, Naitik A. Panjwani, Adam Cronin, Loren Picco, Eva S. Y. Aw, Nicholas J. M. Popiel, Alexander G. Eaton, Thomas G. Parton, Rebecca R. C. Shutt, Sascha Feldmann, Remington Carey, Thomas J. Macdonald, Cheng Liu, Marion E. Severijnen, Sandra Kleuskens, Loreta A. Muscarella, Felix R. Fischer, Hilton Barbosa de Aguiar, Richard H. Friend, Jan Behrends, Peter C. M. Christianen, Christopher A. Howard, Raj Pandya

**Affiliations:** 1 Cavendish Laboratory, University of Cambridge, Cambridge, UK; 2 Department of Chemistry, University College London, London, UK; 3 Berlin Joint EPR Laboratory, Fachbereich Physik, Freie Universität Berlin, Berlin, Germany; 4 Department of Chemistry, University of California, Berkeley, Berkeley, CA USA; 5 Interface Analysis Centre, School of Physics, H. H. Wills Physics Laboratory, University of Bristol, Bristol, UK; 6 Department of Physics and Astronomy, University College London, London, UK; 7 Yusuf Hamied Department of Chemistry, University of Cambridge, Cambridge, UK; 8 Institute of Chemical Sciences and Engineering, École Polytechnique Fédérale de Lausanne, Lausanne, Switzerland; 9 Department of Chemistry and Centre for Processable Electronics, Imperial College London, London, UK; 10 Department of Electronic and Electrical Engineering, University College London, London, UK; 11 High Field Magnet Laboratory (HFML—EMFL), Radboud University, Nijmegen, the Netherlands; 12 Center for Nanophotonics, AMOLF, Amsterdam, the Netherlands; 13 Department of Physics and Astronomy, Vrije Universiteit Amsterdam, Amsterdam, Netherlands; 14 Kavli Energy NanoSciences Institute at the University of California Berkeley and the Lawrence Berkeley National Laboratory, Berkeley, CA USA; 15 Materials Sciences Division, Lawrence Berkeley National Laboratory, Berkeley, CA USA; 16 Laboratoire Kastler Brossel, ENS-Université PSL, CNRS, Sorbonne Université, Collège de France, Paris, France; 17 Department of Chemistry, University of Warwick, Coventry, UK

**Keywords:** Magnetic properties and materials, Two-dimensional materials, Electronic properties and materials, Magnetic properties and materials, Two-dimensional materials

## Abstract

Nanoribbons, nanometre-wide strips of a two-dimensional material, are a unique system in condensed matter. They combine the exotic electronic structures of low-dimensional materials with an enhanced number of exposed edges, where phenomena including ultralong spin coherence times^1,2^, quantum confinement^3^ and topologically protected states^4,5^ can emerge. An exciting prospect for this material concept is the potential for both a tunable semiconducting electronic structure and magnetism along the nanoribbon edge, a key property for spin-based electronics such as (low-energy) non-volatile transistors^6^. Here we report the magnetic and semiconducting properties of phosphorene nanoribbons (PNRs). We demonstrate that at room temperature, films of PNRs show macroscopic magnetic properties arising from their edge, with internal fields of roughly 240 to 850 mT. In solution, a giant magnetic anisotropy enables the alignment of PNRs at sub-1-T fields. By leveraging this alignment effect, we discover that on photoexcitation, energy is rapidly funnelled to a state that is localized to the magnetic edge and coupled to a symmetry-forbidden edge phonon mode. Our results establish PNRs as a fascinating system for studying the interplay between magnetism and semiconducting ground states at room temperature and provide a stepping-stone towards using low-dimensional nanomaterials in quantum electronics.

## Abstract (web summary)

Phosphorene nanoribbons demonstrate extraordinary magnetic properties, ranging from large internal fields in films to macroscopic alignment in solution, which can be coupled to photoexcitations that localize to the magnetic edge of these ribbons.

## Main

Over the past 40 years, strategies to couple different magnetic phenomena and the electronic ground states of semiconductors have mostly focused on doping semiconductors with certain transition metals to form so-called dilute magnetic semiconductors (DMSs)^7,8^. A more recent approach has been to exploit the unique spin configurations that can be realized in two-dimensional (2D) materials: some of which have been also shown to host semiconducting magnetic phases^9,10^. Slicing 2D monolayers into thin strips, nanoribbons, opens the possibility for an even wider range of magnetic spin-arrangements across and along the ribbon edges. The most intensely studied nanoribbons have been those based on graphene^11,12^ (GNRs), in which topologically engineered bands^4,13,14^ and a range of spin ordered electronic states have been observed^1,5^. However, the laborious chemical synthesis^15,16^ needed to achieve the aforementioned phenomena, alongside the typically short (sub-100 nm) lengths of GNRs represents a challenge for applications and exploring the fundamental physics. Consequently, there is a need to explore nanoribbon systems that can be produced at scale and show room-temperature intrinsic spintronic and electronic properties. Among the mooted systems, phosphorene nanoribbons (PNRs), the black phosphorus analogue to GNRs, have uniquely been proposed^17–20^.

**Fig. 1:**
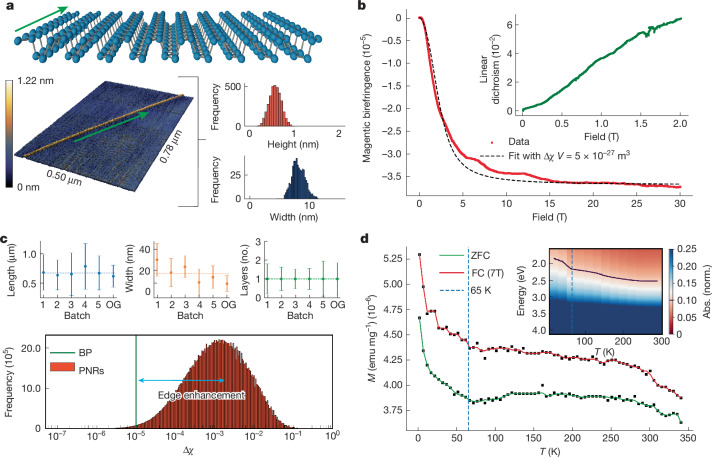
Shape, magnetic and optical anisotropy of PNRs. **a**, Top, crystal structure of the synthesized PNRs with the zigzag-aligned edges along their long axis (green arrow). Bottom, representative AFM image and histogram of height and width along a ribbon. An average width of 8 nm and average monolayer height of 0.6 nm is found along this ribbon (Methods and Supplementary Note 3). **b**, Alignment of PNRs in solution measured using magnetic field-induced linear birefringence up to 30 T shows saturation at 10 T. Combined with the magnetic linear dichroism (inset), these results indicate that PNRs align with their short axis along the field direction. We fit the data using a rigid-rod approximation for the PNRs^29^. Data in red, fit in black **c**, Bottom, histogram of the intrinsic anisotropy calculated using the distribution of all possible PNR volumes in the solution based on transmission electron microscopy and AFM data (Supplementary Notes 1 and 8). Top, the mean (and standard deviation) in length, width and layer number across five PNR batches measured in this work, and that from the original synthesis reported by Watts et al.^21^ (‘OG’). **d**, Field-cooled (FC) at 7 T and ZFC magnetization (*M*) as a function of temperature for a batch PNR samples prepared in a plastic straw measured in a projecting field of 50 mT (see Supplementary Fig. 22 for projecting field dependence). The FC and ZFC curves are split at all temperatures, with a discontinuity around 65 K, indicating a phase transition (*M* versus 1/*T* plots in Supplementary Fig. 38). The inset shows the PNR absorption (Abs.) spectrum between 300 and 10 K; here there is also a marked discontinuity near 65 K (the dark blue line marks a contour of absorption of 0.1). Norm., normalized.

PNRs can be readily fabricated, including by top-down approaches that produce micrometre-length, high-aspect-ratio ribbons with a long axis aligned exclusively in the zigzag crystallographic direction of the black phosphorus parent lattice (Fig. 1a)^21^—key characteristics for nanoribbon-based applications. The predicted presence of a visible band-gap^17^ and edge-ferromagnetism, with a high Curie temperature, makes PNRs intriguing^17,19,20^ for studying the intersection between functional electronic properties and magnetism in low dimensions^22^.

In this work we study width-uniform PNRs (sub-1-nm standard deviation along a ribbon) with zigzag-aligned edges^21^, at the ensemble level in both solution and on substrate. (Supplementary Notes 1 and 2). PNRs are produced in batch solutions with average ribbon widths of roughly 15 nm, average lengths of roughly 700 nm and predominantly as monolayers (greater than 70%; Supplementary Note 1). Figure 1a shows an atomic force microscopy (AFM) image of a typical individual monolayer ribbon (height of 0.58 ± 0.14 nm and width of 8.12 ± 1.08 nm) along with histograms of its width and height distributions measured along the ribbon using high-speed AFM (Supplementary Note 3).

## Magnetic properties of PNRs

To investigate the magnetic ground state of PNRs, it is important to first establish the degree of intrinsic magnetic anisotropy^23,24^ achieved through magnetic field-induced alignment experiments (linear dichroism and linear birefringence^25^). As shown in the magnetic linear birefringence data from a solution of PNRs in Fig. 1b, the orientational anisotropy of PNRs saturates near 10 T at room temperature, much lower than that of other known nanomaterials^26–28^. On fitting the data to the orientational magnetic energy^29^ we retrieve the anisotropy in the volume magnetic susceptibility (Δ*χ*) multiplied by the volume (*V*) of the material, Δ*χV* *=* 5 × 10^−27^ m^3^ (Supplementary Notes 6 and 7). We also observe a magnetic field-induced linear dichroism signal at 543 nm, with more light absorbed by the PNR solution when the light is polarized along the magnetic field direction (Fig. 1b, inset). PNRs are expected to have a higher absorption cross-section with light polarized perpendicular to the zigzag direction, as has been reported experimentally for phosphorene^24^ and obtained from GW-BSE (GW-Bethe–Salpeter Equation) calculations on PNRs^17,24^. We hence conclude that in a magnetic field, the ribbons orient in solution with their short axis along the field direction.

From the distribution of PNR volumes in solution (Supplementary Note 8 and Fig. 1c) and the calculated Δ*χV*, we extract an average anisotropy in the dimensionless magnetic susceptibility Δ*χ* ≈ 10^−3^ (Fig. 1c, bottom). This is two orders of magnitude larger than the values of Δ*χ* for layered phosphorene; that is, black phosphorous^23^ (Fig. 1c, green line). We infer that this 100-fold enhancement in Δ*χ* probably arises from the enhanced number of edges of the ribbons as this is the main intrinsic difference between the layered phosphorene and PNRs.

To understand the large Δ*χ* of PNRs it is critical to investigate the presence of unpaired spins. We hence perform superconducting quantum interference device (SQUID) magnetometry on an ensemble of dropcast PNRs (see Supplementary Note 9 for details on the sample morphology)^30,31^. The 7-T field-cooled (FC) and zero field-cooled (ZFC) magnetization measured in a 50-mT projecting field (Fig. 1d) shows non-Curie behaviour as we approach temperatures as high as 350 K in which the two curves approach each other but do not yet overlap. This is independently confirmed against background-subtracted, and subsequently fitted, raw SQUID traces (Supplementary Notes 10 and 11). The response also does not change when measured in lower projecting fields of 20 mT (Supplementary Fig. 22), indicating that the behaviour does not emerge due to an interaction with the measurement or projecting field of the SQUID. In the solution phase (*N*-methyl-2-pyrrolidone (NMP) and dimethylformamide (DMF) solvents), we also find that the temperature dependence of the field-cooled and ZFC magnetization is split up to the freezing point of the solvent (Supplementary Fig. 23). The invariance of the PNR SQUID signals in the frozen solution phase, and when deposited on both plastic and quartz substrates suggests that the magnetic phase we find is potentially robust against strain^32,33^ in these environments (Supplementary Note 10). Repeating the solution measurements on oxygenated PNRs, where the ribbon edge is expected to chemically degrade^21,34–36^, we find a purely diamagnetic response^37,38^ (see Supplementary Fig. 25 and Supplementary Notes 12 and 13 where we also rule out impurity contributions to the magnetic signals). Overall, this suggests that the magnetic response of PNRs arise from the ribbon edge with the persistence of the magnetic behaviour up to room temperature^18,20^ (Supplementary Notes 10 and 11).

Between 55 and 70 K in the *M*(*T*) data, a discontinuity is observed in Fig. 1d (seen more clearly when plotting *M* versus 1/*T* as shown in Supplementary Fig. 38), a signature of a magnetic phase transition. A similar discontinuity is also observed in the optical absorption at this temperature (Fig. 1d, inset) as well as in temperature dependent Raman and photoluminescence measurements (Supplementary Note 14). These independent experimental observations confirm that the optically active species (that is, the PNRs) are the source of our magnetic signal, and offer a first hint that the electrons involved in the magnetic phase transition could also be involved in the optical excitations.

## PNR edge spins

**Fig. 2:**
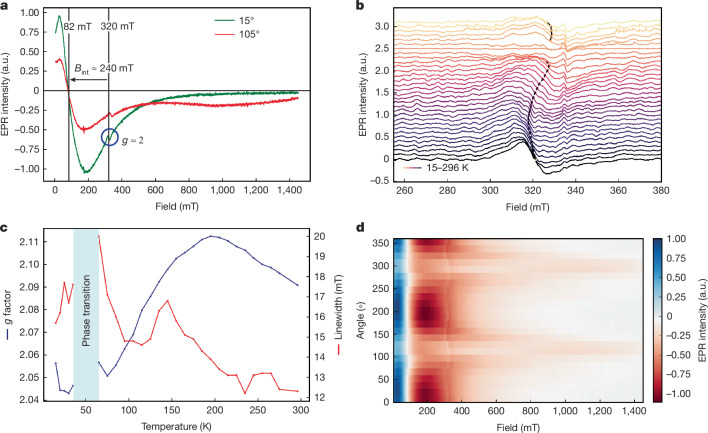
EPR and FMR local magnetic correlations. **a**, The cwEPR signal of PNRs in the solid state measured over a wide magnetic field range (at room temperature) shows both the unpaired electron peak at 320 mT sitting atop a much larger FMR  signal at 82 mT, suggesting an internal field of 240 mT (green curve; 15°, low field signal: internal field aligned to the magnetic field). The sample (a PNR film made in the inner wall of an EPR tube; Supplementary Notes 9 and 15) is highly inhomogeneous, hence rotation results in a shift in the resonance positions (red curve; 105°, high field signal: internal field anti-aligned to the magnetic field). **b**, Line cuts of the *g* ≈ 2 signal along with the zero-intensity crossing (dashed black line) as a function of temperature. **c**, Extracted *g* factor and linewidth as a function of temperature. We observe a divergence in the linewidth around 40 to 65 K characteristic of a phase transition. **d**, Orientational magnetic anisotropy in the EPR (FMR) signal of the ‘inner wall’ PNR sample across the full range of rotation angles of the tube at room temperature (see Supplementary Fig. 52 for the relationship between resonator and EPR tube axes). a.u., arbitrary units.

Next, we investigate the local spin correlations along the edge of PNRs by performing continuous wave electron paramagnetic resonance (cwEPR) measurements on a film formed on the inner wall of an EPR sample tube (see Supplementary Note 9 for the sample morphology). Consistent with the hypothesis of unpaired electrons at the ribbon edge we observe a broad roughly 12–19 mT wide, *g* *≈* 2 EPR signal at room temperature as shown in Fig. 2a,b. However, a wider magnetic field scan (0–1,400 mT) reveals far more intense and even broader ferromagnetic resonance (FMR) signals (Fig. 2a).

The EPR signal also shows a strong temperature dependence, where the linewidth and *g* factor values are non-monotonic, with a divergence of the linewidth and the disappearance of the signal on cooling to roughly 50 K, which reappears at lower temperatures (Fig. 2c; same temperature as the signature observed in SQUID, Raman measurements and so on; Supplementary Note 14). On rotating the sample tube in the EPR spectrometer, we discover that the EPR and FMR signals both show a strong orientational dependency around the sample tube long axis, as shown in Fig. 2d (see also Supplementary Note 15 for a diagram of the rotation axes and background signals). This behaviour arises from the inhomogeneity of the inner wall film in thickness, packing, PNR orientations and size (Supplementary Note 9).

To shed light on the internal fields within the EPR film samples, we fit the FMR signal with two purely Lorentzian and one Voigtian component (Supplementary Fig. 44). We find that some PNR domains give rise to net internal fields of roughly 225–265 mT and are aligned to the external magnetic field direction, thereby shifting the effective resonance position to lower magnetic fields at around 55–95 mT. We also find certain PNR domains where the effective resonance position is strongly orientation dependent, ranging from 850 to 240 mT. This orientation-dependent FMR behaviour is reminiscent of magnetism in ensembles of different sized Ni nanocubes^32^. Here changes in resonance position with changing orientation were ascribed to domains that are hard to (re-) magnetize, resulting in low field signals when the net internal field is (close to) aligned to the external magnetic field direction (hard axis), and high field signals when the net internal field is anti-aligned to the external magnetic field direction (easy axis). PNR domains showing an easy and hard axis could explain the FMR signals with weak and strong orientation dependencies, respectively. In this case, the net internal field of these PNR domains would be estimated to be roughly 600 mT considering the minimum and maximum resonance positions achieved through sample rotation (see Supplementary Notes 15 and 16 for further discussions and estimations of the PNR spin polarization length).

## Electronic dynamics

Having established room-temperature magnetism in PNRs, we turn to their optical (semiconducting) properties. PNRs have a featureless absorption spectrum, and under pressure (up to 300 MPa) the absorption-edge blue-shifts (six times greater than that of phosphorene for the same applied pressure^39^; Supplementary Note 19), with applied electric fields showing a substructure within the absorption spectrum (Supplementary Note 22). Emission measurements on individual PNRs (Supplementary Note 18) indicate a band-gap that is inversely proportional to the PNR width (Supplementary Note 20).

**Fig. 3:**
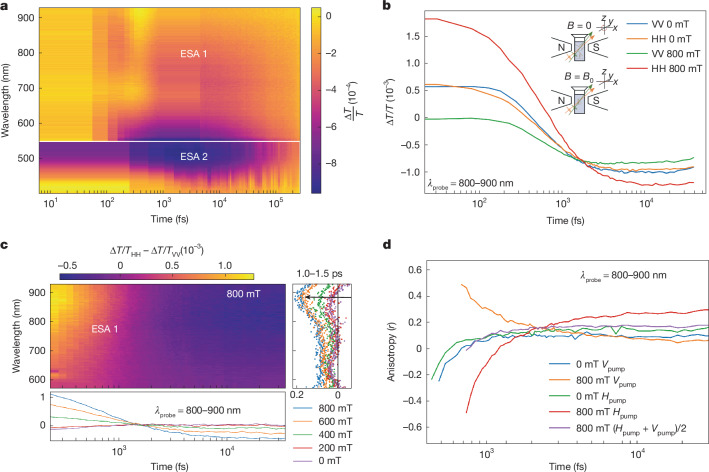
Photoexcited dipole relaxation dynamics. **a**, Broadband transient absorption spectrum of PNRs. On photoexcitation the PNRs show two broad ESA bands that decay on the order of nanoseconds. There seems to be a broad photobleaching signature at 700 nm that overlaps with the ESA bands. **b**, Polarization- and magnetic field-resolved decay of ESA 1 kinetics. On application of an 800-mT field, the ribbons align in the magnetic field, splitting the degeneracy of the horizontal pump–horizontal probe (HH) and vertical pump–vertical probe (VV) polarized experiments. Further, we find a significant reduction in the signal magnitude when photoexciting *V* polarized (along the PNR long axis). Inset shows the orientation of PNRs with respect to light polarization and magnetic field. **c**, Magnetic field-induced anisotropy obtained by subtracting the spectral response of the unaligned ribbons. This unveils a spectral signature centred at 850 nm (corresponding to ESA 1), the magnitude of which can be strongly modulated with a sub-1-T field (right). **d**, Decay of polarization anisotropy (*r*) as a function of magnetic field. In an unaligned solution under no magnetic field, we find that the anisotropy is initially −0.2 and decays to close to 0 within 1 ps (green and blue), indicating the photoexcited state is perpendicular to the probed state after 1 ps (the differences in the *V* and *H* polarized experiments at 0 mT demonstrate the setup’s resolution). On application of the 800-mT field, however, photoexciting *V* polarized (PNR long-axis dipole, orange curve), we find that the anisotropy retains its sign (indicating the photoexcited state is the same orientation as the probed one) whereas when photoexciting *H* polarized (PNR short-axis dipole, red curve), the anisotropy flips sign, indicating a dipole flip, as in the unpolarized case, with the average converging to the unpolarized system, as expected.

Focusing on the electronic dynamics using optical pump–probe spectroscopy, we see the emergence of two long-lived (nanosecond) excited state absorption (ESA) bands at 800 nm (ESA 1) and 500 nm (ESA 2) along with an overlapping, short-lived (picosecond) broad photobleaching band at 700 nm (Fig. 3a). In Fig. 3b, we show the results of performing a polarization-resolved pump–probe experiment on the PNRs in solution, with an 800 mT applied external magnetic field in the Faraday geometry (where ribbons align with their short axis along the magnetic field direction; Fig. 1b). We find that the typical copolarized horizontal pump–horizontal probe and vertical pump–vertical probe signal’s degeneracy is lifted and a laboratory axis is introduced into the experiment on application of an 800-mT field. The magnetic field is polarized along the *H* direction in the laboratory axis that, as expected, results in the higher transient absorption signal (which scales with field; Supplementary Note 21) due to the increased net linear dichroism along the horizontal pump–horizontal probe direction (Fig. 1b).

We leverage this magnetic alignment effect to isolate the spectral features of the magnetically aligned ribbons (as opposed to optical signatures of a non-magnetic subpopulation) by subtracting the horizontal pump–horizontal probe and vertical pump–vertical probe polarized transient absorption maps at different field strengths. As seen in Fig. 3c, we find that the magnitude and kinetics of the photoinduced absorption band (ESA 1) is most strongly perturbed by the external field. We can therefore confidently assign ESA 1 to that of the magnetically active ribbons.

ESA 1 (Fig. 3a) forms quickly (sub-1 ps) on the disappearance of the two positive photobleaching-like spectral features. To understand this and track the evolution of the photoexcited dipole’s relaxation dynamics in the magnetically active ribbons, we monitor the transient absorption anisotropy in the ESA 1 band (800–900 nm) defined as

$$r=\frac{{\frac{\Delta T}{T}}_{\parallel }-{\frac{\Delta T}{T}}_{\perp }}{{\frac{\Delta T}{T}}_{\parallel }+2{\frac{\Delta T}{T}}_{\perp }},$$

where ${\frac{\Delta T}{T}}_{\parallel }$ and ${\frac{\Delta T}{T}}_{\perp }$ are the transient absorption signal with the probe polarization parallel and perpendicular to the pump polarization^33^. We find that, in the absence of an external field, at 200 fs the anisotropy is −0.2 (blue-green traces), signifying that we are probably probing the dipole polarized orthogonally to the main photoexcited transition. After 1 ps, the anisotropy relaxes to a value of about 0.05, signifying that there is energetic transfer between the dipoles as the timescale is faster than the nanosecond-orientational relaxation dynamics typically associated with molecular anisotropy.

On application of the 800-mT field, because of the macroscopic alignment of the ribbons, we can increase our dipole selectively and pump the short-axis polarized (*H*) and long-axis polarized (*V*) dipoles. As the ribbons are now aligned, the sign of the anisotropy is the key feature of interest as the magnitude will not be consistent with the isotropic molecular distribution assumption standard to such measurements^40^. We find that when pumping the long-axis polarized (*V*) dipole (Fig. 3d, orange), the anisotropy does not change sign, indicating that the energy remains in this state, whereas when we pump the short-axis polarized (*H*) dipole, the sign flips (red), just like in the case of the unpolarized ribbons (blue). Further, the fact that we can construct the unaligned PNR anisotropy as an average of pumping the 800 mT aligned long-axis polarized (*V*) and short-axis polarized (*H*) dipoles (Fig. 3d, purple) suggests that these are the main two dipoles involved in the optical response of the system. Taking this observation with GW-BSE calculations of zigzag PNRs, which report that the lowest excited state is a dark exciton polarized along the ribbons long axis^17^, we suggest the sub-1-ps decay is probably into a (long-lived) long-axis polarized dark state. We remark our that transient absorption microscopy measurements on single PNRs show a distribution (roughly 0.75-ps standard deviation) in the ESA 1 lifetimes indicating the timescales above are an ensemble average (Supplementary Note 18).

## Magneto-optical coupling

**Fig. 4:**
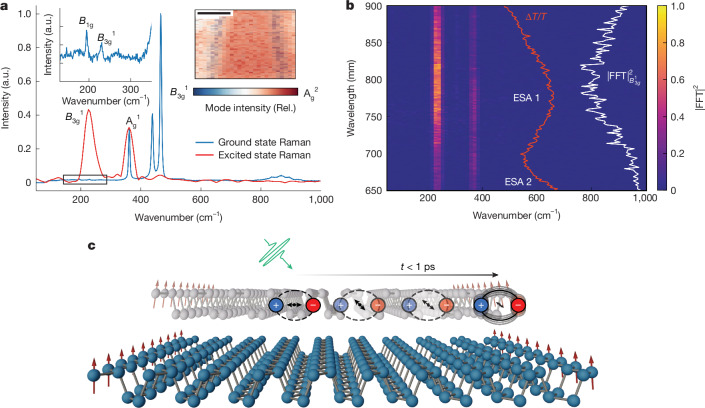
Coupling of the photoexcitation to the magnetic edges. **a**, The excited state (impulsive, time domain) Raman spectrum of PNRs (red) shows a strong coupling to the *B*_3g_^1^ mode compared to the ground state spectrum (blue), indicating that the photoexcitation is delocalized along the edges of the ribbon. Inset, on the left, zoom into the low-frequency region of the ground state Raman spectrum. Right, Raman imaging of a black phosphorous flake shows that, as previously reported^41^, the *B*_3g_^1^ mode is localized on the edge. We note that the (broad) linewidth in the IVS experiments is, as a result of the instrument resolution and Fourier transforming of the time-domain spectrum, not inhomogeneous or lifetime broadening. **b**, Wavelength-resolved, impulsive, time-domain Raman spectrum of the photoexcited PNRs reveals that the *B*_3g_^1^ mode is most strongly coupled (white curve) to ESA 1 (red curve), which was also most strongly modulated by the external magnetic field. **c**, Schematic of the photoexcited dipole dynamics, where the excited state is initially polarized along the PNR short axis and, on sub-1-ps timescales, relaxes to a dipole polarized along the long axis with significant density along the ribbon edge. Here the excitation is coupled to a symmetry-forbidden edge phonon mode, and it is at this edge where the magnetism is probably present. FFT, fast Fourier transform; Rel., relative. Scale bar, 500 nm.

To understand how the photoexcitation in PNRs relates to the edge, key for any magneto-optical coupling, we exploit the presence of special localized phonon modes on the PNR edges^41^. The spontaneous Raman spectrum of the PNRs (Fig. 4a, blue curve) is dominated by *A*^1^_g_, *B*^2^_g_ and *A*^2^_g_ symmetry modes, with extra bulk-symmetry-forbidden *B*^1^_g_ and *B*_3g_^1^ modes between 190 and 250 cm^−1^. All of these modes are also observed in the Raman spectrum of black phosphorous^42^ with the *B*^1^_g_ and *B*_3g_^1^ modes specifically being along the zigzag edge as previously reported (Raman image in Fig. 4a, inset)^41^. To study the mode coupling to the photoexcited state, we perform impulsive vibrational spectroscopy (IVS)^43^. We find that when resonantly photoexciting the PNRs, the photoexcited state is strongly coupled to the *B*_3g_^1^ edge phonon mode (Fig. 4a, red curve, also Supplementary Note 23). Furthermore, as seen in Fig. 4b, we find that this edge phonon mode is most strongly coupled to ESA 1, the transition characteristic of the magnetically active ribbons. The selective coupling of the excited state to the edge mode as opposed to the bulk phonon modes suggests that the electron density of the photoexcitation is along the edges of the PNRs. In the solution phase, we observe that the excited state IVS of the *B*_3g_^1^ out-of-plane edge mode is present when studying the unaligned (0 mT) ribbon solution only in the perpendicular pump–probe configuration, that is, horizontal pump–vertical probe or vertical pump–horizontal probe (Supplementary Note 23) and not the parallel configurations. In other words, one needs to probe a dipole perpendicular to the main photoexcitation (that we ascribe to the transition polarized along the PNRs long axis) to observe the excited state moving along the *B*_3g_^1^ vibrational coordinates at the edge of the PNRs, consistent with our proposed photoexcitation relaxation mechanism (Fig. 4c).

## Conclusion

In summary, we have evidenced by means of SQUID magnetometry and cwEPR, macroscopic magnetism at room temperature in films of PNRs, arising from the ribbon edges. In solution a giant magnetic anisotropy means PNRs can be readily aligned with their short axis along the field direction. As shown in Fig. 4c, on photoexcitation, energy rapidly migrates to a state with density that is localized to the PNR edge where the magnetism probably exists. Our findings open up extensive opportunities for further understanding the magnetic properties of PNRs (spin coherence times, influence of ribbon width or strain, role of thickness and so on), building of proposed single nanoribbon devices^44–46^ and for more disparate applications (nonlinear optical elements^47,48^ or nanorobotics^28,49^). The optically active nature of PNR edges provides an especially promising route for nanoscale magnetic-semiconducting interfacing, essential for low-energy computing, and refining theories of magnetism in low dimensions.

## Methods

### PNR synthesis

PNRs were synthesized as reported in ref. ^21^. In short, in an argon glovebox (less than 0.1 ppm O_2_, less than 0.1 ppm H_2_O) black phosphorus (2D Semiconductors Ltd or Smart Elements) was ground using a pestle and mortar into roughly 1-mm flakes and 124 mg (in a typical synthesis) was transferred to a glass tube fitted with a metal Swagelok valve, alongside 3.5 mg freshly cut lithium metal (Sigma Aldrich, 99% rod). The tube was evacuated (roughly 10^−7^ mbar) and cooled to −50 °C and ammonia gas (Sigma Aldrich, 99.95%, precleaned by condensation over excess lithium metal) was condensed to submerge the lithium and phosphorus. The solution immediately turned dark blue from the formation of lithium-ammonia solution, and slowly turned orange over 16 h. The ammonia was then evaporated and the LiP_8_ salt was dried under vacuum (roughly 10^−7^ mbar) at room temperature. The LiP_8_ (10 mg) was then placed in 10 ml of either NMP (Sigma Aldrich, 99.5% anhydrous) or DMF (Sigma Aldrich, 99.9% anhydrous) that had been dried with 4-Å molecular sieves for 1 week. The mixtures were bath sonicated for 30 min, before centrifuging (100*g*, 10 min) and decanting in a glovebox to give solutions of PNRs.

### Single PNR AFM

PNR samples were prepared by means of dropcasting from solution onto freshly cleaved graphite (HOPG) substrates as discussed in ref. ^21^. High-resolution topography maps were then collected using contact-mode high-speed AFM (HS-AFM; Bristol Nano Dynamics Ltd) with silicon nitride microcantilevers (MSNL-10, Bruker) with nominal tip radii of 2 nm. To ensure that the images obtained featured tip-limited lateral resolution scan sizes of 0.8 × 0.5 µm were used, corresponding to pixel sizes of roughly 1 × 1 nm.

HS-AFM images containing ribbons selected for analysis were input into custom LabVIEW image analysis software (National Instruments) that isolated the ribbon from the background in an automated fashion before generating histograms of ribbon height and width. In brief, the algorithm consisted of: a local thresholding step to identify the ribbon; a mask step to separate pixels corresponding to the background from the ribbon; an erosion step to remove the edge pixels where the AFM tip traverses the slope between the background and ribbon; calculation of height histograms for both the background and ribbon pixels and finally Gaussian fits to the histograms to recover the mean height of each and enable calculation of the height of the ribbon with respect to the background.

The width of the ribbon was analysed in parallel using the same algorithm. After the masking step the central axis of the isolated ribbon was determined by fitting cubic splines to the two edges of the ribbon and calculating the mean of these two edges. Then, the width of the ribbon was determined by calculating the distance to the central axis from each edge. Measurements of the width of the ribbon along its entire length were then input into a histogram and fit with a Gaussian distribution to identify the mean width of the PNR.

### Magnetic linear dichroism and birefringence

The degree of magnetic alignment is measured optically through the magnetic field-induced linear dichroism and birefringence by using green and red Helium Neon lasers (wavelengths 543 and 632.5 nm, respectively). The solution samples were contained in an optical cuvette (thickness 5 or 10 mm) positioned within a temperature-controlled environment at 20.0 ± 0.1 °C in a 33-T Florida–Bitter electromagnet or a Varian V-3900 2-T magnet. The linear dichroism and linear birefringence signals were measured using standard polarization modulation techniques using a photo-elastic modulator^50^.

### SQUID magnetometry

Magnetization measurements were obtained in a Quantum Design Magnetic Properties Measurement System (MPMS 3) using a SQUID magnetometer. Measurements were performed down to 1.8 K and up to 350 K, in various applied magnetic field strengths up to 7 T. Data for two magnetization–temperature curves of PNRs are shown in the inset of Fig. 1d. Each magnetization–temperature curve was measured starting at 1.8 K and increasing the temperature in a constant probing field of 50 mT. Before beginning any new magnetic field or temperature scan the system was taken to 300 K and the magnet was reset to remove any stray flux from the SQUID. Samples were secured on an MPMS quartz sample holder using GE varnish, and care was taken to ensure that they were not touched by any magnetic material throughout the mounting and loading procedure. For each measurement, several d.c. magnetization measurements were averaged, providing a reliable measure of the bulk magnetization of the PNR samples.

For measuring the PNRs in the DMF and NMP solutions (Supplementary Fig. 23), 10 μl were pipetted into a plastic (polypropylene) straw or capsule (polypropylene). The capsule was fixed to a quartz rod using GE varnish, and the presence of PNRs increased the magnetization by two orders of magnitude when compared to the GE and straw or capsule by itself. The d.c. moment was averaged over several scans, and between each experimental run the MPMS was brought to 300 K and the magnet was reset to remove any remaining flux in the SQUID. Oxygenation of the PNR was performed by bubbling air through PNRs in the plastic capsule for roughly 20 min while ensuring the sample it did not come into contact with any metallic substances. See also Supplementary Notes 10, 11 and 24 for further details.

### EPR

For the cwEPR experiments, 100 µl of PNR in DMF solution was placed into a 3.9-mm-outer-diameter (2.9-mm-inner-diameter) quartz EPR tube inside a nitrogen glovebox. The sample tube was attached to a custom adaptor and transferred to a pumping station outside the glovebox. The custom adaptor keeps the sample in the inert glovebox environment. The solution was evaporated under vacuum (using a pumping station), resulting in a film on the inner walls of the EPR tube. The procedure was repeated four times to achieve a thicker film and in total 400 µl was evaporated. The inner wall sample was then left to pump to a pressure of 6 × 10^−4^ mbar and flame sealed.

#### Narrow magnetic field scan rotation and temperature series (setup 1)

The cwEPR spectra were recorded at X-band (roughly 9.4 GHz) using a laboratory-built EPR spectrometer (all narrow scan cwEPR spectra were rescaled to 9.4 GHz). The setup for the rotation and temperature series consisted of a Bruker ER 041 MR microwave bridge together with an ER 048R microwave controller and an AEG electromagnet together with a Bruker BH 15 Hall effect field controller. The magnetic field was also monitored with a Bruker ER 035M NMR Gaussmeter. The resonator used was a Bruker ER 4122-SHQE resonator. The static magnetic field was modulated at 100 kHz and lock-in detection was carried out using a Stanford Research SR810 lock-in amplifier in combination with a Wangine WPA-120 audio amplifier. An ESR 900 helium flow cryostat together with a ITC503 temperature controller (Oxford Instruments) was used for low-temperature measurements. The spectra were acquired at a frequency of roughly 9.4 GHz with a microwave power of 7.96 mW and 1-mT modulation amplitude. The magnetic field was calibrated using a standard N@C60 sample with a known *g* factor.

The narrow magnetic field scans presented in the main text (Fig. 2c) have had the FMR signal (slope) removed from them using an appropriate polynomial fit. See Supplementary Note 15 for further details.

#### Wide magnetic field scan rotation study (setup 2)

The cwEPR spectra were recorded at the X-band (roughly 9.55 GHz) using a laboratory-built EPR spectrometer (all wide-scan cwEPR spectra were rescaled to 9.55 GHz). The setup for the rotation study consisted of a Bruker ER 046 XK-T microwave bridge together with an ER 048R microwave controller and a Varian electromagnet together with a Bruker ER 032M Hall effect field controller. The resonator used was a Bruker MD5 dielectric ring resonator. The static magnetic field was modulated at 99 kHz and lock-in detection was carried out using a Stanford Research SR830 lock-in amplifier in combination with a Wavetek 50 MHz function generator model 80. The wide magnetic field measurement was carried out at 296 K. The spectra were acquired at a frequency of roughly 9.55 GHz with a microwave power of 7.96 μW and 0.4-mT modulation amplitude.

#### Wide magnetic field scan without low-temperature insert rotation series (setup 3)

The wide magnetic field scan without a low-temperature insert (rotation series) was carried out on a Bruker ElexSys E580 spectrometer, with a Bruker ER 4122-SHQE resonator. The SHQE cavity is the same type of cavity as used in the narrow field range setup ‘setup 1’. However, this time we used the cavity without the low-temperature Dewar insert, as this insert can have background signals. The reason for using the Bruker ElexSys E580 is that it allowed us to do the full magnetic field sweep using the SHQE resonator. The spectra were acquired at a frequency of roughly 9.85 GHz with a microwave power of 8.05 μW and 0.4-mT modulation amplitude.

### Scanning tunnelling microscopy

All scanning tunnelling microscopy (STM) experiments were performed on a commercial Omicron LT-STM at 4.2 K using PtIr STM tips. Samples were prepared by aerosolizing PNRs dispersed in NMP onto a HOPG substrate to minimize aggregation on the surface^51^. The deposition parameters and the concentration and/or density of PNRs on the surface were optimized using ambient AFM imaging. For ultra-high vacuum-STM experiments PNRs were aerosolized onto a freshly cleaved HOPG substrate using an Iwata Custom Micron CM-C airbrush. The coated substrate was immediately transferred into the vacuum chamber and annealed for more than 1 h at 150 °C before transferring the substrate to the STM sample stage (extended annealing or heating greater than 150 °C led to partial degradation of PNRs). Supplementary Note 5 contains further details on the STM imaging.

### Transient absorption spectroscopy

The transient absorption measurements from 550 to 930 nm were performed using a home-built setup around a Yb-doped potassium gadolinium tungstate (Yb:KGW) amplifier laser (1,030 nm, 38 kHz, 15 W, Pharos, LightConversion). The probe pulse was a chirped seeded white light continuum created using a 4-mm yttrium aluminium garnet (YAG) crystal that spanned from 500 to 950 nm. For the source of the pump pulse (roughly 200 fs) a commercial optical parametric amplifier OPHEUS ONE (LightConversion) was used.

The transient absorption measurements from 400 to 550 nm were performed using a home-built setup around a Ti-sapphire (800 nm, 1 kHz, Spectra-Physics, Solstice Ace). The probe pulse was a chirped seeded white light continuum created using a 4-mm CaF_2_ crystal that spanned from 400 to 600 nm. For the source of the pump pulse (roughly 100 fs), the fundamental of the laser was doubled in a β-barium borate crystal.

The sub-1-T magnetic field was generated using an electromagnet from GMW Model 3470 with 1-cm distance between cylindrical poles and the field strength calibrated with a Gaussmeter.

### Impulsive Raman spectroscopy

Femtosecond time-domain Raman spectroscopy measurements were performed using a home-built setup around a Yb:KGW amplifier laser (1,030 nm, 38 kHz, 15 W, Pharos, LightConversion). The probe pulse was a chirped seeded white light continuum created using a 4-mm YAG crystal that spanned from 500 to 950 nm. The pump pulse for the resonant experiment was created using a non-collinear optical parametric amplifier where the 1,030 nm seeded a white light continuum stage in sapphire that was subsequently amplified with the third harmonic of the 1,030-nm laser in a β-barium borate crystal to create a broad pulse centred at 550 nm. The pump pulse for the off-resonant experiment was created using a non-collinear optical parametric amplifier where the 1,030 nm seeded a white light continuum stage in a YAG crystal that was subsequently amplified with the third harmonic of the 1,030-nm laser in a β-barium borate crystal to create a broad pulse centred at 750 nm. Both pulses were compressed using a chirped mirror and wedge prism (Layerterc) combination to a temporal duration of under 15 fs. Compression was determined by second-harmonic generation frequency-resolved optical gating (upper limit) and further confirmed by reference measurements on acetonitrile where the 2,200 cm^−1^ mode could be resolved. The probe white light was delayed using a computer-controlled piezoelectric translation stage (Physik Instrumente), and a sequence of probe pulses with and without pump was generated using a chopper wheel (Thorlabs) on the pump beam. The average fluence of the pump 10 μJ cm^−^^2^.

### Transient absorption microscopy

Pulses were delivered by a Yb:KGW amplifier (Pharos, LightConversion, 1,030 nm, 5 W, 200 kHz) that seeded two broadband white light stages. The probe white light was generated in a 3-mm YAG crystal and adjusted to cover the wavelength range from 650 to 950 nm by a fused-silica prism-based spectral filter. By contrast, the pump white light was generated in a 3-mm sapphire crystal to extend the white light in the high frequency to 500 nm, with pulse short-pass filtered at 650 nm (Thorlabs, FESH650). The pump pulses were focused onto the sample using a single-lens oil-immersion objective (×100, numerical aperture (NA) 1.1) to a diffraction-limited spot of roughly 270 nm (full-width at half-maximum, full bandwidth). By contrast, the counter-propagating probe pulses were loosely focused onto the sample by a concave mirror (full-width at half-maximum, roughly 15 μm). A set of third-order corrected chirped mirrors (pump white light, Layertec; probe white light, Venteon) in combination with a pair of fused-silica wedge prisms (Layertec) compressed the pulses to sub-15 fs at the sample. The transmitted probe light was collected by the same objective used to focus the pump pulses. The probe was then relayed to a spectrometer consisting of a slit in the intermediate image plane and a F2 prism to disperse the light perpendicular to the slit. This allowed us to access spectrally dispersed transient absorption microscopy images of the PNRs over a selected region. The differential nature of the imaging was achieved by modulating the pump beam at 45 Hz by a mechanical chopper. All recording was performed with an EMCCD camera (Qimaging Rolera Thunder, Photonmetrics). The axial focus position was maintained by an extra auto-focus line based on total internal reflection of a 405-nm continuous wave laser beam.

### Ensemble photoluminescence measurements

#### Steady-state photoluminescence

Photoluminescence experiments were performed using a home-made confocal-like setup using a large working-distance microscope objective (NA roughly 0.55) to focus light on the nanoribbons and collect the emission in a reflection configuration. In the studies, PNRs are dispersed (dropcasting method) on glass slides (thickness roughly 100 μm) and glued (with silver lacquer) on the cold finger of a cryostat designed for thermal expansion compensation (from Oxford Instruments). The photoluminescence signal was analysed by using a 75-cm focal length spectrometer (Acton sp2750i, Princeton Instruments) itself coupled to a nitrogen cooled CCD (Spec10, Princeton Instruments), a combination that leads to a roughly 100 μeV energy resolution, well beyond the resolution required to address the broad PNRs photoluminescence components. To minimize the scattered light, the excitation was tuned to 416 nm (second-harmonic generation of a Ti:sapphire laser, pulse width of roughly 2 ps, 80-MHz repetition rate) and a dichroic filter (Semrock FF01-430/LP-25, 50% cut-off at 437 nm) was placed in the detection path. The photoluminescence polarization was analysed using a classical scheme: a motorized half-waveplate, positioned upstream along the detection beam path, allowing us to rotate the polarization of the PNR emission that was further analysed using a polarizer (Glan-Taylor, calcite) placed in front of the spectrometer slit (with its direction set parallel to the grating grooves to enhance the CCD response).

#### Time-resolved photoluminescence

The spectro-temporal photoluminescence maps were measured using a streak-camera synchronized with the high repetition rate Ti:sapphire laser (C5680 model from Hamamatsu incorporating a M5675 synchroscan unit). All measurements were carried out in the confocal configuration described above, the camera being directly coupled to the Acton spectrometer, using a deflection mirror and imaging the nanoribbons’ spectra on the entrance slit of the camera. The response function of the camera was measured as the response to laser excitation with an instrument response function curve that demonstrates a time resolution of roughly 22 ps across the detection range to the setup. The same cryostat and mounting as for steady-state photoluminescence measurements were used for the time-resolved measurements.

### Single PNR photoluminescence imaging and spectroscopy

Spinning disk confocal photoluminescence measurements were performed with a commercial Nikon X-Light V2 microscope, with a ×100 1.52 NA oil-immersion objective. Excitation in all cases was at 390 nm with nominal laser powers (at the laser output) between 20 and 50 mW. Imaging was performed with a 40 μm confocal pinhole and iXon 897 EMCCD Camera (Andor). The lateral resolution in such measurements was around 120 nm. For the measurement of photoluminescence spectra from individual PNRs, the microscope emission outcoupling-arm was modified to pass the photoluminescence to a spectrograph (Kymeria 193i; 600 lines per millimetre grating; 600-nm blaze) with the emission once again measured by an iXon 897 EMCCD. Typical accumulation times of 1–2 min were used to collect spectra from the PNRs with a collection area on the sample of 0.1 μm.

### Absorption spectroscopy

#### Temperature dependent PNR absorption

An Agilent Cary 6000i ultraviolet–visible light with near infrared spectrophotometer with blank substrate correction was used. Here 400 μl of PNR solution was dropcast onto fused-silica substrates and placed in a continuous-flow cryostat (Oxford Instruments Optistat CF-V) under a continuous inert atmosphere. Samples were cooled to 6 K with the temperature dependent absorption taken on heating.

#### Individual PNR absorption spectroscopy

Absorption spectroscopy of individual (large, greater than 300 nm wide) PNRs was performed on a customized Zeiss Axio microscope with illumination provided by a halogen lamp (Zeiss HAL100). Transmitted light was collected using a ×50/0.4 objective (Nikon, T Plan SLWD) and spatially filtered using a 100-μm-diameter optical fibre (Avantes FC-UV100-2-SR) mounted in confocal configuration and connected to a spectrometer (Avantes AvaSpec-HS2048). PNR samples were prepared by means of dropcasting from solution onto cleaned (Acetone/IPA) 0.17-mm-thick glass slides.

### Pressure dependent absorption

To study the pressure dependence of the PNRs, transmittance spectra were measured with a LAMBDA 750 ultraviolet–visible light with near infrared spectrophotometer (Perkin Elmer). The PNR solutions were dried in an inert atmosphere and then placed inside a high-pressure cell (ISS Inc.) filled with an inert liquid, Fluorinert FC-72 (3 M). Hydrostatic pressure was generated through a pressurizing liquid using a manual pump. Before using, the liquid was degassed in a Schlenk line to remove oxygen that caused, from 300 MPa onwards, scattering of a fraction of light and therefore a reduction of the transmitted signal from the sample. The pressure was applied from ambient pressure to 300 MPa in steps of 50 MPa. Before the measurement, we waited 7 min for equilibration of the material under pressure. We estimated an error of the pressure reading to be 20 MPa.

### Raman measurements

#### Temperature-dependent Raman spectroscopy

Raman spectra were measured as a function of temperature from 4 to 300 K. Raman measurements were conducted by backscattering (T64000, Horiba) a continuous wave diode line (532 nm, 1 mW). Spectra were collected at more than 200 cm^−1^, where the CCD detector (Horiba Synapse Open-Electrode) had a monotonically increasing quantum efficiency of 0.43–0.50. Acquisitions used a ×100 optical objective and used minimal laser intensity to avoid sample degradation.

#### Raman imaging

For Raman imaging a standard layout of an epi-detected Raman microscope was used. A pump laser beam (wavelength 532 nm, Coherent Mira) was spectrally cleaned up by a bandpass filter (FLH05532-4, Thorlabs), and its beam width was expanded to 7.2 mm before entering a home-built inverted microscope. Further waveplates (half-waveplate and quarter-waveplate for 532 nm, Foctek Photonics) precompensated the ellipticity introduced by the dichroic filter (F38-532_T1, AHF) and also generated circularly polarized light. We used high NA oil-immersion objectives (Nikon ×60/1.4 NA oil) to ensure high-resolution imaging and increase collection efficiency. The pump power before the objective was 30 mW, a power level that ensured no degradation of samples. The samples were scanned with galvanometric mirrors (Thorlabs). The Raman inelastic backscattered light was collected by the same objective and focused with the microscope tube lens onto the slit of a spectrometer (Andor, Shamrock 303i, grating 300 lines mm^−1^; the slit also acts an effective pinhole for confocal detection). The spectrometer is equipped with a high-sensitivity charge-coupled camera (Andor, iXon 897). The image presented was taken with an integration time per pixel of 500 ms. Recording of data was performed by a custom MATLAB program. For Raman imaging, black phosphorous flakes (300–600-nm thickness; 2D Semiconductors Ltd) were mechanically exfoliated inside a nitrogen glovebox and transferred onto a Si substrate. A 0.15-mm-thick coverslip was placed over the flakes and sealed with epoxy glue to act as an encapsulant. The polarization of the pump and Raman light was not strongly controlled, but simply adjusted to maximize the respective signals.

## Online content

Any methods, additional references, Nature Portfolio reporting summaries, source data, extended data, supplementary information, acknowledgements, peer review information; details of author contributions and competing interests; and statements of data and code availability are available at 10.1038/s41586-024-08563-x.

## Supplementary information

Supplementary Information

## Data Availability

The data that support the plots in this paper and other findings of this study are available at Zenodo (10.5281/zenodo.142116566)^52^.
