# Supplementary material for: Magnetically and optically active edges in phosphorene nanoribbons
Source: Nature. 2025 Mar 12;639(8054):348–53. doi: 10.1038/s41586-024-08563-x (PMC11903315; doi:10.1038/s41586-024-08563-x)
Supplement: Supplementary file 1 — Supplementary Information [file 41586_2024_8563_MOESM1_ESM.pdf]

nature portfolio

https://doi.org/10.1038/s41586-024-08563-x

Supplementary information

Magnetically and optically active edges in phosphorene nanoribbons

In the format provided by the authors and unedited

Supplementary Materials

Magnetically and Optically Active Edges in Phosphorene Nanoribbons

[1]Arjun Ashoka, [2,3]Adam J. Clancy[#], [4]Naitik A. Panjwani[#], [5]Adam Cronin, [6]Loren Picco, [3]Eva S. Y. Aw, [1]Nicholas J. M. Popiel, [1]Alexander G. Eaton, [7]Thomas G. Parton, [3]Rebecca R. C. Shutt, [8]Sascha Feldmann, [1]Remington Carey, [9,10]Thomas J. Macdonald, [11]Marion E. Severijnen, [11]Sandra Kleuskens, [12,13]Loreta A. Muscarella, [5,14,15]Felix R. Fischer, [16]Hilton Barbosa de Aguiar, [1]Richard H. Friend, [4]Jan Behrends, [11]Peter C.M. Christianen, [3]Christopher A. Howard, and [1,16,17]Raj Pandya

[1]Cavendish Laboratory, University of Cambridge, J.J. Thomson Avenue, CB3 0HE, Cambridge, United Kingdom
[2]Department of Chemistry, University College London, Christopher Ingold Building, Gordon St, London WC1H 0AJ, UK
[3]Department of Physics & Astronomy, University College London, London, WC1E 6BT, United Kingdom
[4]Berlin Joint EPR Lab, Fachbereich Physik, Freie Universität Berlin, D-14195 Berlin, Germany
[5]Department of Chemistry, University of California, Berkeley, Berkeley, California 94720, United States
[6] Interface Analysis Centre, School of Physics, H H Wills Physics Laboratory, University of Bristol, Tyndall Avenue, Bristol BS8 1TL, United Kingdom
[7]Yusuf Hamied Department of Chemistry, University of Cambridge, Lensfield Road, Cambridge, CB2 1EW, United Kingdom
[8]Institute of Chemical Sciences and Engineering, École Polytechnique Fédérale de Lausanne, Lausanne, Switzerland
[9]Department of Chemistry and Centre for Processable Electronics, Imperial College London, London, W12 0BZ, United Kingdom
[10]Department of Electronic & Electrical Engineering, University College London, London WC1E 7JE, United Kingdom
[11]High Field Magnet Laboratory (HFML - EMFL), Radboud University, 6525 ED, Nijmegen, The Netherlands
[12]Center for Nanophotonics, AMOLF, Science Park 104, 1098 XG Amsterdam, The Netherlands
[13]Department of Physics and Astronomy, Vrije Universiteit Amsterdam, De Boelelaan 1081, 1081 HV Amsterdam, Netherlands
[14]Kavli Energy NanoSciences Institute at the University of California Berkeley and the Lawrence Berkeley National Laboratory, Berkeley, California 94720, United States
[15]Materials Sciences Division, Lawrence Berkeley National Laboratory, Berkeley, California 94720, United States
[16]Laboratoire Kastler Brossel, ENS-Université PSL, CNRS, Sorbonne Université, Collège de France, 24 rue Lhomond, 75005, Paris, France
[17]Department of Chemistry, University of Warwick, Coventry, UK

#Denotes equal contribution
Correspondence: raj.pandya@warwick.ac.uk

Table of Contents

Supplementary Note 1: Characterizing the reproducibility in length, width and height between PNR batches 3

Supplementary Note 2: Discussion of PNR applications and PNR edge alignment and termination 7

Supplementary Note 3: Additional AFM discussion and characterisation 10

Supplementary Note 4: Discussion of the effect of edge reconstruction, termination on magnetism 16

Supplementary Note 5: Scanning Tunnelling Microscopy 17

Supplementary Note 6: Calculation of volume susceptibility 19

Supplementary Note 7: PNR tangling in solution and validity of the rigid rod approximation for susceptibility fitting 21

Supplementary Note 8: Generating a histogram of PNR volumes 25

Supplementary Note 9: Morphology of PNR films (SQUID, EPR, microscopy and pump-probe spectroscopy) 27

Supplementary Note 10: Additional SQUID magnetometry measurements 33

Supplementary Note 11: Raw background corrected SQUID measurements 37

Supplementary Note 12: Exploration of the PNR degradation mechanism in air 43

Supplementary Note 13: Elemental Analysis of the PNR solution (ICP-OES) 48

Supplementary Note 14: Low Temperature Phase Transition (optical and magnetic data) 50

Supplementary Note 15: Additional EPR magnetometry measurements 54

Supplementary Note 17: Persistence Length Measurements 69

Supplementary Note 18: Single ribbon steady-state and transient optical micro-spectroscopy 71

Supplementary Note 19: Pressure Dependent Absorption Spectroscopy 81

Supplementary Note 20: Ensemble photoluminescence (PL) spectroscopy 83

Supplementary Note 21: Scaling of pump-probe signal with magnetic field 89

Supplementary Notes 22: Electroabsorption Measurements 90

Supplementary Note 23: Additional Impulsive Vibrational Spectra 92

Supplementary Note 24: SQUID Sequences 95

References ***98***

Supplementary Note 1: Characterizing the reproducibility in length, width and height between PNR batches

To ensure consistency between sample batches studied in this work, we performed AFM measurements and analysis on 5 distinct PNR batches all produced using the same protocols detailed in the **Methods** section of the main text. These data from batches (1-5) consist of the height, width and length measurements of 21, 62, 71, 60 and 39 PNRs, respectively. **Figure S1** shows histograms of the length, width and length of PNRs from these batches as well as the published length, width and height distribution of ribbons from the first synthesis[1,2] ('Original'/'OG'). We note here that all samples used in this study are from the same lab and group that produced the 'OG' batch[1,2].

Firstly, the distributions in PNR height, width and length are all remarkably similar in form for the different batches, showing a log-normal shape. To characterise the similarity between batches further we calculate the Jensen-Shannon Divergence (JSD) between a given length/width/height distribution on PNRs and the 'OG' batch[3]. The JSD is a measure of dissimilarity between two probability distributions with values below 0.5 (and closer to 0) indicating similarity. As shown in **Figure S2** for all distributions, we observe JSD values between 0.1 and 0.4 indicating a good level of similarity. To be more quantitative, we can extract the average PNR lengths, widths and layer numbers (mode). Overall, we observe a mean width of 15.8 nm across batches and a modal thickness of 1 layer. This data is summarised in **Figure 1c** of the main text and **Table S1**. Our analysis reveals that over 70% of PNRs we measure will be monolayer in thickness and 90% will have monolayer or bilayer thickness. The distribution of widths is narrow with, on average, 72% of all PNRs being between 10 and 20 nm in width. The average PNR length is 674 nm with 80% being between 200 and 900 nm long. While the widths, lengths and thicknesses appear to be log-normally distributed (**Figure S1**), because it is challenging to know the exact distribution, (because there may be extremely small PNRs that cannot be detected by TEM or AFM) we report a standard deviation simply based on $\sigma = \sqrt{((\Sigma(x_i - \bar{x})^2) / (N - 1))}$ where $\bar{x}$ is the sample mean, x_i is a given sample and N the number of samples.

By showing that the PNRs measured are similar across batches we can: (i) make comparison between the results in this work and the PNRs considered in the original synthesis paper[1,2]; (ii) combine data from multiple PNR batches as there is high-degree of reproducibility between batches, e.g., in **Figure 1c** and; (iii) substantiate our claims on data at the ensemble level as we know we have predominantly monolayers which are 10/15 nm in width. This is important for rationalizing many of our observations from the stability of edge magnetism to our EPR signals.

**Property**	**Batch 1**	**Batch 2**	**Batch 3**	**Batch 4**	**Batch 5**	**OG**	**Overall mean**
N PNRs	21	62	71	60	39	240	n/a
Monolayer	81%	74%	71%	66%	79%	69%	73%
Monolayer or Bilayer	95%	88%	82%	73%	84%	87%	90%
Mean width (nm)	7.6	13.6	12.3	21.5	18.2	20.9	15.8
Mean length (nm)	618	671	782	652	660	682	674

Table S1 Dimensions of PNRs between batches: Summary of PNR thicknesses, widths and lengths for the 5 batches used in this work, alongside the properties of PNRs used in (‘OG’ (original))[1,2].

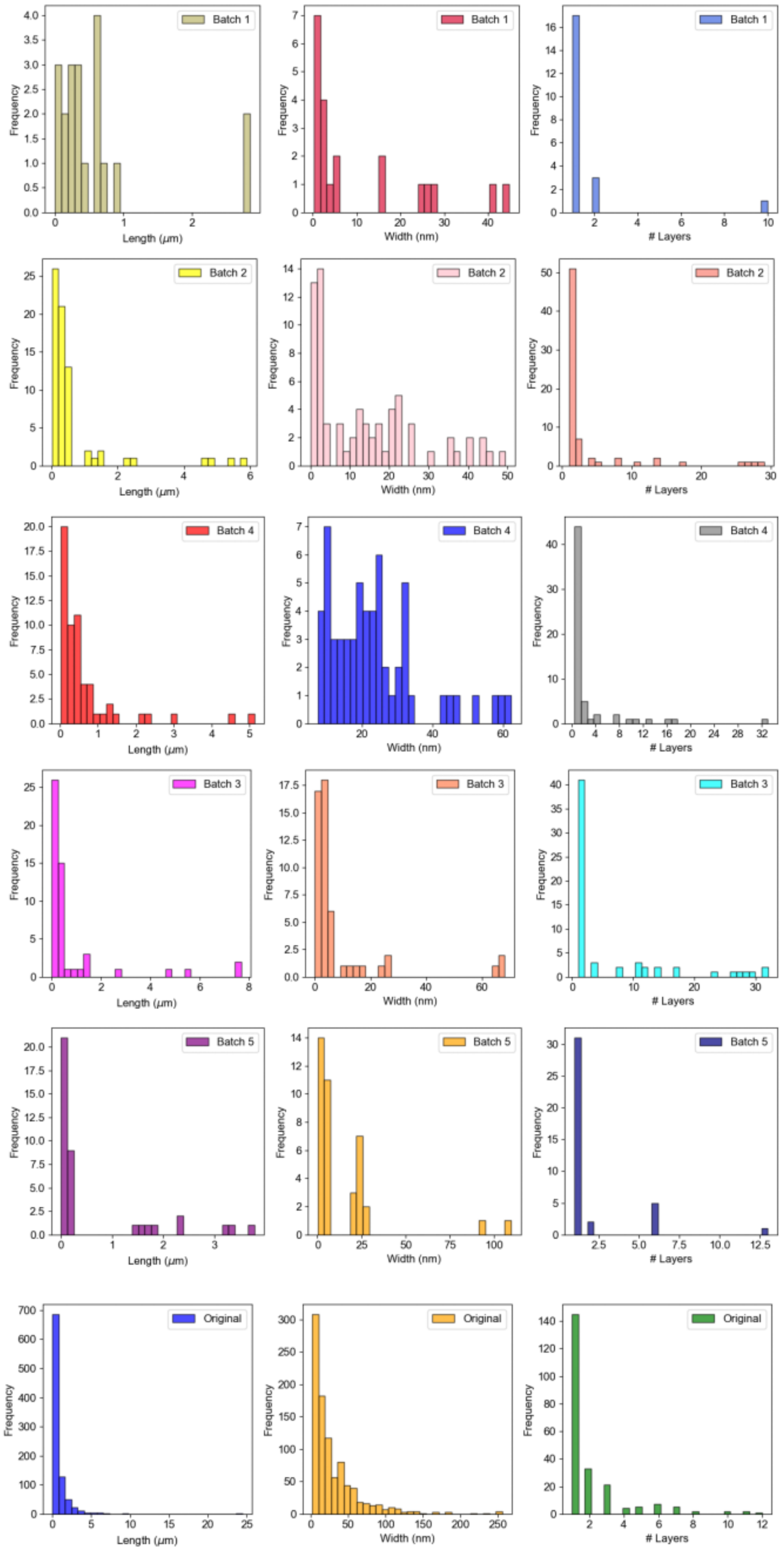
Batch 1
Frequency
Length (μm)
Batch 1
Frequency
Width (nm)
Batch 1
Frequency
Layers
Batch 2
Frequency
Length (μm)
Batch 2
Frequency
Width (nm)
Batch 2
Frequency
Layers
Batch 4
Frequency
Length (μm)
Batch 4
Frequency
Width (nm)
Batch 4
Frequency
Layers
Batch 3
Frequency
Length (μm)
Batch 3
Frequency
Width (nm)
Batch 3
Frequency
Layers
Batch 5
Frequency
Length (μm)
Batch 5
Frequency
Width (nm)
Batch 5
Frequency
Layers
Original
Frequency
Length (μm)
Original
Frequency
Width (nm)
Original
Frequency
Layers

Figure S1 PNR dimensions from different batches in histogram form: Histogram of PNR lengths, widths and heights from 5 different PNR batches used for experiments in this text as well as the PNR samples used in ref[2] ('OG' (original)).

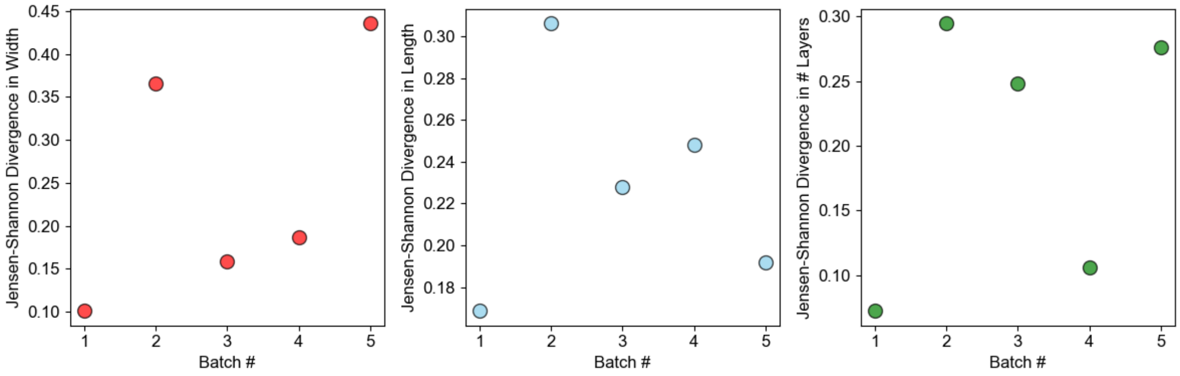

Figure S2 Comparing PNR widths between batches: Jensen-Shannon Divergence (JSD) of PNR batches 1-5 as compared to the original batch synthesised in ref[4].

Finally, for PNRs that are >1 µm in length we characterise the width uniformity by measuring the width in 15 different locations of each PNR with AFM, and then determing the standard deviation in the measured values. As shown in **Figure S3**, along a given PNR the width remains highly homogeneous with very little variation in the standard width deviation which remains around ± 0.73 nm.

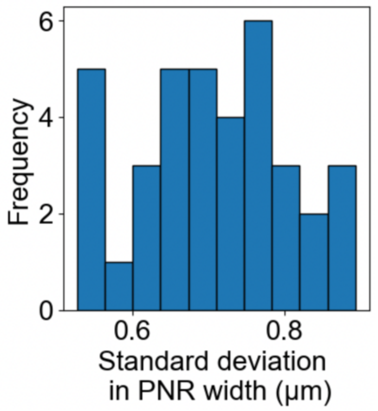

Figure S3 PNR uniformity histogram: Histogram of standard deviation in PNR widths measured. This is calculated by measuring the width in 15 places along each PNR for 37 PNRs that are greater than >1 µm long.

Supplementary Note 2: Discussion of PNR applications and PNR edge alignment and termination

PNR applications

The lengths of PNRs with zigzag aligned edges (produced in batch solution) as demonstrated in this work surpasses that of almost all (edge alignment controlled) GNRs, where lengths are typically limited to 100 nm due to on-surface growth approaches. This is a serious issue for single nanoribbon-based devices (which we demonstrate the first (deposition) steps towards with top-down synthesized PNRs in **Supplementary Note 5**)[5]. And while spectacular results have recently been published on long GNRs[6] we note these remain encapsulated in hBN making their edge characterization/access and application beyond nanoribbon electronics limited. Indeed, fundamentally PNRs are much easier to form with zigzag edges than GNRs due to the lower formation energies for these types of edge in phosphorene based[7] systems compared to those based on graphene[8]. This means for applications that rely on zigzag (aligned) edges in both materials e.g., thermoelectrics, there is a large chance PNRs will outperform GNRs with much higher Seeback coefficients[7]. While outstanding GNR based charge transport measurements have been made[6] there are predications that PNRs can surpass this (with >10^3 on/off ratios for transistors) due to both the higher fundamental hole mobility of the base phosphorene lattice, and the enhanced electronic conduction properties introduced when phosphorene particularly is in nanoribbon form[9–12]. While this remains to be tested there are promising signs with recent experimental reports of PNRs boosting hole transport in perovskite based solar cells[13]. This result could have implications for other optoelectronic devices e.g., photodetectors, which could benefit from PNRs.

With respect to magnetism, while this has been observed in GNRs, it has only been following heteroatom or radical edge sensitization (which is inherently unscalable) as opposed to utilizing only the intrinsic GNR lattice/structure (although this is theoretically possible[14]).

Similarly, while semiconducting properties have been demonstrated in GNRs the coupling to magnetic behavior is also missing (coupling to metallic behavior has only been achieved so far[15]). In contrast, we have demonstrated evidence for coupling between magnetic and optical/electronic properties in PNRs with routes towards further exploration. This is a property that will be key for low-energy computing, spintronic or switching devices based on nanoribbons. One of the biggest (and most surprising) results in this work is that the magneto-optical properties of PNRs can be preserved into thin-films (ensemble). This opens up an even wider range of device possibilities.

There are several emerging applications where PNRs will be useful and potentially superior to GNRs. One is as additives in battery electrodes where their predicted high ionic conductivities (particularly for Na^+) could be useful (GNRs are poor ionic conductors)[16,17]. Similarly theoretical studies in the growing area of active plasmonics[18] indicate doped PNRs could be extremely useful due to their predicted high third order non-linear susceptibilities (10^{-5} esu). For photocatalysis and specifically water splitting the potentially (layer and width) tunebale band-gap of PNRs and high absorption compared to GNRs has meant they have been suggested to be particularly desirable for such applications[19]. Finally, in this work we demonstrate an extremely high pressure/strain tunability for the electronic properties (bandgap) of PNRs (see **Supplementary Note 19**) with a much larger changes per unit of pressure as compared to phosphorene or GNRs. This opens up applications in active electronics such as for strain tunable LEDs, gas sensors and flexoelectronics[20] where the combination of nanoribbon edges, phosphorene's anisotropic lattice (symmetry breaking) and external (temperature, electric field (see **Supplementary Note 22** for influence of electric fields on PNR properties) or magnetic) stimuli could be particularly powerful.

PNR edge alignment and termination

While we are unable to atomically-resolve the PNR edges with methods such as STM (see **Supplementary Note 5**), we are able to confirm the zigzag edge alignment of the PNRs studied. Firstly, in the original paper and thesis reporting the synthesis of PNRs by Watts *et al*.[1,2] (which the synthesis of all PNRs in this study follows) the ribbon alignment was characterised using selected area electron diffraction (SAED). This method allows a diffraction pattern to be taken over a selected real space region (of defined orientation) giving the (reciprocal space) diffraction pattern of that region. Comparison of the two images (and the calculated pattern) is then used to determine the crystal alignment. This measurement was performed on over 40 PNRs in that paper[1,2], from multiple synthesis batches, and without exception the ribbons were always aligned in the zigzag direction. The rationale for this is that '*rapid diffusion of alkali metal ions along the corrugated channels, [result in] strain between intercalated and unintercalated regions, therefore [causing] the longer P-P bonds to break in the zigzag direction*'. This mechanism for zigzag ribbon formation was further supported by Raman data, which revealed that ribbionisation of the lithiated black phosphorous crystal was in the zigzag direction[1] supported by other publications, which had characterised structures formed after intercalation of alkali ions into bP[17,21,22]). We also note that alternative top-down methods, based on electrochemical intercalation/exfoliation, for producing PNRs all result in zigzag-aligned PNRs (explained as due to the relative weakness of the longer P-P bonds[17,22]).

Polarised Raman has been shown to be sensitive to zigzag *vs* armchair edges[23] via the different relative intensity between broken-symmetry edge modes. However, these modes are generally low in intensity and our IVS measurements, which are best placed to probe these modes, have low spectral resolution meaning they cannot distinguish precisely between B_{1g} and B_{3g}^1 modes.

Instead, we turn to A_g^1 mode, which as commented by Ribiero *et al*.[23], should only appear in a configuration where the excitation light and detection polarisations are orthogonal; quoting from this work: *'The most significant anomalous signals in the XZ configuration are observed at the zigzag edge for the A_g^1 mode…'*. Hence, if the edges are indeed zigzag (terminated), then the impulsive polarized Raman spectroscopy we perform (in **Supplementary Note 23**) should reveal this. As shown in **Figure S72** the A_g^1 mode is indeed significantly more strongly observed in the orthogonally polarized pump-probe configuration; in our case HV refers to the short and long axis of PNRs which is equivalent to XZ in ref[23]. This provides evidence that a zigzag-like edge termination is present in the PNRs we study. We show the real-space motions associated with above modes, as calculated and demonstrated by Ribiero *et al*.[23], below.

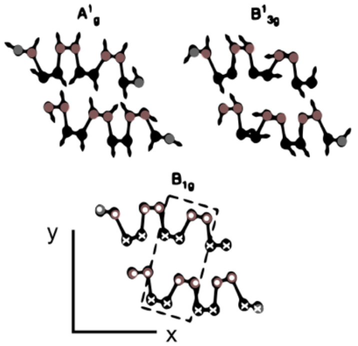

Figure S4 Real space picture of A^1_g, B^1_{3g} and B^1_g modes for a phosphorene slab: Given the similarity in mode frequencies between PNRs and phosphorene it is likely similar modes and motions exist in PNRs. The 'x' represents a vibration out of the page and 'o' into the page (see also Ribiero *et al*[23]).

It is important to state that this strong polarisation dependence on the Raman properties of black P/phosphorene, which PNRs have a near identical Raman spectrum to, has been confirmed both experimentally and theoretically[24]. Furthermore, arguments using polarised impulsive Raman spectroscopy have been used by Mao *et al.* to confirm zigzag terminations in black P/phosphorene in a similar manner to us[25]. We also re-emphasise, as in the main text, that theoretical calculations of PNR properties typically refer to zigzag aligned and terminated edges.

Nonetheless, we believe also further work is required to unequivocally characterise the zigzag edge in PNRs using e.g., STM (see **Supplementary Note 5**). It is for this reason we refer to the PNRs we study as zigzag aligned as this has been shown beyond doubt. In **Supplementary Note 4** we discuss potential reconstructions of the PNR edge.

<u>Width effects</u>

Theory calculations suggest that PNRs can host ferromagnetic (FM) or antiferromagnetic (AFM) ground edge states based on their width. At narrow widths <3 nm the AFM state is lowest in energy and dominates whereas as the width increases FM states become more favourable (or in some calculations AFM and FM configurations become degenerate)[26–28]. However, in general, regardless of the calculation methods (DFT or Quantum Monte Carlo) the conclusion from the theory literature on PNRs is that as the width of PNRs increases >12 nm there is less influence on the magnetic properties. As our ensemble is dominated by PNRs of width around 15 nm (see Supplementary Note 1), we suggest that our PNRs are in a crossover range where width effects start to play less of a role. More specifically in this range (as predicted from Quantum Monte Carlo and dynamic mean field theory calculations) we will be in a crossover between decoherence-dominated classical magnetism and fluctuation-dominated quantum magnetism. This is as also observed in graphene nanoribbons[4,29]. It will be interesting avenue to investigate *experimentally* the link between PNR width and magnetism. But this will require however producing size separated PNRs in the 3 – 10 nm range, where width effects are expected to be strongest, using local magnetic probes (nanodiamond AFM, scanning tunnelling microscopy, nano-MOKE) and calculations which extended to larger width PNRs.

Supplementary Note 3: Additional AFM discussion and characterisation

High-speed (HS) AFM PNR uniformity measurements

To extract uniformity histograms for the PNR detailed in **Figure 1** of the main text, the pixels in the HS-AFM data corresponding to the ribbon were automatically isolated from those of the background HOPG via a bespoke image analysis algorithm containing local thresholding and edge detection steps. Once separated, a histogram of HOPG and ribbon heights (in 0.05 nm intervals) was created and Gaussian fits to each distribution were calculated. The mean height of the HOPG was subtracted from the mean height of the ribbon to establish the relative height of the ribbon from the background. This height was found to be 0.58 nm with a standard deviation of 0.14 nm, from a sample set of more than 3,700 pixels. This height is very close to the expected layer thickness of 0.54 nm for phosphorene[1] and is indicative of a monolayer ribbon. This exact same procedure was used to identify the heights of all other PNRs in this work

The distribution in the uniformity as shown in **Figure 1** of the main text specifically arises from the uncertainty in the measurement convoluted with the intrinsic width of a PNR across its length. As the mean height of the ribbon taken from the histogram is ~0.6 nm, it is unlikely that we are seeing stacking of PNRs because this measured height is very close to the 0.54 nm measured for monolayer phosphorene ribbons reported previously as well as earlier reports in monolayer phosphorene[1,2,30].

All heights are measured relative to the local background HOPG. We have further deemed it unlikely that the ribbon in **Figure 1a** has anything beneath it because the underlying HOPG matches the appearance and surface roughness of bare, freshly cleaved HOPG from other control measurements. We apply the same logic when assessing if any PNRs are lying on top of one another in other AFM data.

We note that we present many more height values in the histogram than the width histogram in **Figure 1a** of the main text. That is because the height histogram is made up of all the pixels from the top of the ribbon whereas the width histogram consists of all the width measurements possible along the ribbon. In other words there are many more height pixels along each slice of the ribbon's width than there are width slices. Thus, the height and width samples have very different numbers of values because we are measuring a single ribbon and unless it is essentially 1 pixel wide we will always have more height measurements than width measurements.

Stacking and Bundling

In films prepared for AFM measurements we do not observe any restacking of the PNRs. For ribbons that do have heights greater than that of a monolayer (i.e., multilayer PNRs) we observe that they are always uniform in width consistent with single multilayer ribbons rather than re-stacked/overlaying examples of multiple thinner ribbons deposited on top of one another. In other words we do not find PNRs aggregated when cast from dilute solutions suggesting they are not aggregating in solution. However, given their length they may necessarily overlap upon deposition.

We do note that we observe some splits at points along multilayer ribbons into two ribbons of the same width but lower layer numbers (i.e., a 5-layer ribbon splitting at a Y-junction into 2- and 3-layer ribbons; see discussion around Figure 2 in ref[2]). Regarding bundling, the AFM data does not demonstrate any bundling/twisting/coiling of ribbons about one another. A library of AFM images containing multiple PNRs in each image is provided in **Figure S5**.

Roughness

Surface roughness measurements are very dependent on a range of operator/user factors, as discussed by Petr Klapetek (Chief AFM Metrologist at the Czech Metrology Institute and co-developer of Gwyddion, the gold standard open-source AFM data analysis software) in his recent paper[31]. Our approach is detailed below.

The HOPG surface roughness was typically measured in ten different locations and averaged. The images were *not* flattened line-by-line to prevent the underestimation reported in ref[31]. The total surface area measured to produce the final value was always around 3.48 μm^2 with a pixel resolution of 0.39 nm^2. This equates to 8.9 megapixels collected in this 3.48 μm^2 area and provides enough oversampling to accurately reflect the roughness. The mean roughness for the datasets used in this work was found to be 0.693 nm ± 0.045 nm. Given that the HOPG surface also has probable contamination from the NMP or DMF solvent, we also took a roughness measurement from pristine and freshly-cleaved HOPG. This measurement resulted in a value of 0.206 nm ± 0.020 nm.

We can compare the values we obtain for HOPG roughness we obtain from recent measurements in the literature: 0.5 nm ± 0.2 nm (ref[32]), ~0.5 Å (ref[33]), 0.07 nm ± 0.02 nm (ref[34]). Comparing our results with these three recent examples highlights the variation in these measurements (due to a variety of operator and environmental factors). However, we are confident in the validity of the measurement approach we have taken and that our measurements are not subject to the typical underestimation that is mentioned in ref[31] due the use of line-by-line flattening techniques. This means the height and PNR layer thicknesses we report are robust to HOPG-background subtraction artifacts.

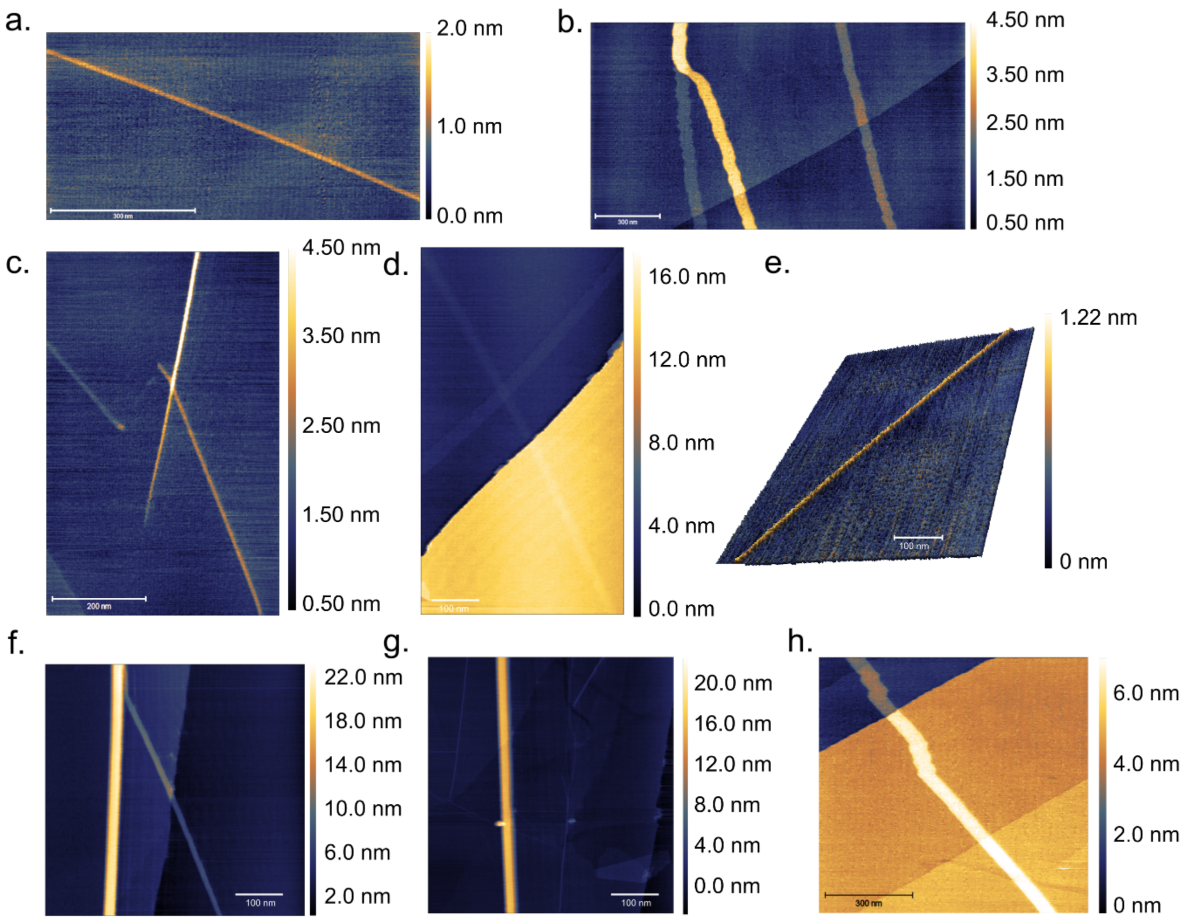

Figure S5 Ensemble AFM images of PNRs on HOPG: a. 1 monolayer (scale bar is 300 nm). **b.** 2 monolayer PNRs (far left and right) and bilayer in centre (scale bar is 300 nm). **c.** 3 monolayer PNRs (scale bar is 200 nm). **d.** 2 monolayer PNRs (scale bar is 100 nm). **e.** 1 monolayer PNR (scale bar is 100 nm). **f.** 5-layer PNR (left) and monolayer PNR (right) scale bar is 100 nm. **g.** Multilayer PNR (scale bar is 100 nm). **h.** Monolayer PNR (scale bar is 300 nm).

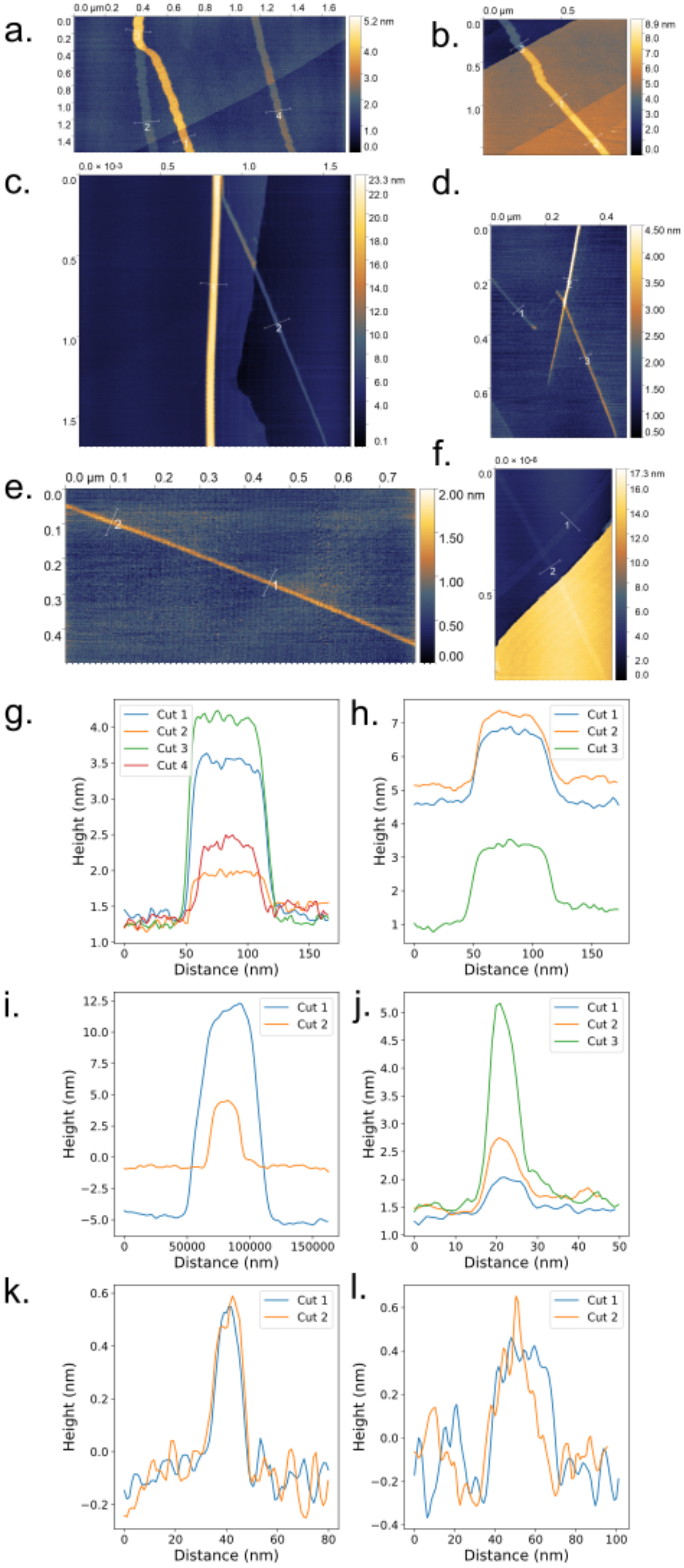
a.
b.
c.
d.
e.
f.
g.
h.
i.
j.
k.
l.
5.2 nm
8.9 nm
23.3 nm
4.50 nm
2.00 nm
17.3 nm
Cut 1
Cut 2
Cut 3
Cut 4
Height (nm)
Distance (nm)

Figure S6 AFM images with line-cuts: a-f. Selected AFM images from Figure **S5** with positions marked where linecuts are taken. **g-l.** Line cuts from marked images **a-f.**. Graph **g** corresponds to image **a**; graph **h** corresponds to image **b**; graph **i** corresponds to image **c**; graph **j** corresponds to image **d**; graph **k** corresponds to image **e**; graph **l** corresponds to image **f**.

<u>Conformation to surface</u>

We also find that PNRs conform very well to large steps in the underlying HOPG substrate as shown in **Figure S7** and **Figure S8**, clearly demonstrating that the ribbons tend to bend very uniformly without any twisting through the step.

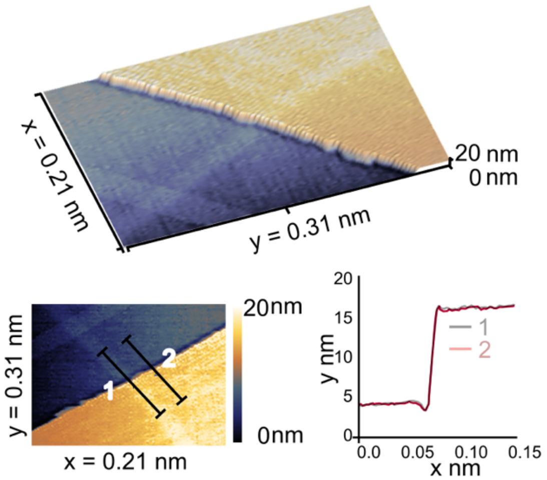

Figure S7 AFM of a PNR conforming to a HOPG surface and associated line-cuts: (Top) AFM of PNR (white silhouette) lying atop of HOPG substrate (yellow). (Bottom) Line profiles show the ribbon conforms almost perfectly to the HOPG step-edge.

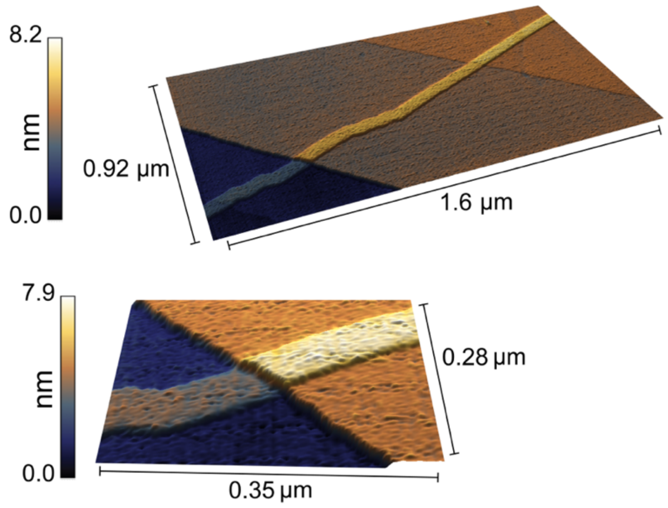

Figure S8 AFM of a PNR conforming to a HOPG surface: AFM of 4-layer PNR (gold silhouette) that is 10 nm wide which climbs 3 nm steps in the HOPG substrate (orange) with extremely close conformity.

Supplementary Note 4: Discussion of the effect of edge reconstruction, termination on magnetism

We can use theory and literature measurements on black P/phosphorene to make a tentative discussion about the edge reconstruction in PNRs and the effect of this on their properties. While PNRs, with non-reconstructed edges (from a pristine zigzag configuration), have been suggested to have a band gap, this gap is extremely small without edge reconstruction[35]. Scanning tunnelling microscopy/scanning tunnelling spectroscopy (STM/STS) and DFT calculations suggest that reconstruction of the edge widens the PNR bandgap bringing it to the visible spectral range as we experimentally observe. In this case, there is self-passivation of dangling bonds at the phosphorene edges which results in a switch of the phosphorous coordination from 3 to 5 or 3 to 4 (ref[35]; in this paper PNRs and phosphorene edges are treated interchangeably which is (theoretically) reasonable also for the widths we consider[36]). Further, calculations suggest the 5-coordinate state to be the most energetically stable termed a ZZ[Tube] reconstruction[37] but this remains experimentally unproven. We also note that from the STM of phosphorene in ref[35], 'armchair reconstructions' of the edge were ruled to be energetically unfavourable.

The above reconstructions also imply that there are no dangling bonds at phosphorene edges, which have been theoretically suggested to be necessary for magnetism[27,38,39]. Indeed, a 5-coordiante P for example would result in the loss of a lone pair and the movement of an unpaired electron into a bonding orbital. Hence, the exact reconstructions as detailed above are unlikely to be exactly occurring in the PNRs we study.

What we can more confidently state is that the presence of the edge phonon mode we observe has been theoretically shown not to be a consequence of just broken translational symmetry but emerging as a result of also edge reconstruction. In this case the suggestion is that the edge reconstruction that gives rise to this mode is 'subtle' and involves: '*the stretching and contraction of the puckered structure along the length [such] that the distance between the projected atoms clearly varies in the xy plane…[and] a marked change in the atomic displacement vectors*' (ref[23]). This edge reconstruction involves the maintaining of dangling bonds. Given the fact that we clearly observe the edge phonon mode described in the above reconstruction, we can suggest that edges of PNRs may involve puckering of P atoms along the edge as described in ref[23].

Supplementary Note 5: Scanning Tunnelling Microscopy

Imaging the edge of PNRs is non-trivial, requiring STM or (S)TEM measurements. High resolution (S)TEM attempts have been thwarted by beam damage. Moreover, the fact PNRs preferentially deposit around, rather than over, holes in TEM grids means the amorphous support interferes with edge measurements because the support is much thicker that the ribbon itself.

Consequently, bond resolved STM is the only choice for imaging PNR edges. Indeed, for graphene nanoribbons (GNRs) there have been numerous individual papers over the last 15 years devoted to this single measurement technique and data[40–42]. The GNRs that are often characterised by STM are also cyclo-dehydrogenated *in situ* on the Au (111) STM substrate required for imaging. In the case of the PNRs we have a top-down synthetic approach, which makes this route unfeasible. In other words, even before preforming STM, a principal challenge is understanding how to deposit the solution-processed PNRs for such measurements. We emphasise this is a non-trivial problem and one that will be required to be overcome if we are to locally study edge effects in individual PNRs and further optimise single PNR devices based on top-down synthesised PNRs. Our STM measurements represent the first step towards this.

All STM experiments were performed on a commercial Omicron LT-STM at 4.2 K using PtIr STM tips. Samples were prepared by aerosolizing PNRs dispersed in NMP onto a HOPG substrate to minimize aggregation on the surface. The deposition parameters and the concentration/density of PNRs on the surface were optimized using ambient AFM imaging. For UHV-STM experiments PNRs were aerosolized onto a freshly cleaved HOPG substrate using an Iwata Custom Micron CM-C airbrush. The coated substrate was immediately transferred into the vacuum chamber and annealed for >1 h at 150 ˚C before transferring the substrate to the STM sample stage (extended annealing or heating >150 ˚C led to partial degradation of PNRs).

Locating PNRs on the surface using STM proved challenging due to the limited range of the *xy*-piezo stage of the STM head when compared to ambient AFM and the low density of PNRs on the surface. Furthermore, the STM probe is very sensitive to residual solvent molecules the PNRs are dispersed in. Since imaging quality quickly degrades while scanning on PNR samples, frequent tip preparation had to be performed on a separate clean Au(111) substrate. Features with dimensions commensurate with PNRs were encountered on the surface, yet it remained ambiguous whether these were PNRs which had been deposited onto the surface or could be attributed to grain boundaries/defects in the HOPG. A low signal to noise ratio and broadening of the features when reducing the scan area can be attributed to the collection of surfactant molecules on the tip. Large clusters were occasionally observed as illustrated in **Figure S9a**. Occasionally longer straighter features were observed which may have been few-layer (**Figure S9b**) or single layer (**Figure S9c**) PNRs averaging roughly 0.5–0.7 nm in height. Atomically resolved imaging with CO functionalized STM tips on or along the edges of these structure was unsuccessful (**Figure S9d**) with Moiré effects with the underlying HOPG substrate that could not be removed, e.g., by background subtraction in the Fourier domain. The above results highlight the challenge of bond-resolved imaging of PNRs but provide a first step towards this and how to deposit PNRs for atomic level characterisation.

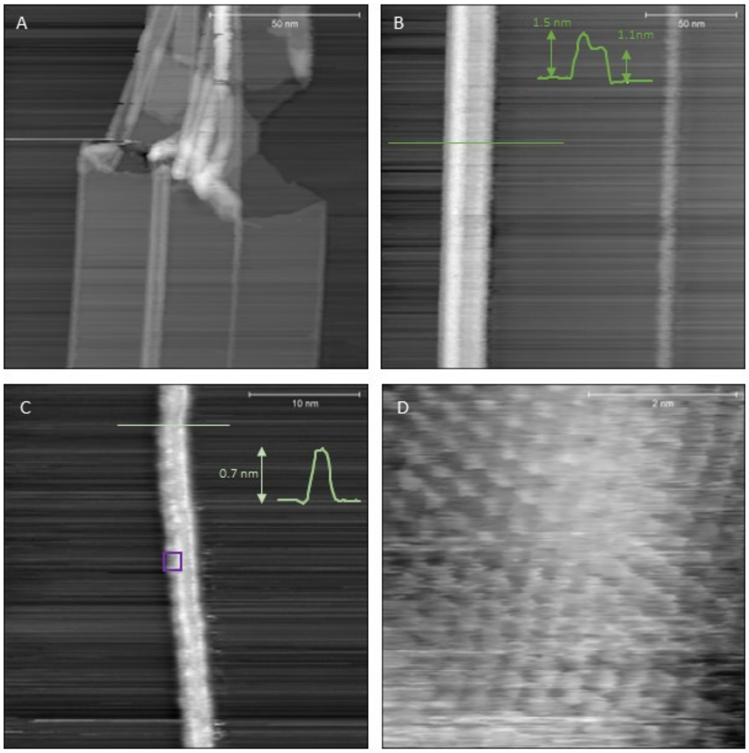

Figure S9 STM imaging of PNRs: a. STM image of a PNR cluster accumulated on a HOPG step edge. **b-c.** STM images of suspected few-layer and single layer PNRs, with line profiles corresponding to the cross sections shown in the image. **d.** Close-up STM image of the area in the purple box in (**c**). Bias voltage and current used for images is: V_s = 500 mV and I_t = 20 pA, respectively.

Supplementary Note 6: Calculation of volume susceptibility

For both diamagnetism and paramagnetism the magnetic moment induced by an external magnetic field B is given by,

$$\boldsymbol{m} = \frac{\chi V}{\mu_0} \cdot \boldsymbol{B} \text{ (Equation 1)}$$

The magnetic interaction energy is given as the projection of the magnetic moment along the applied field direction, i.e.,

$$\boldsymbol{E(B)} = -\int_0^B \boldsymbol{m} \cdot \boldsymbol{dB} = -\frac{\chi V}{2\mu_0} \boldsymbol{B}^2 \text{ (Equation 2)}$$

Where $\chi \approx 10^{-3}$ with χ positive for paramagnetism and $\chi \approx - 10^{-5}$, with χ negative for diamagnetism. For a magnetically anisotropic material with in-plane isotropy, χ can be represented as,

$$\chi = \begin{pmatrix} \chi_{\perp} & 0 & 0 \\ 0 & \chi_{||} & 0 \\ 0 & 0 & \chi_{||} \end{pmatrix} \text{ (Equation 3)}$$

The magnetic moment and energy along the two directions are therefore

$$\begin{cases} \boldsymbol{m}_{\perp} = \dfrac{\chi_{\perp} \boldsymbol{V} \cdot \boldsymbol{B}}{\mu_0} \quad \Rightarrow \quad \boldsymbol{E} = -\dfrac{\chi_{\perp} \boldsymbol{V}}{2\mu_0} \boldsymbol{B}^2 \\ \boldsymbol{m}_{||} = \dfrac{\chi_{||} \boldsymbol{V} \cdot \boldsymbol{B}}{\mu_0} \quad \Rightarrow \quad \boldsymbol{E} = -\dfrac{\chi_{||} \boldsymbol{V}}{2\mu_0} \boldsymbol{B}^2 \end{cases}$$

There is therefore an energy associated with the anisotropy of the magnetic susceptibility χ called the orientational anisotropy energy,

$$\boldsymbol{\Delta E}_{\mathbf{an}} = -\frac{(\chi_{||} - \chi_{\perp}) V}{2\mu_0} \boldsymbol{B}^2 = -\frac{\Delta \chi V}{2\mu_0} \boldsymbol{B}^2 \text{ (Equation 4).}$$

For a material with optical anisotropy measured through the magnetic birefringence, the birefringence is assumed to measure the degree of alignment S,

$$\boldsymbol{S} = \frac{3 \cdot \langle \cos^2 \boldsymbol{\theta} \rangle - 1}{2} \text{ (Equation 5)}$$

where,

$$\langle \cos^2 \boldsymbol{\theta} \rangle = \frac{\int \cos^2 \boldsymbol{\theta} \cdot \boldsymbol{f(\theta)} \cdot \sin \boldsymbol{\theta} \cdot \boldsymbol{d\theta}}{\int \boldsymbol{f(\theta)} \cdot \sin \boldsymbol{\theta} \cdot \boldsymbol{d\theta}} \text{ and } \langle \cos^2 \boldsymbol{\theta} \rangle = \frac{\int \cos^2 \boldsymbol{\theta} \cdot \boldsymbol{f(\theta)} \cdot \sin \boldsymbol{\theta} \cdot \boldsymbol{d\theta}}{\int \boldsymbol{f(\theta)} \cdot \sin \boldsymbol{\theta} \cdot \boldsymbol{d\theta}}.$$

Hence by fitting the magnetic birefringence as a function of applied field, we can determine the volume susceptibility χ_V. Further the field required to align the PNRs at room temperature depends on the volume of the PNRs,

$$\frac{\Delta \chi V}{2\mu_0} \boldsymbol{B}^2 \geq \boldsymbol{k_B T} \text{ (Equation 6)}$$

Finally, we note in the main text we compare the anisotropy of PNRs to black phosphorous. The latter has its predominant magnetic anisotropy between the in- and out-of-plane directions, which are not expected to be the dominant anisotropy directions in PNRs[43].

Supplementary Note 7: PNR tangling in solution and validity of the rigid rod approximation for susceptibility fitting

PNRs could potentially bundle/tangle in solution at the concentrations investigated and it is important to establish the degree to which this is present. To assess first how this influences the optical properties we perform absorption measurements (**Figure S10**) in solution as a function of concentration as previously outlined by Niu *et al*. as a method to assess the degree of ribbon tangling[44]. We note that whilst the absorption spectrum (like for Niu *et al*.[44]) is relatively featureless, no changes in the spectrum are observed as a function of concentration. We confirm that this absorption does not arise from the scattering, by performing photothermal deflection spectroscopy (PDS) measurements (in **Figure S10a**); PDS is a scatter free technique. Secondly, we measured the decay of the photoinduced absorption at 600 nm (pumping with a 480 nm pump pulse; 100 fs) as a function of concentration. We find that the lifetime of the decay remains approximately independent of the concentration (**Figure S11**) in the following PNR:DMF ratios tested: 1 mg:1 ml, 1 mg:4 ml, 1 mg:10 ml and 1 mg:50 ml. The above results suggest that the optical properties of PNRs are not strongly influenced by any bundling effects in the concentration regime that we study. Finally, transient absorption experiments on PNR films of different thickness do not show any change in the PNR excited lifetime as shown below in **Figure S12**, again suggesting the overlap of PNRs does not significantly influence the results.

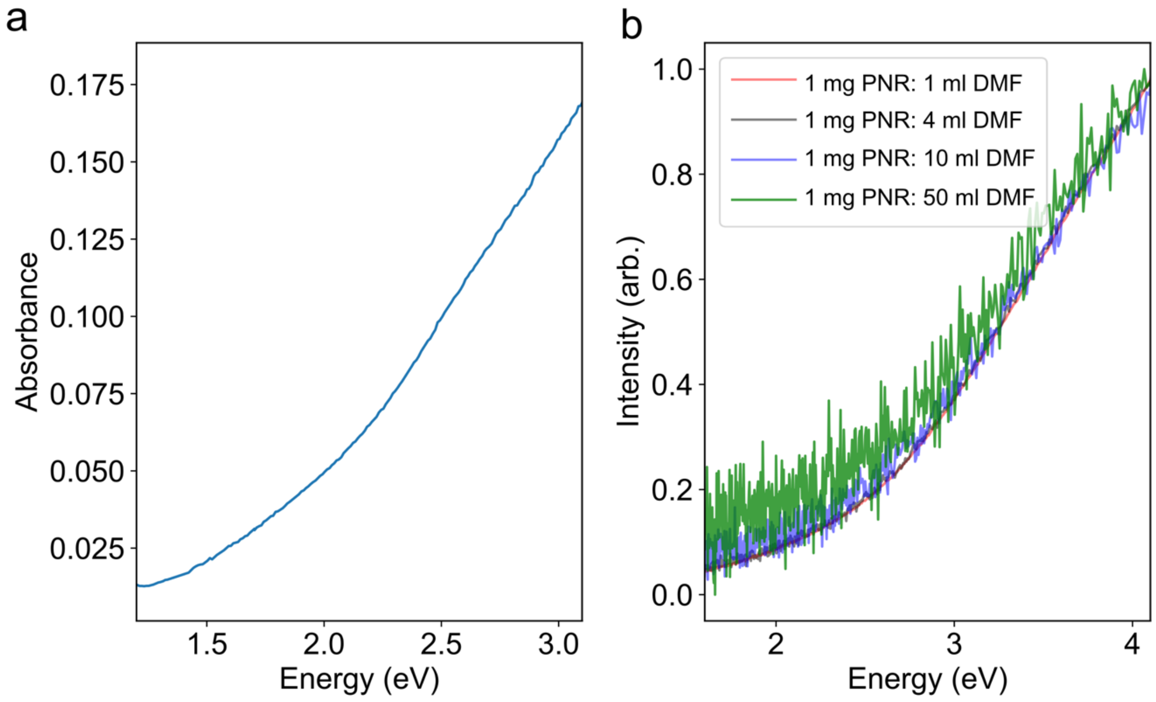

Figure S10 Scattering and concentration dependence of PNR absorption: a. Photothermal deflection spectrum (PDS) of PNR thin film. PDS is a scatter free absorption technique and confirms that the absorption does not arise from scattering. **b**. UV-Vis spectrum of PNRs in solution as a function of concentration. No change in the optical properties are observed suggesting that aggregation does not strongly influence the optical properties. The PNR:solvent concentration ratios are indicated in the legend.

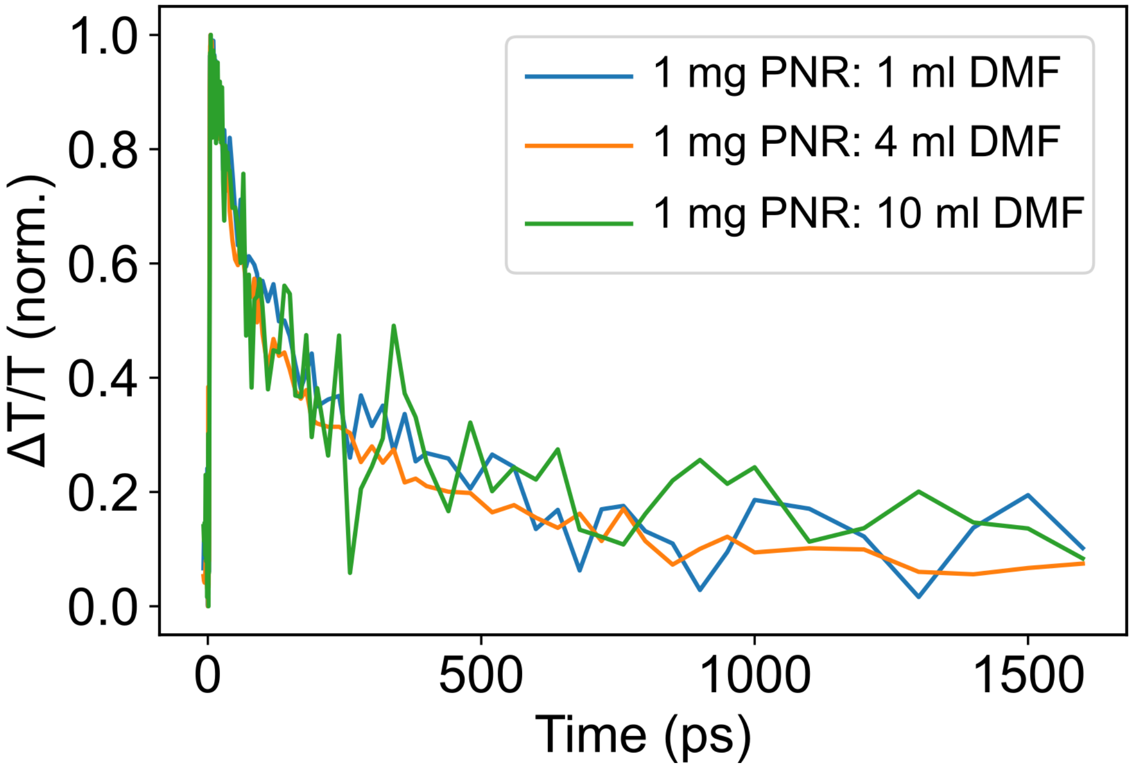

Figure S11 Concentration dependence of time-resolved dynamics of PNRs: Decay of photoinduced absorption band at 600 nm as a function of PNR concentration.

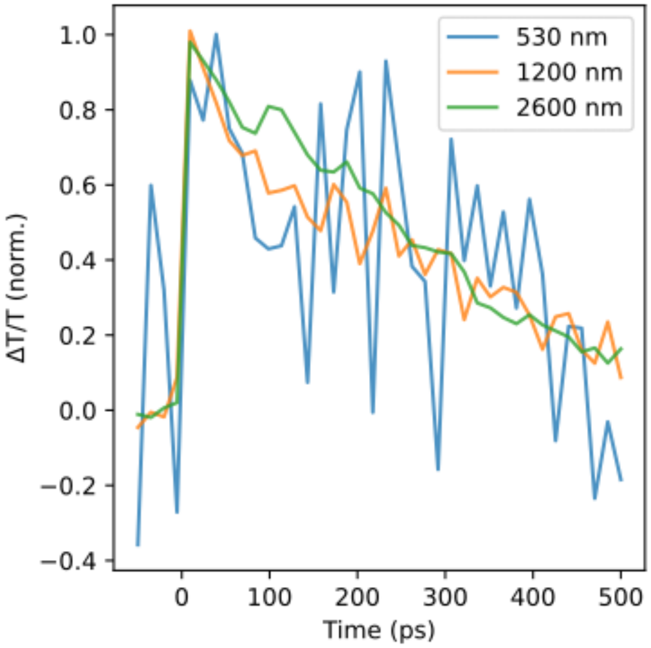

Figure S12: Decay of photoinduced absorption band at 600 nm as a function of PNR maximum film thickness. The film thickness is the maximum found across the film by AFM imaging of step edges across the inhomogeneous sample and typically varies by ± 300 nm.

In terms of the optical properties of PNRs we do not observe any effects directly of bundling/embedding. As we show in Supplementary Note 7 (**Figure S10** and **Figure S11**) the absorption spectrum of PNR solutions does not change with concentration (a method for testing for nanoribbon bundling as outlined in Niu *et al*.[44]), nor does the excited state lifetime of PNRs change. While there is some overlap between the PNR absorption and emission at room temperature the quantum yield of emission is only 11% and hence any energy transfer e.g., through FRET is expected to be low.

We note that comparing the optical properties of single PNRs with that of the film there are some differences. For instance, as we highlight in **Figure S54** there is a range of emission linewidths within the ensemble. However, this arises not from stacking effects but due to the range of PNR widths. Similarly, as shown in **Figure S59** the photoinduced absorption band of PNRs shows a range of lifetimes depending on the exact PNR, but this we again attribute to dimensionality effects. Indeed, as we show in **Figure S63**, dense and dilute regions of PNRs show near identical PL spectra except with slight difference in the ratio of intensities of the emission bands. If significant stacking/bundling effects were taking place, we would expect the spectra to be shifted in energy from one another due to the different dielectric environment experienced by PNRs that are overlapped *versus* those that are isolated. This is not the case suggesting again overlapping of PNRs does not influence our observations strongly. We do note that the difference in intensity ratios between the PNR PL bands in the ensemble spectra in **Figure S63** could arise due reabsorption effects in thicker films; we now comment on this in Supplementary Note 20.

To *qualitatively* assess the behaviour of PNRs in solution we have performed dynamic light scattering measurements (DLS; all DLS performed with a Malvern Instruments Zetasizer Ultimate and analysed with custom codes). Here, we measure the intensity correlation function $G_2(\tau) = B[1 + \beta|g_1(\tau)|^2]$, where $G_2(\tau)$ is the measured intensity correlation function capturing changes in scattering intensity, $g_1(\tau)$ is the electric field correlation function describing particle motion, τ is the correlation time and β a fitting a parameter. The above equation can be rearranged to a normalised correlation function $C(\tau) = \beta e^{-2\Gamma\tau}$ where Γ captures the shape and diffusion of particles within a solution (amongst other things) via $\Gamma = D\,[\frac{4\pi n}{\lambda}\sin\left(\frac{\theta}{2}\right)]^2$ where D is the diffusion coefficient, n the refractive index of the solution, θ the scattering angle and λ the probe wavelength. Interestingly, a plot of the $C(\tau)$ *vs* τ shows a broad but mono-modal distribution in PNR sizes from DLS. This agrees with our AFM and SEM studies, but we note that the DLS measurements are likely insensitive to large PNRs and are challenging to quantitively interpret for such a complex non-spherical material. Nonetheless, based on this observation we chose to fit an equation of the form $ln\frac{G_2(\tau)-B}{B} = \ln(\beta) - 2\Gamma\tau + K_2^2\tau^2$ where $\frac{k_2}{\Gamma^2}$ is the polydispersity of the sample (see ref[45]). Performing this fit gives a polydispersity value of 0.21 and an apparent diffusion coefficient of D_{app} = 1.2 × 10^{-9} cm^2 s^{-1}. Importantly the plot of Γ *versus* q = $[\frac{4\pi n}{\lambda}\sin\left(\frac{\theta}{2}\right)]^2$ has a non-zero y-axis intercept, an indicator of anisotropic objects in DLS (see ref[45]). Commenting on the exact nature of this anisotropy is not possible for the PNRs in solution presently without polarised DLS measurements (or static light scattering) and further modelling, but the fact that a shape anisotropy is strongly present in the solution suggests PNRs retain a ribbon-like shape in solution and do not curl into sphere-like objects for examples. We note that the polydispersity from DLS while relatively small (0.05 – 0.2 values are considered small with our chosen model for polymeric objects, see ref[45]) requires careful modelling/theory for full interpretation which is beyond the scope of this work. We hence do not interpret it or relate it to the ratio of line widths for single PNRs *vs* the ensemble in transient absorption or emission imaging as done in **Supplementary Note 18**.

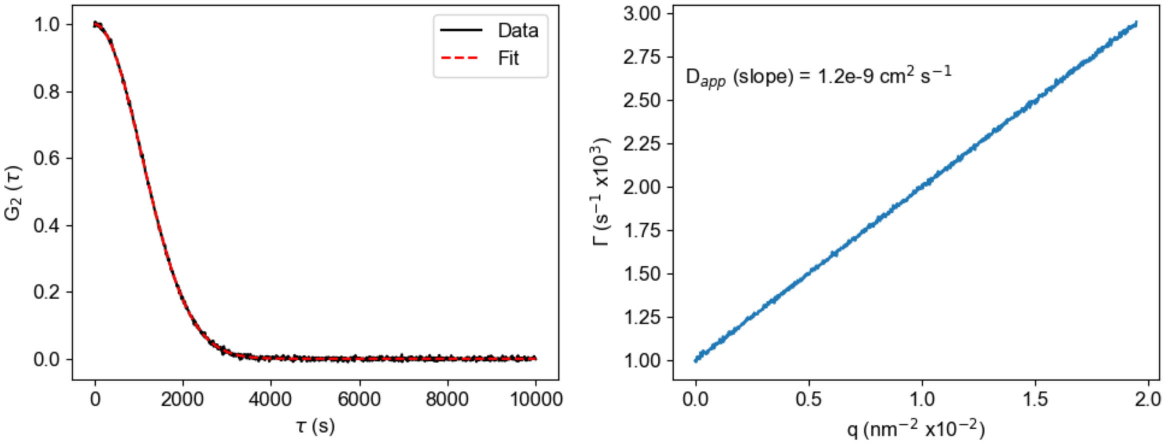

Figure S13 Dynamic light scattering of PNR solutions: (Left) Intensity correlation function measured using DLS along with associated quadratic fit as detailed in the text above. (Right) Γ *versus* q (= $[\frac{4\pi n}{\lambda}\sin\left(\frac{\theta}{2}\right)]^2$) plot obtained from DLS parameters. From the slope the apparent diffusion coefficient of objects in the solution can be obtained. The non-zero *y*-intercept indicates anisotropic particles are being examined.

The above data provides evidence that although PNRs are flexible they retain a ribbon like shape in solution (i.e., little curling into tubes or bending into spherical like shapes).

Supplementary Note 8: Generating a histogram of PNR volumes

In **Figure 1c** of the main text, we have used the extensive, previously-reported histograms of ribbon heights (from AFM), widths (from TEM) and length (from TEM)[2] to generate a volume distribution of the ribbons as shown below. Our PNRs are produced by the same group and using the exact same method as those in ref[2].

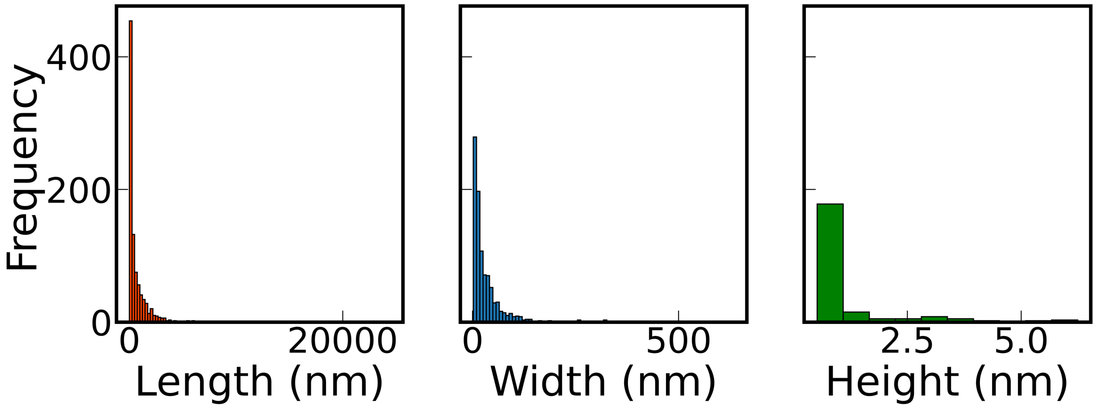

Figure S14 PNR dimensions studied in Watts *et al.*[2]: Length, width and height histograms of the PNRs measured in the original study of Watts *et al.*[2].

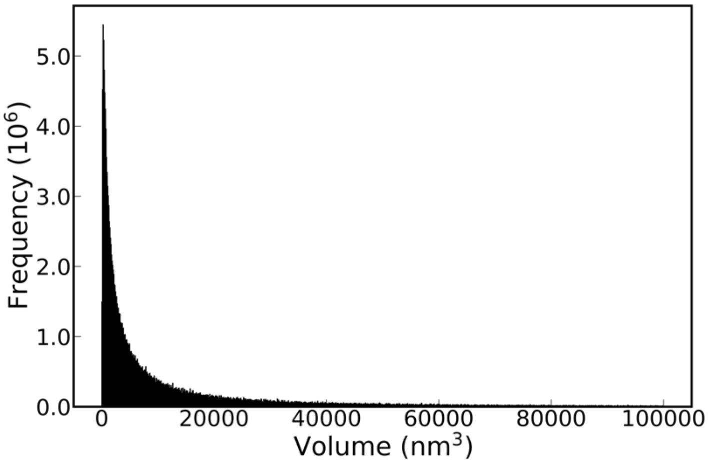

Figure S15 Histogram of PNR volumes extracted from the data in Watts *et al.*[2]: Using the histograms in **Figure S14** we can generate an uncorrelated volume histogram of the PNRs by iterating over all possible height, width and length combinations of PNRs.

We estimate the ribbon volume histogram in **Figure 1c** by assuming the simplest possible case of entirely uncorrelated length (from TEM), width (from TEM) and height (from AFM) distributions. Our volume histogram therefore samples a much larger space than any of the individual measured histograms by considering all possible height, width and length combinations of PNRs based on the measured histograms of these values. Because our magnetic birefringence measurements are at the ensemble level, this approach allows us to most safely estimate the range of $\Delta\chi$ for PNRs. While this approach results in a wide range of $\Delta\chi$ values, all values we obtain are still at least one order of magnitude larger than $\Delta\chi$ for black P, indicative of a true edge-effect as we describe in the main text. Furthermore, new AFM measurements in this study,

where the length, width and height are always measured on the same PNRs (correlated), yield a volume distribution comparable to the uncorrelated volume distribution. This data set has a lower number of points and lower lateral resolution as compared to the uncorrelated data. This is due to the reduced throughput and lateral resolution of AFM as compared to the TEM which was used to obtain the length and width dimensions for the uncorrelated data. Hence, we use the previously-published uncorrelated volume histogram to estimate our $\Delta\chi$ distribution. We note that as we show in **Supplementary Note 1** the batch-to-batch reproducibility of PNRs is exceptionally high and the length/height/width distributions match very well with the previously published data of Watts *et al.*[2], i.e., combining data sets between different PNRs batches is valid. In general, given that persistence length of PNRs is longer than the average ribbon length (see **Supplementary Note 17**) it is unlikely height, width and length values are strongly correlated. Our approach is hence technically robust and allows a truly statistical picture of the susceptibility in the magnetic anisotropy to be obtained.

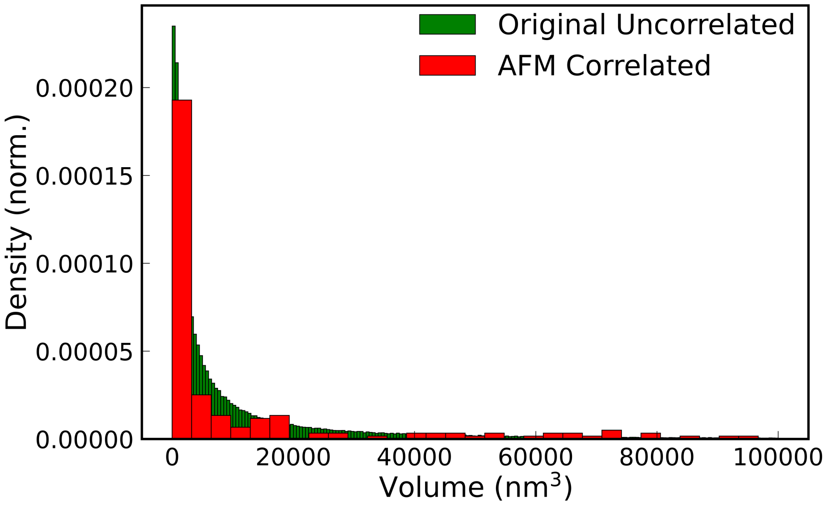

Figure S16 PNR dimensions measured in Watts *et al.*[2] compared with those measured here: A comparison using a smaller sample size (253 PNRs) where the width, length and height measurements are always on the same PNR (measured using AFM) *versus* an uncorrelated volume distribution with the lengths, widths and heights of PNRs measured from both TEM and AFM across different ribbons. There is a strong overlap between the two histograms.

Supplementary Note 9: Morphology of PNR films (SQUID, EPR, microscopy and pump-probe spectroscopy)

The concentration of the PNR solutions from which drop casting is performed to create solid-state samples is nominally 1 ± 0.1 mg/ml unless otherwise stated (uncertainty is across repeat measurements). This concentration is determined by drop casting a known volume of PNR solution onto a pre-weighed coverslip and then measuring the differential increase in mass. Typically for the SQUID measurements 400 μl of PNR solution was drop cast into a plastic straw (**Figure S17a** shows a picture of the sample). Resulting in a total mass of PNRs of 400 μg. We have also performed background subtracted SQUID measurements using a quartz rod. The same volume of PNRs as for the plastic straw is drop cast onto these rods (**Figure S17b**).

For the solid-state SQUID measurements, once drop cast in the plastic straw/on the quartz rod, the solution is dried under vacuum to leave a 'spot' of sample which has the 'structure' of a local dipole needed for SQUID. In the case of the quartz rod, we managed to constrain the solution of the PNRs along the length of the quartz rod by using parafilm wrapped around the quartz rod on both sides of the PNR deposition zone. We then remove the parafilm carefully, using acetone to clean the quartz rod where PNRs and the parafilm are in contact. All depositions and preparations were performed in an inert N_2 environment.

To characterise the morphology of the plastic straw samples, a film was prepared as above and the straw was cut into 'flat' segments to allow SEM measurements to be performed. As we could not place the quartz rods in the SEM (or break them into segments) we prepared PNRs on borosilicate glass coverslips with comparable volumes of solution used in SQUID and then characterised the films with SEM/AFM measurements. We note AFM measurements on plastic shards was not possible.

As the SEM images in **Figure S18** show, the plastic/quartz samples for SQUID show no preferential alignment or structure of PNRs. Due to the low atomic number of phosphorus, the contrast of PNRs in SEM is quite low and we are therefore mainly sensitive to thicker (multilayer) PNRs which are >80 nm in width, with the monolayer, 10-20 nm width PNRs that dominate the sample, blurred out in the images. Measuring the thickness and roughness with AFM over 6 step edges for the samples prepared on borosilicate glass to mimic the quartz rod SQUID samples, we find a mean thickness of 7 ± 1 μm and average roughness of 1.15 μm (**Figure S21a**). We note the roughness in a given position is taken as the standard deviation of the AFM thickness across a 10 μm region of the sample in that location unless otherwise stated.

Because we also perform a number of measurements on borosilicate/quartz/glass substrates e.g., transient absorption, Raman, PL, etc, we also characterise the films on this substrate. In this case, 100 μl of PNRs was drop cast onto a borosilicate coverslip substrate and then dried under vacuum over a period of hours in an Ar glove box. The borosilicate substrate is kept flat whilst drying leading to an inhomogeneous film with a typical 'coffee ring' like drying pattern.

The SEM morphology here is similar to that for the SQUID samples above (**Figure S19**) but the thickness of the PNR film is more variable in this case ranging between 1.5 μm and 3.5 μm measured over 8 locations. The roughness variation is slightly larger than the SQUID samples at between 0.25 and 1.25 μm depending on the location. For the thin films samples the detailed inhomogeneity should not influence optical or magnetic measurements beyond the absolute magnitude of the signal between locations, which we do not compare.

With regards to the inner wall EPR sample (picture in **Figure 17c**), we also perform AFM and SEM measurements to characterise the film morphology. Samples are prepared as for EPR with approximately

400 μl drop cast into a quartz EPR tube (NORELL 4 mm inner wall diameter) with the tube then kept upright and dried under Ar to produce an inhomogeneous inner wall sample as for EPR. The tube was then broken and the film on the shards characterised.

The SEM images in **Figure S20** once again demonstrate a highly inhomogeneous set of films for the EPR samples. Qualitatively, we do observe, by eye, some preferentially orientated 'drying patterns' of the PNR films inside the EPR tube which could contribute to the observation of an orientation dependent EPR signal, but we do not comment further on the nature of this. Again, while for the EPR samples it also appears that wider/thicker PNRs tend to lie at the top of the film and are more prevalent, we are cautious to conclude anything from this because it is most likely due to the limitations of SEM resulting in the narrower PNR monolayers being unresolvable.

To measure the thickness of the EPR inner-wall samples, we again locate step edges with the glass, on shards of broken inner-wall films. Over 5 shards (from the same inner-wall film; **Figure S21c**) we find a mean thickness of 6.3 ± 2.1 μm. This large variation in thickness and roughness (0.75 μm to 4 μm) is in-line with our notion that the inhomogeneity in the inner wall samples results in the observed orientation dependence of the EPR signals.

In the above case atomic force microscopy measurements were done in tapping mode (Veeco Dimension 3100) at room temperature. The AFM cantilever was provided by MikroMasch. The tip radius was ~10 nm. SEM images were obtained using a MIRA3 TESCAN SEM system, at 5 kV.

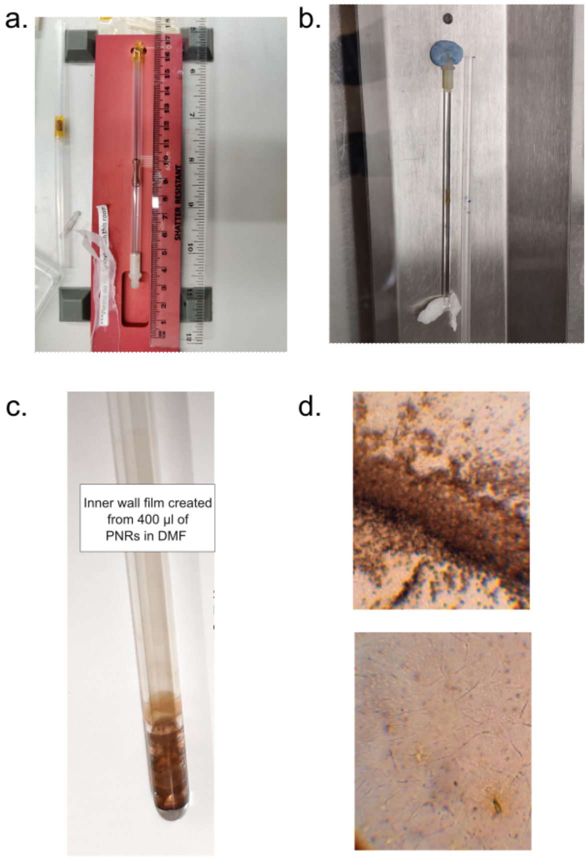

Figure S17 Photographs of PNR samples studied: a. Digital photo of samples as prepared for SQUID measurements where a uniform thin film is made on the inner wall of a plastic straw using 400 μl of solution (left and right). To mimic a point dipole the thin film is made to extend in width as small as possible, limited by movement of the solution drying in the vacuum. **b.** An alternative approach to fabricating samples for SQUID measurements was to coat PNR solution onto a quartz rod. This was again with 400 μl solution with an aim of creating uniform films, with a small surface area ('point dipole'). **c.** Digital photo of 'inner wall' sample used in measurements presented in **Figure 2**. **d.** Image of drop cast (100 μl) PNRs on borosilicate glass substrate showing 'coffee ring' effect where PNRs are distributed randomly across the sample.

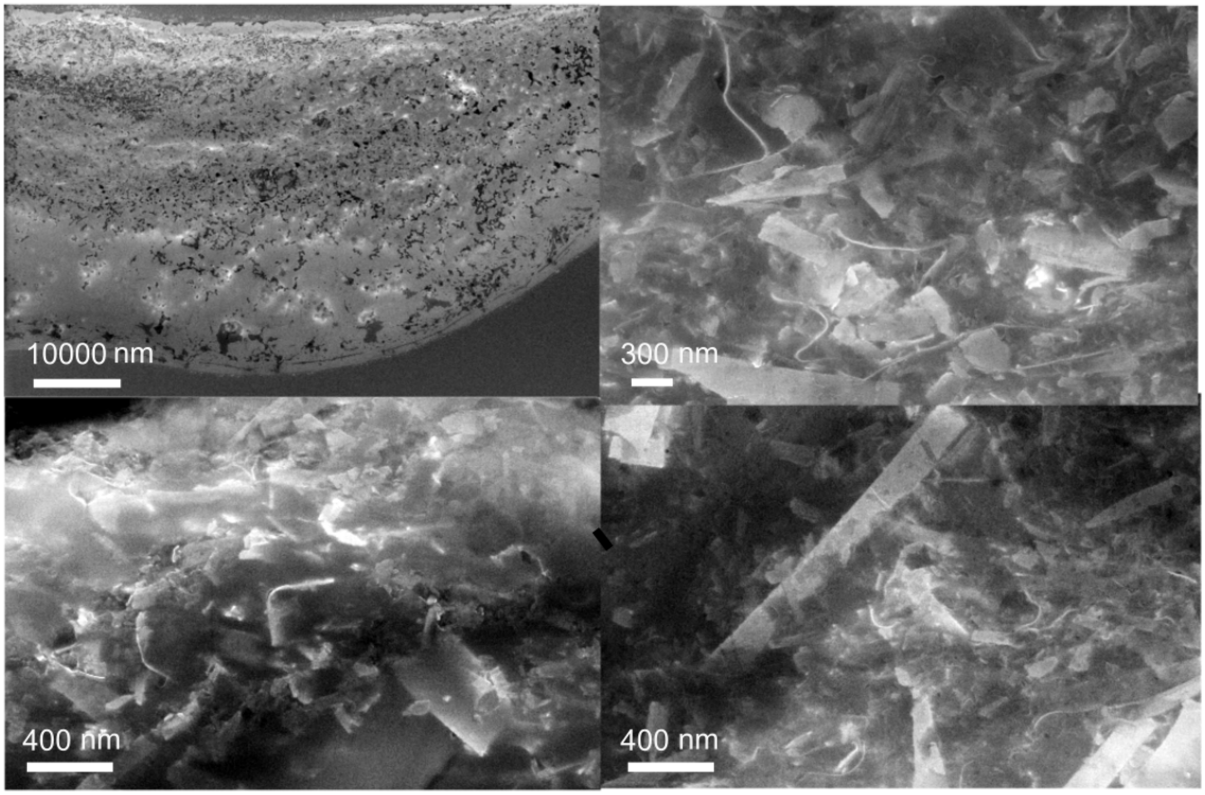

Figure S18 SEM images of PNR samples studied for SQUID: SEM images of PNRs on plastic capsule (top row) or borosilicate glass substrate (bottom row) as prepared in the same manner as for SQUID measurements.

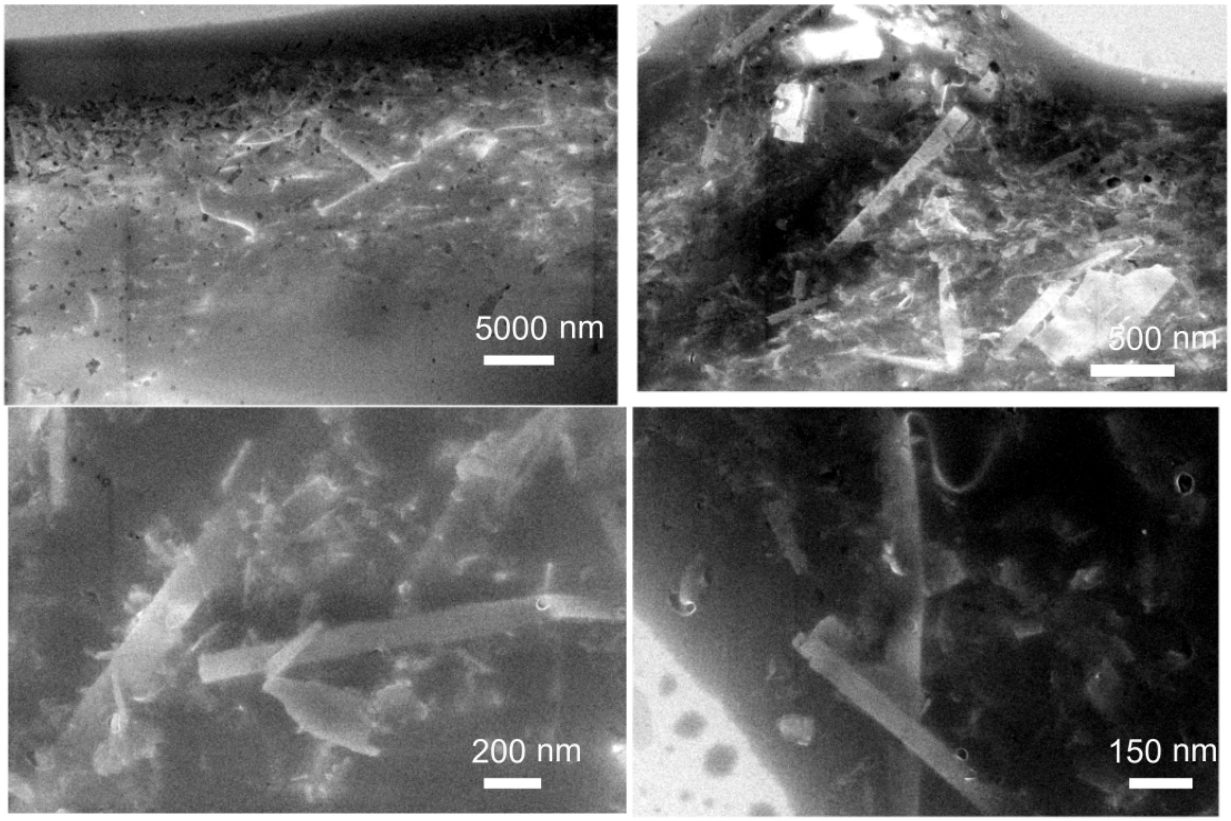

Figure S19 SEM images of PNR samples studied for optical measurements: SEM images of PNRs on borosilicate glass substrate as prepared for optical measurements.

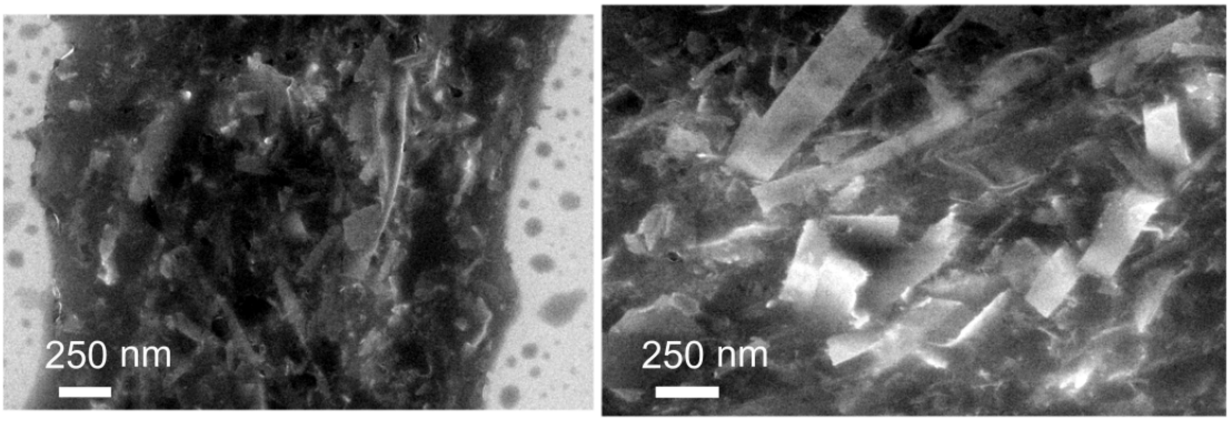

Figure S20 SEM images of PNR samples studied for EPR: SEM images of PNRs on quartz prepared in the same manner as for EPR.

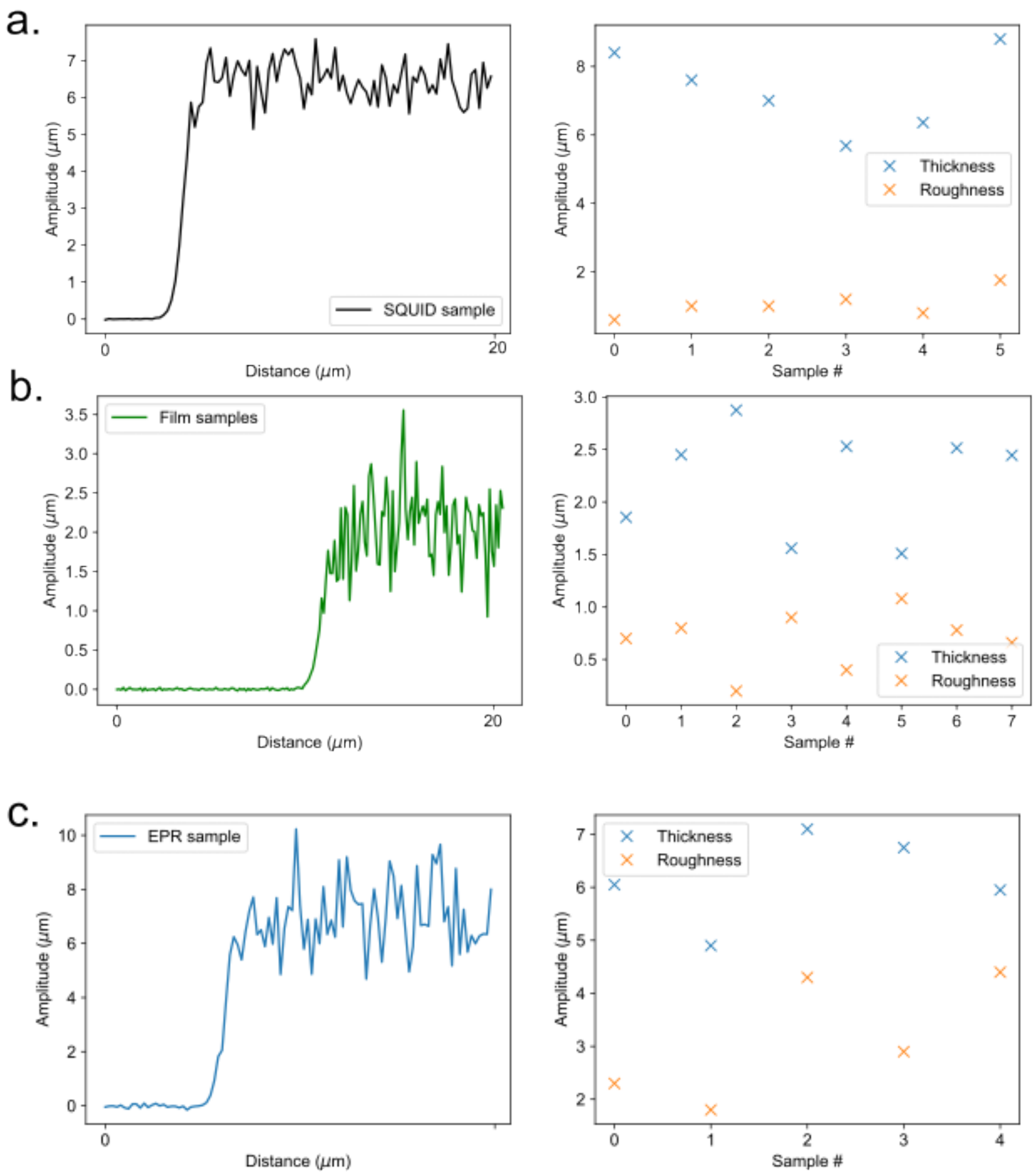

Figure S21 Thickness and roughness characterisation of PNR samples studied with SQUID, optical spectroscopy and EPR: a. Substrate (borosilicate) and tilt corrected AFM line profile along 20 µm section of a PNR film as prepared for SQUID (i.e., same volumes and drying procedure). The film thickness is of 6-7 µm and roughness of 0.8 µm. Right shows distribution of thickness and roughness values measured across 6 different samples/locations. **b.** Substrate (borosilicate glass) and tilt corrected AFM line profile along 25 µm section of a PNR film, as prepared for optical measurements, on glass showing a film thickness of 2-3 µm and roughness of around 1 µm. Right shows distribution of thickness and roughness values measured across 7 different samples/locations. **c.** Substrate and tilt corrected AFM line profile along 20 µm section of PNR film as prepared for EPR showing a film thickness of around 7 µm and roughness of around

2 μm. Right shows distribution of thickness and roughness values measured across 5 different samples/locations. In all cases the roughness is taken as the standard deviation of the AFM thickness across different 10 μm regions of the sample.

We note if we take the SEM images in **Figure S18** (*e.g.*, top right-hand image) and estimate the width of the resolvable PNRs we find a mean width of 176 nm (much larger than the ~20 nm reported from AFM). However, in an area of PNRs of 4 μm^2 this is only 13 resolvable PNRs. Estimating the area of those PNRs and comparing this with the total image area we find they make up only 9.1% of the image. Repeating the same analysis for the SEMs in **Figure S18-S20**, we find 16.3% of the image is made up by these 'large' PNRs on average. This is in-keeping with our estimates from AFM which suggest that >80% of the ensemble is made of monolayer PNRs with widths around 20 nm. In other words, while the above analysis is simplistic, it demonstrates that the AFM and SEM data are roughly consistent in terms of PNR dimensions.

Bundles of PNRs are not discarded in our AFM images but we do not observe any PNRs that are stacked. This is because for AFM measurements we are depositing from dilute solutions so that we can readily resolve individual (isolated) PNRs. In thicker films, from more concentrated solutions, we cannot well resolve individual PNRs in our AFM images and can only capture the film roughness with this technique. In these thicker films PNRs are overlapped, but the influence of this on the magneto-optical properties is not significant.

Supplementary Note 10: Additional SQUID magnetometry measurements

Projecting Field Dependent ZFC and FC Magnetisation

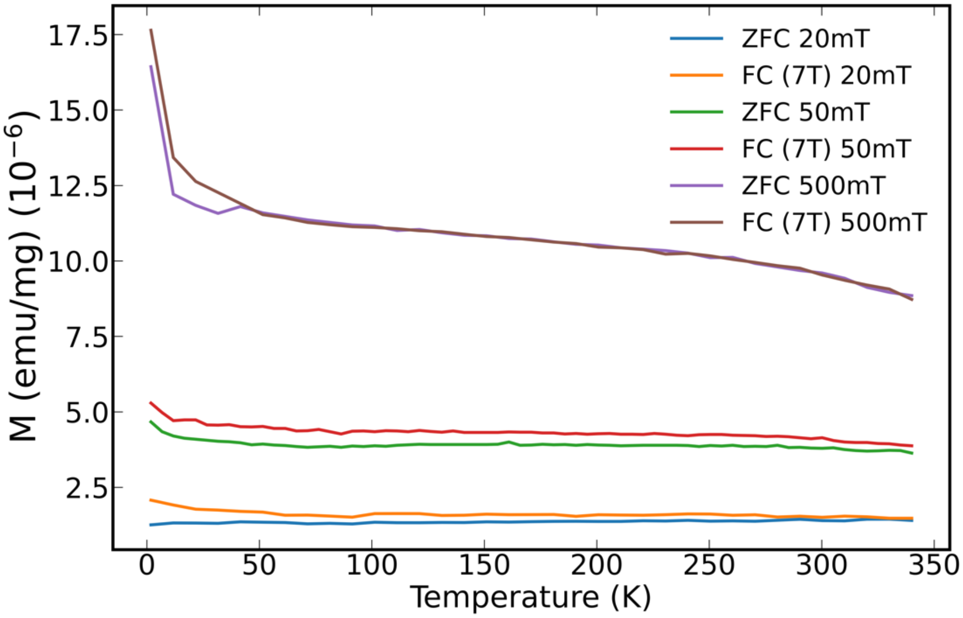

Figure S22 Field and Zero Field Cooled Magnetization as a function of projecting field: Zero field (ZFC) and Field cooled (FC) at 7 T magnetization measured in different projecting fields to demonstrate the sensitivity of the PNRs to the projecting field. If the projecting field is above the coercive field (>50 mT), the room temperature magnetic phase ceases to exist, likely due to the projecting field polarizing all the spins during the measurement. At 50 mT and 20 mT however, it can be seen that the ZFC and FC curves begin to approach each other towards room temperature, but do not yet overlap, signifying that the room temperature magnetic phase can only be measured with very low projecting fields.

SQUID of PNRs Frozen in Solution

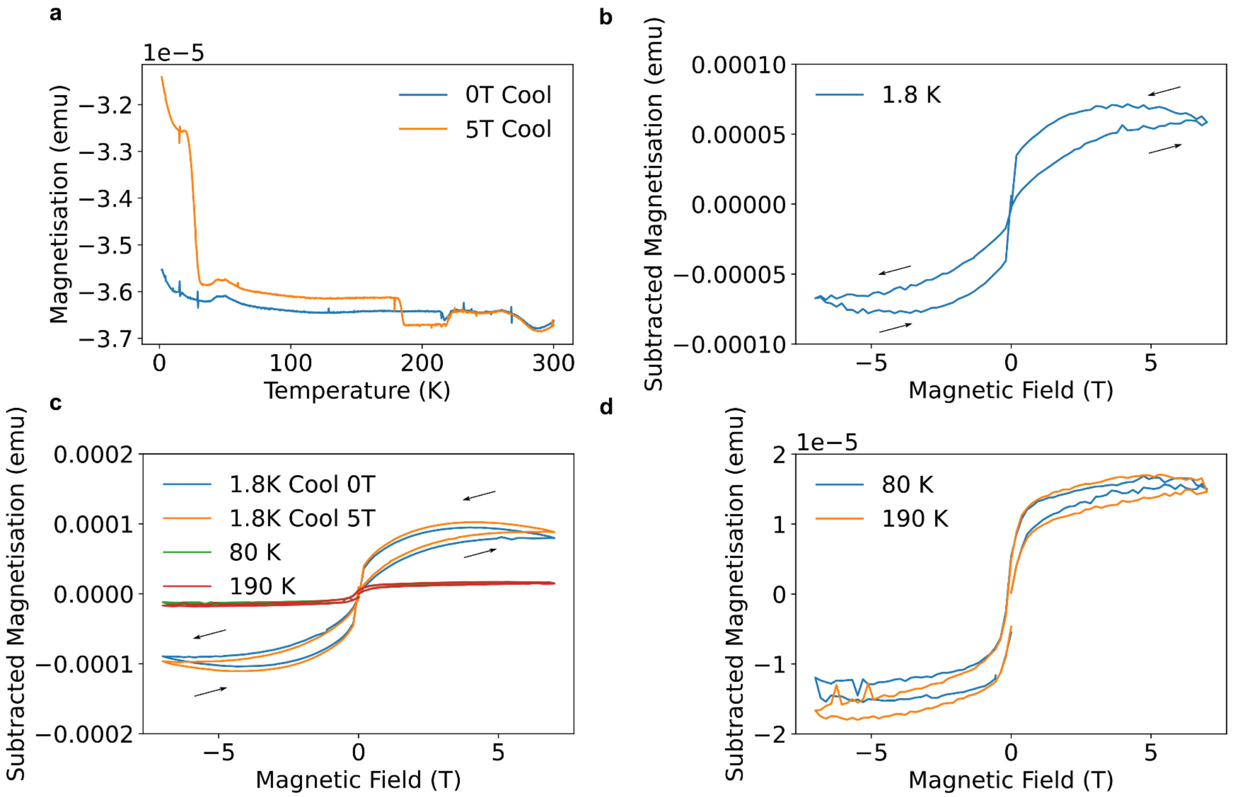

Figure S23 SQUID measurement of PNRs in solution (DMF and NMP): a-d. In both DMF (**a-b**) and NMP (**c-d**) below the solvent freezing points (212 K DMF; 250 K NMP) the motion of the ribbons in solution is frozen out. Hysteresis is then observed in the isothermal magnetization sweeps and field and zero field cooled magnetization sweeps which are split, up to the freezing point of the solvent. The arrows in the panels show the direction of the field sweep.

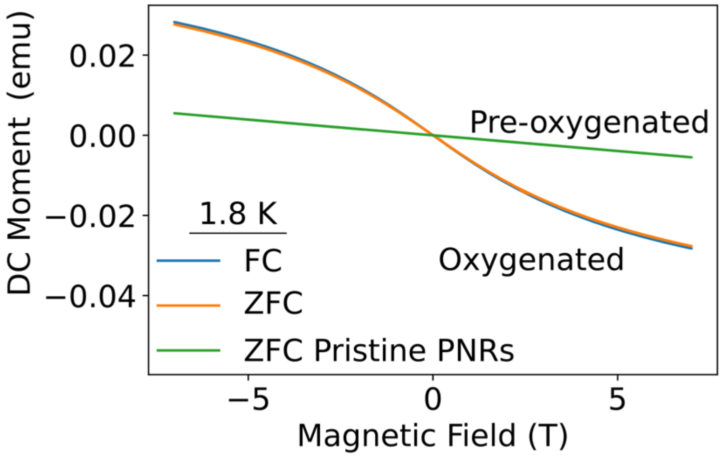

Figure S24 Increased diamagnetic background after exposure of PNRs to air: Upon exposure to air (for ~20 mins) the diamagnetic background of the PNRs can be seen to increase (blue and orange curves). This diamagnetic background in the un-oxygenated scenario (green line) arises from the solvent and is subtracted by fitting the high field limits to straight lines to retrieve the PNRs behavior. All measurements are done at 1.8 K and the FC/ZFC legend indicates whether the sample was field cooled or zero field cooled before the M *vs* H measurement.

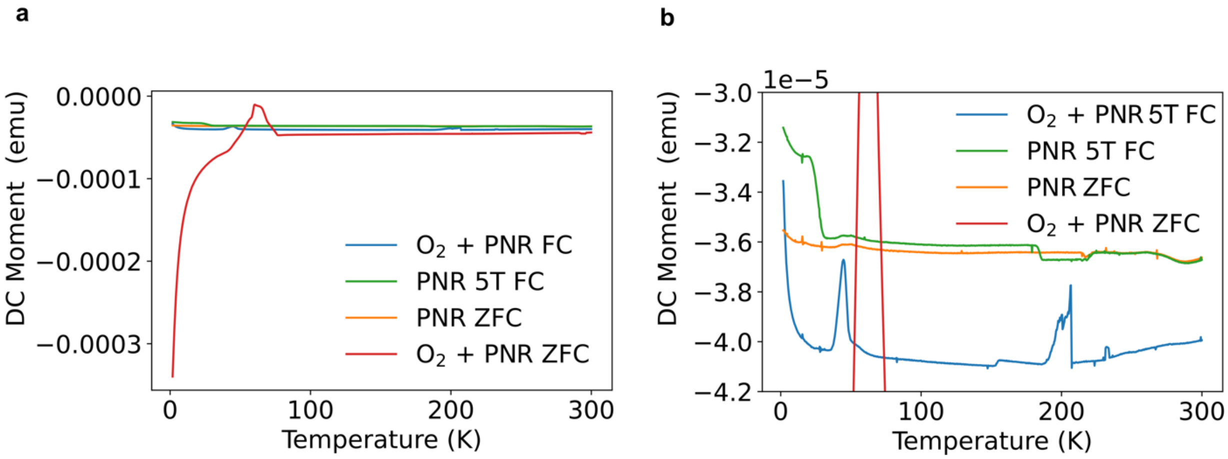

Figure S25 SQUID measurement of PNRs in solution before and after exposure to O_2 (air): a-b. ZFC/FC response of PNRs in solution before (green and orange) and after (blue and red) exposure to O_2 (air), after diamagnetic background subtraction. The increased magnetic response after exposure to air suggests that the magnetic behavior of the PNRs themselves has been altered with an increase in the background diamagnetism which likely comes from the reacted PNRs. Panel (b) is a zoom in on (a).

SQUID of Empty Straw

In the main text, the reason we chose to measure the PNR samples deposited straight into the inner wall of a plastic straw was due to very large diamagnetic background contributions when we attempted to use plastic capsules, etc, where the amount of plastic varies strongly with SQUID position, hence giving a large DC diamagnetic component for the SQUID to pick-up. We found that a bare straw has the least peaks in the SQUID voltage as it passes through the coil and hence when PNRs were deposited straight in the straw, this allowed for a maximally uniform background, allowing us to pick the PNR signal as close to background free as possible in standard 'plug-and-play' SQUID operation. We report the SQUID measurements of the bare plastic straw and with the PNRs below.

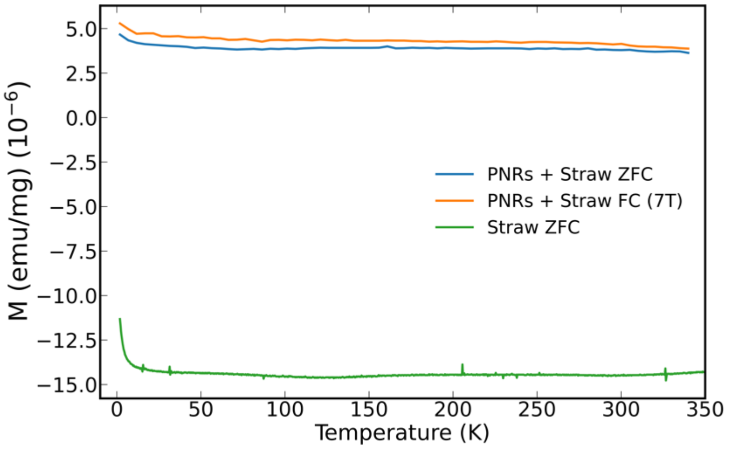

Figure S26 M *vs* T SQUID signals for PNRs in a plastic straw: A comparison of the M *vs* T SQUID signals measured on PNRs deposited straight into a plastic straw (blue/orange) *vs* the signal from an empty straw (green). Note that the diamagnetic signal measured on the straw is simply the highest peak the SQUID DC moment algorithm could find in the scanning range and is not necessarily where the sample sits.

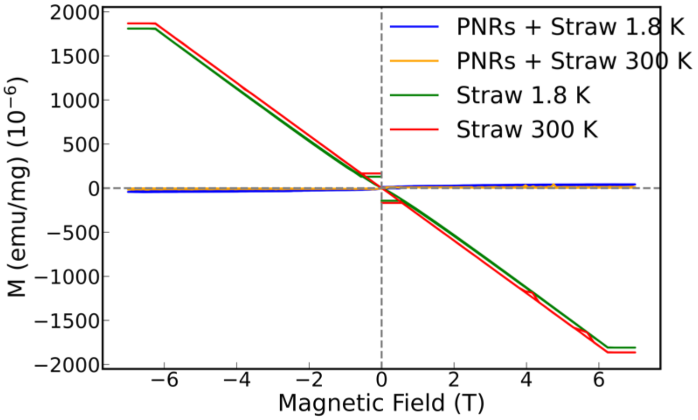

Figure S27 M *vs* H SQUID signals for filled and empty plastic straw: A comparison of the M *vs* H SQUID signals from the empty plastic straw (red and green curves) compared to the case where PNRs are deposited on the inner wall of the straw (blue and orange curves) as discussed above. The straw data demonstrates a large diamagnetic response, consistent with **Figure S26**. The horizontal lines in the red and green curve are single point outliers/artifacts in the measurement but do not influence the overall result/conclusion.

Supplementary Note 11: Raw background corrected SQUID measurements

In order to ensure that we have only contributions to the SQUID signal from the PNRs and no background signals/impurities, we have followed the background correction SQUID protocol reported in ref[46] on fresh PNR samples. We have done this by measuring the raw voltage *vs* position traces from a quartz rod and PNRs deposited on the same (blank) quartz rod. We then subtract the signals and finally fit a dipole to the traces at each field/temperature.

Zero-field cooled M(T)

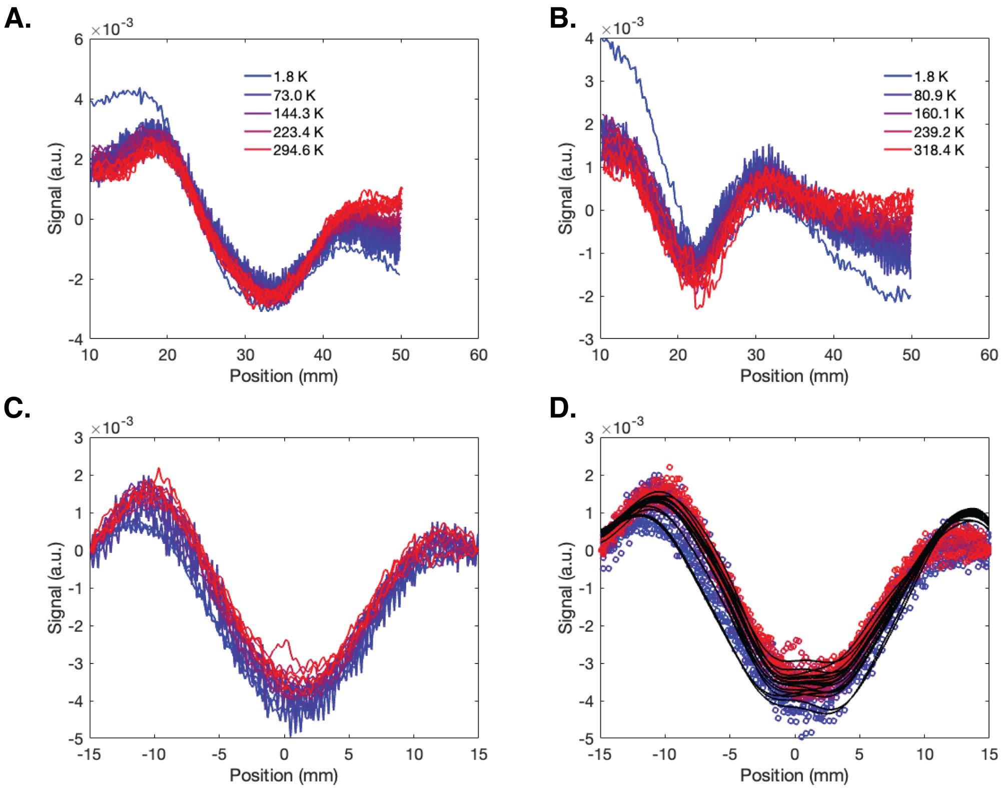

Figure S28 Extraction of ZFC SQUID signals using raw data and fitting: a. ZFC SQUID signal measured of a quartz rod with PNRs deposited on it. **b.** ZFC SQUID signal measured in the same a quartz rod before deposition of PNRs. **c.** Background subtracted traces centered at position = 0 mm for the fit. **d.** Fits to the background subtracted ZFC SQUID data yielding the M *vs* T ZFC plots shown in **Figure 1d** of the main text.

7T Field Cooled M(T)

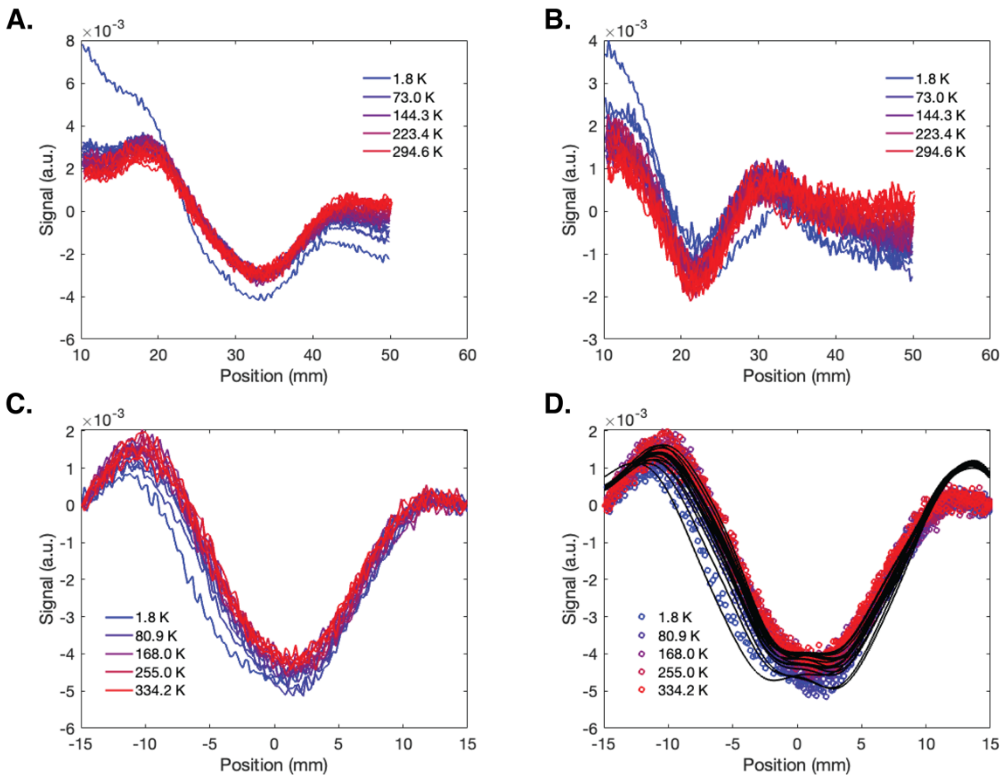

Figure S29 Extraction of FC SQUID signals using raw data and fitting: a. 7T FC SQUID signal measured of a quartz rod with PNRs deposited on it. **b.** FC SQUID signal measured on the same a quartz rod before deposition of PNRs. **c.** Background subtracted traces centred at position = 0 mm for the fit. **d.** Fits to the background subtracted FC SQUID data yielding the M *vs* T FC plots shown in **Figure 1d** of the main text.

1.8K Isothermal Magnetisation Sweeps

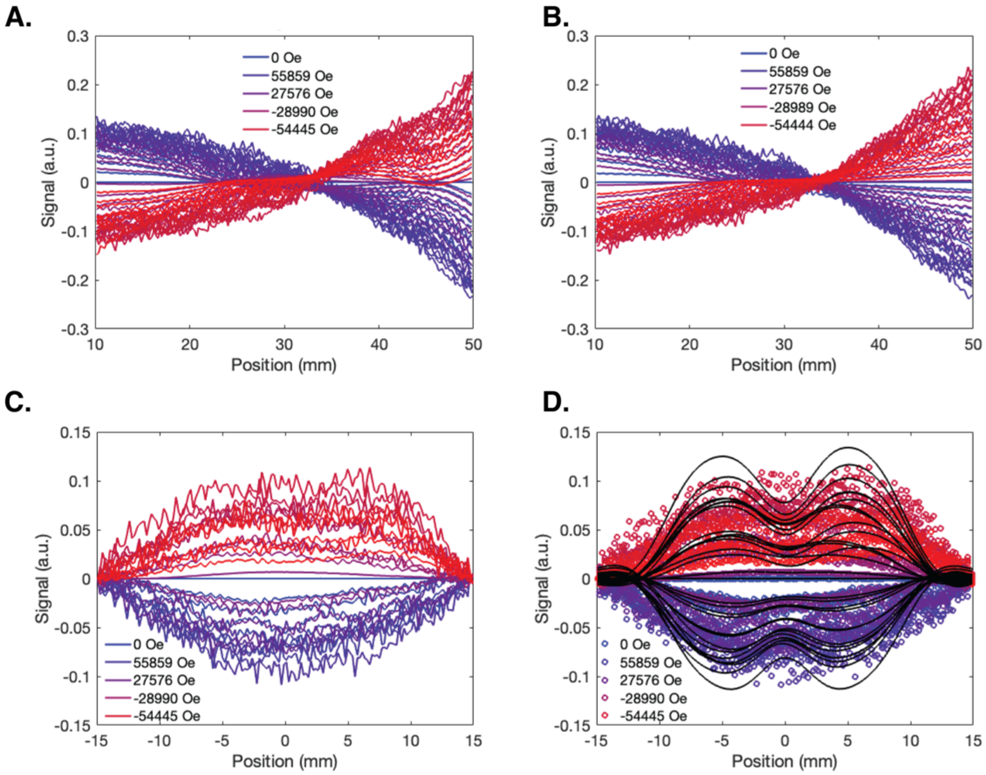

Figure S30 Extraction of M *vs* H SQUID signals using raw data and fitting at 1.8 K: a. 1.8 K M *vs* H SQUID signal measured of a quartz rod with PNRs deposited on it. **b.** 1.8K M *vs* H SQUID signal measured on the same quartz rod before deposition of PNRs. **c.** Background subtracted traces centred at position = 0 mm for the fit. **d.** Fits to the background subtracted M *vs* H SQUID data yielding the 1.8K M vs H FC plots shown in **Figure S32**.

300 K Isothermal Magnetisation Sweeps

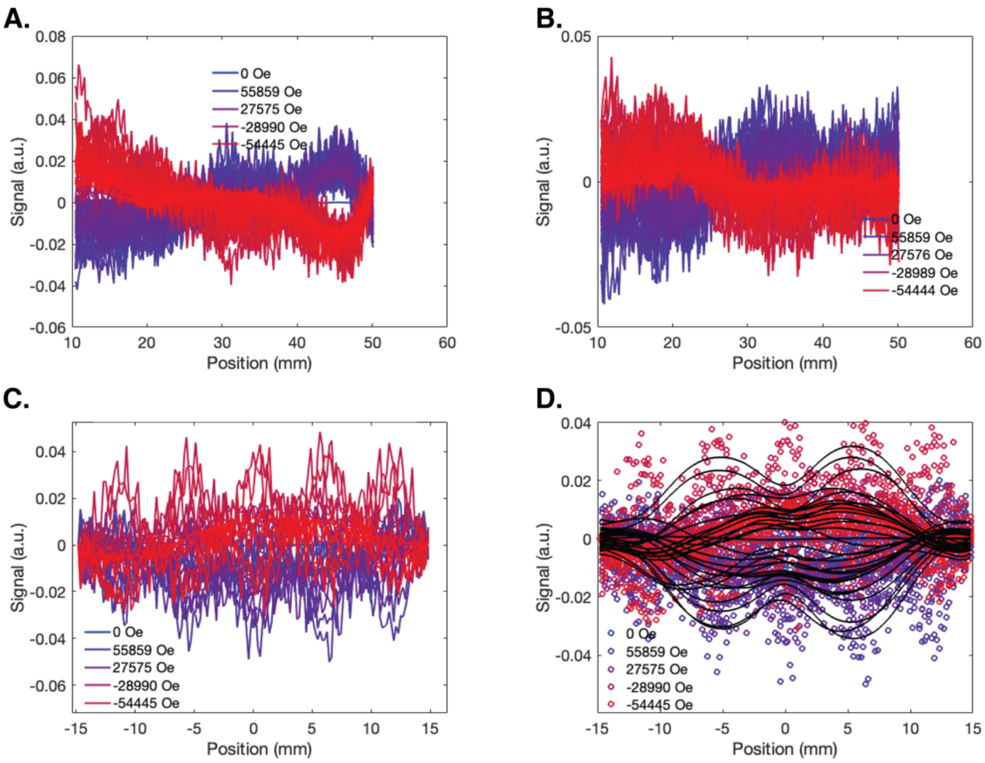

Figure S31 Extraction of M *vs* H SQUID signals using raw data and fitting at 300 K: a. 300 K M *vs* H SQUID signal measured for a quartz rod with PNRs deposited on it. **b.** 300 K M *vs* H SQUID signal measured of the same a quartz rod before deposition of PNRs. **c.** Background subtracted traces centered at position = 0 mm for the fit. **d.** Fits to the background subtracted M *vs* H SQUID data yielding the 300 K M *vs* H FC plots shown in **Figure S32**.

Comparison to non-background subtracted data

We find quantitative agreement between our ZFC and FC M *vs* T measurements regardless of whether the sample holder/substrate background is subtracted, as shown below.

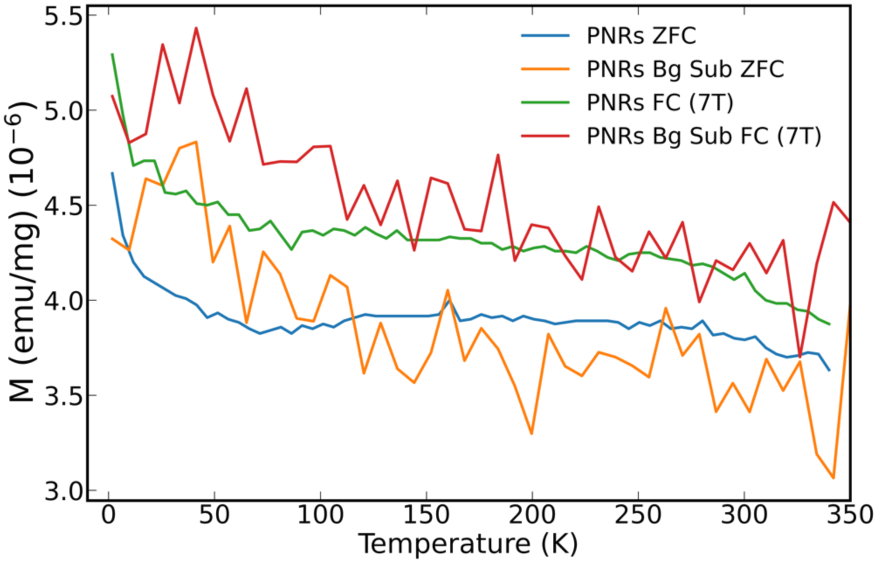

Figure S32 Quantitative comparison of background and non-background subtracted PNR signals: A comparison between the M *vs* T SQUID traces measured on the PNRs deposited straight onto the plastic straw (blue and green curves for ZFC and FC, respectively) and the post background subtracted M *vs* T SQUID traces (PNR+quartz rod – quartz rod; termed 'Bg Sub') data on the same mass of PNRs (orange and red curves for ZFC and FC, respectively). While the data is noisy following background subtraction we stress that this quantitative agreement in the SQUID data and the non-overlap of the FC and ZFC above 300K is clear evidence for the presence of a magnetic phase in these systems, which persists to temperatures above 300 K.

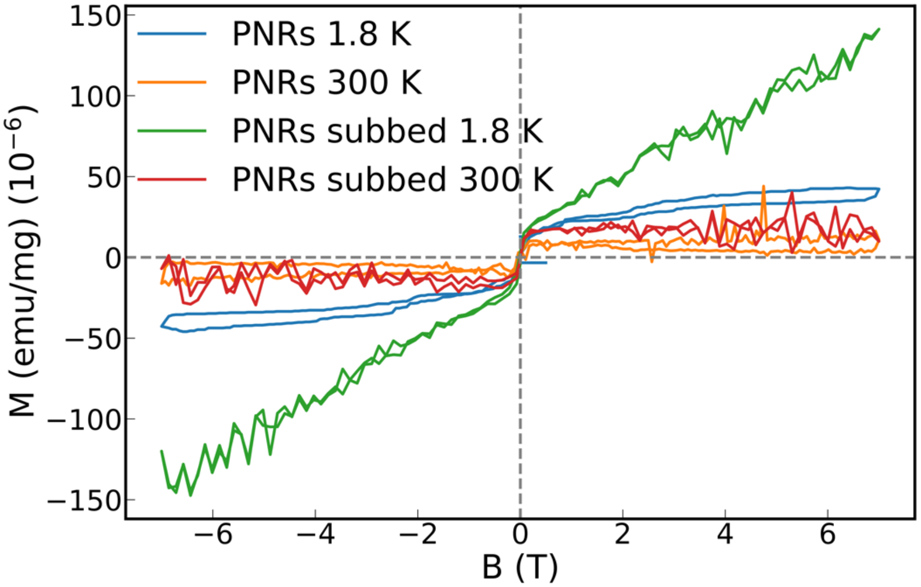

Figure S33 M *vs* H signals with and without background subtraction: A comparison of the M *vs* H SQUID traces measured on the PNRs deposited straight into the plastic straw (blue and orange curves for 1.8 and 300 K, respectively) *vs* the DC moment fits to the post background subtracted (PNR+quartz rod – quartz rod; termed 'subbed') data on the same mass of PNRs as mentioned in the main text (green and red curves for 1.8 and 300 K, respectively). In the latter case we are unable to resolve any hysteresis between the up-sweep and down-sweep traces due to the noise level of the measurement. We also cannot rule out

possible small inconsistencies of the SQUID magnet reset between subsequent runs, which could introduce measurement uncertainties of a similar magnitude to this noise level. We therefore refrain from making significant claims based on this data.

We conclude that while our background corrected M *vs* H sweeps are too noisy for us to unambiguously discern any hysteresis, the quantitative agreement in the ZFC/FC M(T) signals between the background subtracted SQUID (on quartz rods) and our measurements in the plastic straw demonstrate this data to be robust. We do all the checks needed to ensure the providence of SQUID signals, including magnet resets to ensure there is no residual flux in the SQUID and also oscillating the field to demagnetise the sample between runs. We also stress that it remains unclear if hysteresis as expected for bulk ferromagnets is applicable in the case of edge magnetism. Our claims of room temperature magnetism remain well supported by the ZFC/FC M(T) SQUID, FMR, EPR and magnetic birefringence measurements.

Supplementary Note 12: Exploration of the PNR degradation mechanism in air

FTIR Measurements

To assess the effect of air exposure on PNRs we performed FTIR measurements (using an attenuated total reflectance (ATR) FTIR mode). PNRs are deposited onto glass substrates from DMF and allowed to dry within a glove box vacuum over 30 mins. The (ATR) FTIR spectrum is then measured in the Ar glove box environment (measurements cannot be performed directly in solution due to the large solvent background). The PNR film is then removed from the glove box with the FTIR spectrum re-measured (in air) at given time intervals. Under Ar no IR modes are observed in the 600 to 4000 cm^{-1} wavenumber range we scan, beyond that of the glass substrates.

Interestingly, however after 3 hrs, 3 modes begin to grow-in centred at 1620, 1650 and 1720 cm^{-1}. Over 240 hr (10 days) the intensity of these modes increases. To the best of our knowledge, modes in this frequency range have not previously been reported for phosphorene or bulk-black phosphorous. The modes do sit in the range however for P=O bonds which are often found between 1400 – 1600 cm^{-1} (ref[47,48]). Whether these P=O (and likely other related species) are situated at the PNR edge or 'bulk' cannot be determined by FTIR but these measurements give some chemical insight into how the material degrades. We note that these modes only grow in with significant intensity after 3 hrs which is longer than the ~20 mins for which we expose PNRs to in **Figure S23** before performing SQUID measurements. This suggests that even some limited chemical changes from the pristine PNRs can destroy the magnetic state. Although we also note that the measurements in **Figure S23** involved bubbling air through a solution of PNRs which likely accelerates the degradation process. We comment that based on the FTIR measurements any small exposure to air, e.g., during transfer from the glovebox into the SQUID (sub-5 mins) likely has limited effect on the PNR structure/properties.

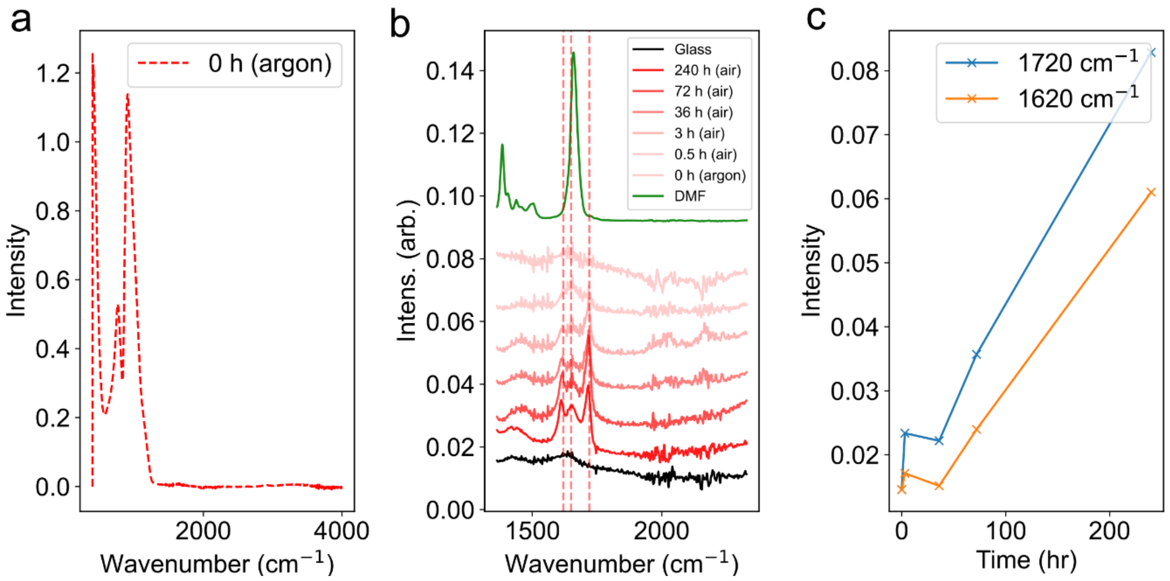

Figure S34 FTIR characterisation of PNRs: a. FTIR spectrum of PNRs in Ar. All the peaks observed arise from the glass substrate**. b.** FTIR spectrum between 1370 and 2345 cm^{-1} of PNRs at select times after exposure to air (red). The FTIR spectrum of the glass substrate and DMF solvent PNRs are dispersed in is marked in green and these background modes are distinct from the three modes at 1620, 1650 and 1720 cm^{-1} which arise from the PNRs. **c.** Evolution of intensity of 1620 and 1720 cm^{-1} modes of PNRs over time.

XPS Measurements

In addition to the FTIR measurements performed above we have also performed XPS measurements to understand what happens to PNRs (chemically and structurally) when exposed to oxygen. PNRs were drop cast from NMP solution onto silicon wafer substrates and dried under vacuum in a glovebox antechamber. For 'pristine' samples the PNRs are kept in an Ar environment throughout the preparation, storage and measurement. For air exposed samples to compare to SQUID measurements, an aliquot of NMP solution (~1 mL) was taken from the glovebox, exposed to the atmosphere, shaken, and left for ~20 min before dropcasting on a silicon wafer and drying in a glovebox antechamber, subsequently moving the sample into the glovebox. In the glovebox, both samples were adhered to an air-free XPS holder assembly with carbon tabs. Our XPS spectra (shown in **Figure S35**) show this technique is more sensitive to changes in the PNR nature on exposure to air than FTIR. However, we note that XPS is much more surface sensitive than FTIR and while the PNRs on the surface may be degraded, the interior of the film can be 'protected' from the air (with the majority of our magnetic signals in SQUID and EPR likely occurring from the bulk film). Consequently, care must be taken in comparing between techniques and in determining degradation timescales.

The P2p signal shown in **Figure S36a** is indicative of heavy oxidation. On exposure to air the pair of peaks corresponding to the oxide (>132 eV) become significantly more intense than the basal plane "neutral" phosphorus pair (~130 eV). The magnitude of the increase does imply basal plane oxidation, not edge-only which would be limited to a proportion fraction increase, equal to the fraction of edge sites. The neutral peaks also have a statistically significant +0.6 eV shift upon oxidation (errors $\leq \pm 0.2$ eV) implying even phosphorus atoms lacking P-O bonding motifs are p-doped by the excess oxidation.

The O1s signal (**Figure S36c**) shows a reasonable difference on aerial oxidation of the sample. However, a caveat that must be noted here is that the more dominant peak at 532.7 eV of SiO_2 at the substrate surface may influence the fitting. The initial peak at 510.8 eV in the air free sample is in a spectral region that is a little surprising and distinct from the usual phosphorus complexes that could residually be present (i.e., PO_4^{3-}, PO_3^{-}, P_2O_5) with highly oxidised P^{5+} centres. Upon oxidation, this peak shifts to 531.2 eV, typical of P^{5+} atoms, implying oxidation forms highly oxidised phosphate ions, or something similar.

Lithium is the only metal directly involved in the synthesis of PNRs, and a possible residual component coordinated to the solvent. The signal is very weak in the air free samples but a signal that could be Li emerges more clearly in the oxidised PNRs. Lithium is tricky to observe by XPS as it has an inherently low signal and is also very hard to assign chemistry too given the spectral range the peaks of Li are in. This is not withstanding the low experimental stoichiometry of Li in the precursor intercalation compound. Our results might suggest there is a plurality of local lithium environments in the pristine PNRs which smears out the signal *versus* an expected monocomponent LiOH in oxidised PNRs. Given the raw counts of the oxidised sample are low compared to the air-free sample, it is unlikely that the signal difference arises from the amount of material cast. However, the role of residual Li in the properties of PNRs remains to be investigated, indeed the 'peak' at 55 eV is not dissimilar to the level of noise in the rising background and hence we reserve on commenting on any Li that may be present in the samples.

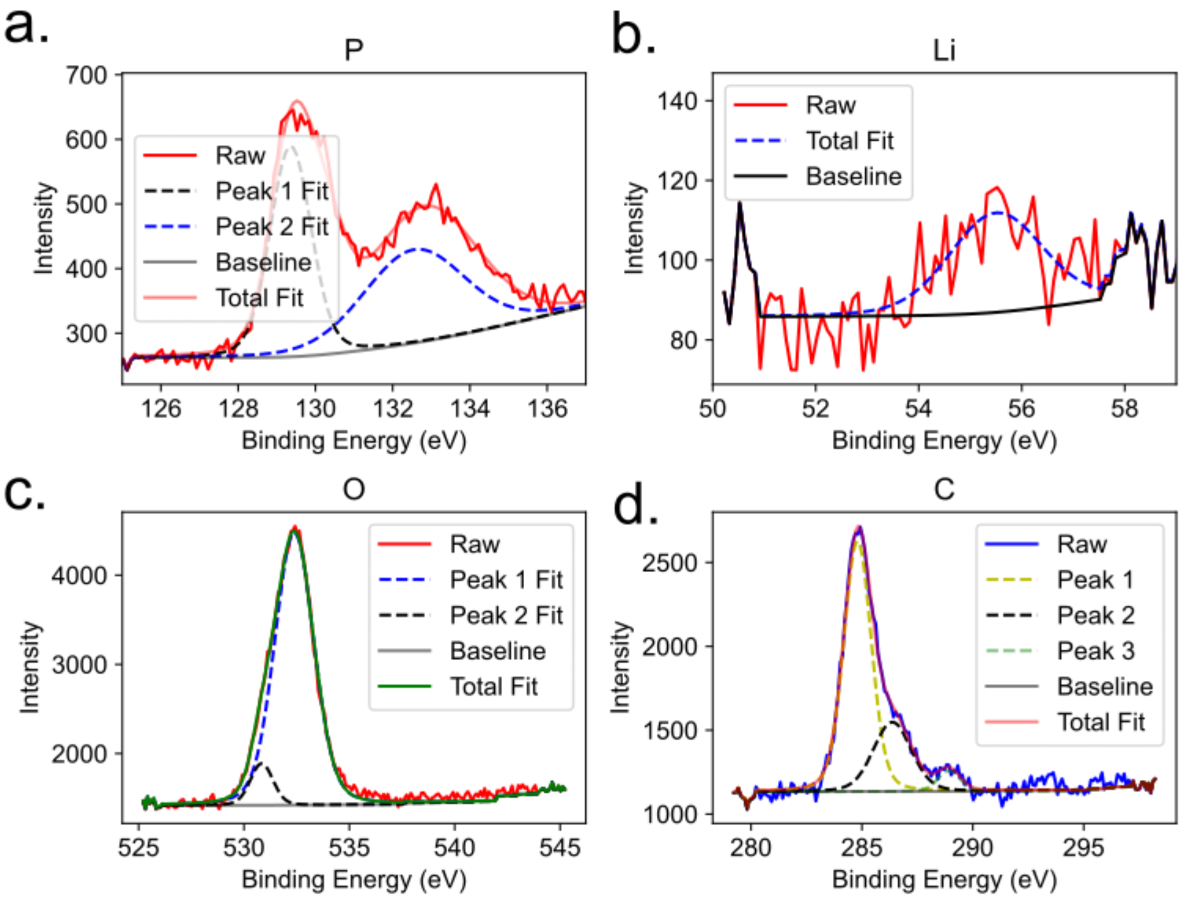

Figure S35 XPS characterisation of PNRs before air exposure: a-d. XPS spectra of P (a), Li (b), O (c) and C (d) in pristine PNR samples. Raw data and fits are marked appropriately. Samples calibrated to C1s advantageous carbon (284.8 eV) and fitted with a Shirley background. P2p data was fitted with fixed spin orbit splitting (0.86 eV).

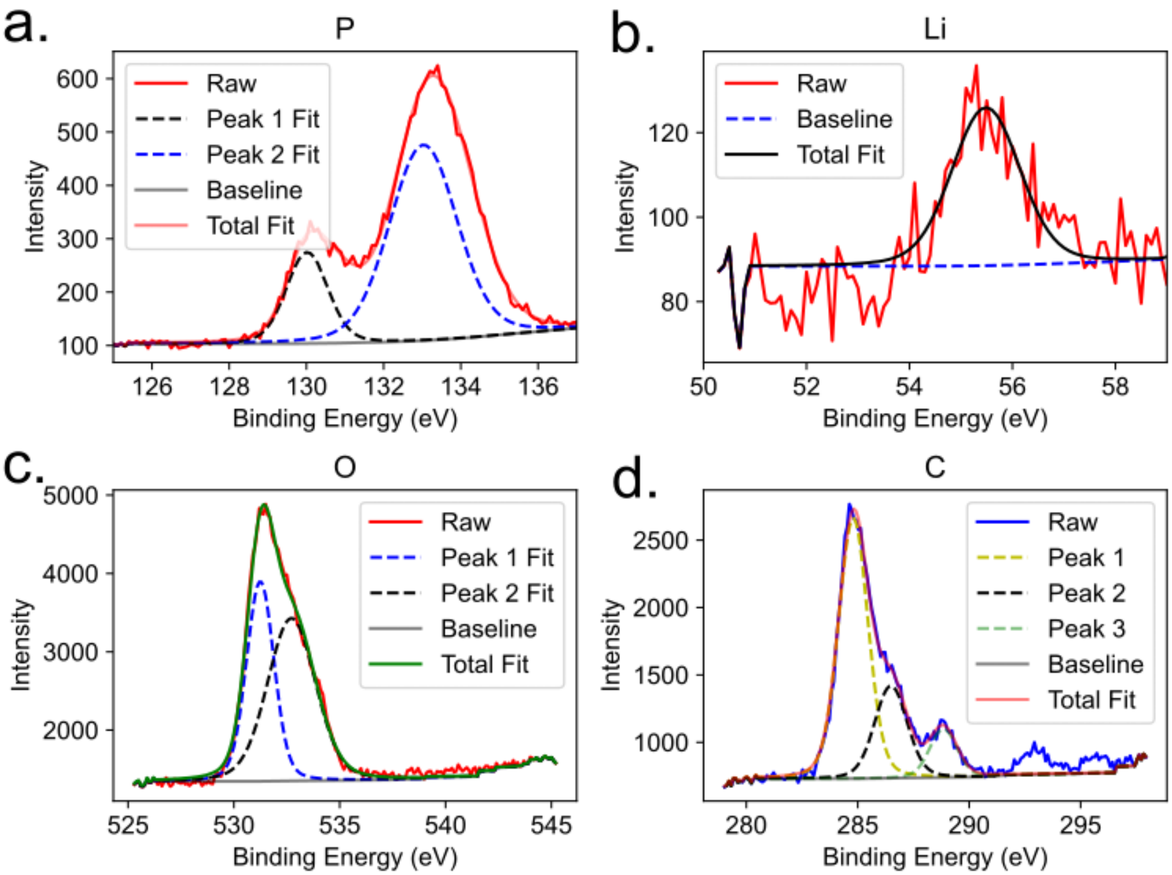

Figure S36 XPS characterisation of PNRs after air exposure: a-d. XPS spectra of P (a), Li (b), O (c) and C (d) in PNR samples after air exposure. Raw data and fits are marked appropriately. Samples calibrated to C1s advantageous carbon (284.8 eV) and fitted with a Shirley background. P2p data was fitted with fixed spin orbit splitting (0.86 eV).

Raman Measurements

Finally, we performed Raman spectroscopy on a sample as it was exposed to air focussing on monitoring the intensity of the A^1_g bulk and B^1_{3g} edge phonon modes. Interestingly, while the former's intensity drops by only ~15% over 2 hrs of measurement in air, the latter drops 100% in intensity within 75 mins. These results support the idea that the edges are the first point of reaction on exposure to air and the main structural changes are occurring here.

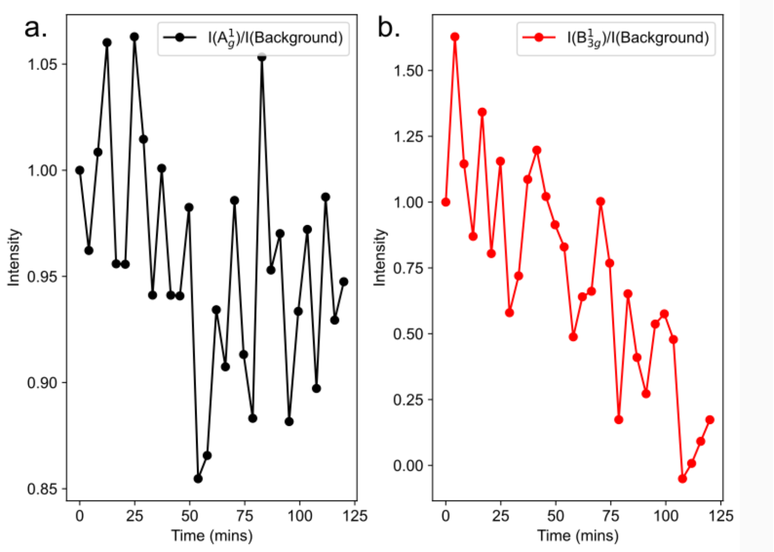

Figure S37 Raman characterisation of PNRs after air exposure: a. Normalised intensity (normalised to initial value) of bulk PNR A^1_g mode/background (baseline counts) over time. **b.** Normalised intensity of B^1_{3g} edge mode/background (baseline counts) over time. Error bars are not shown to prevent obscuring of plot but are approximately 15% of values.

Supplementary Note 13: Elemental Analysis of the PNR solution (ICP-OES)

With regards to impurities in batches of PNRs themselves, which could contribute to the SQUID data and EPR signals after background contributions from substrates, etc have been rules out, we have performed ICP-OES measurements to characterise the amount of magnetic Fe, Cr, Mn, Ni species that could be present. PNR samples ~2 mg/ml in concentration were digested in 2% nitric acid with 0, 0.5 1, 10, 100, 1000 ng/ml concentration standards used to calibrate the instrument. The elemental sensitivity of the system was ~1 ppb (lowest measurement reading). The elemental concentrations found are detailed in **Table 2** and **Table 3** and are sufficiently low such that their contribution to the SQUID signal can be ignored.

With regards to the EPR data, generally, an X-band EPR setup can be sensitive to ~ 1×10^{10} to 1×10^{11} spins per mT linewidth (or 1×10^{9} to 1×10^{10} spins per Gauss linewidth) depending on the exact setup. The EPR data presented in the main text was from a sample made from 400 μl of a 1 mg/ml PNR batch in DMF solution. With the maximum impurity from any batch being Fe, at 12 ppb, this would mean a possible ~5×10^{10} Fe atoms in our sample based on:

$$\frac{0.4 \times 10^{-3} \times 6.022 \times 10^{23}}{56} \times 12 \times 10^{-9} = 5.1 \times 10^{10}$$

However, since the $g\approx2$ signal we observe is 12-20 mT wide, the detection limit for such a linewidth would be 1×10^{11} to 1×10^{12} spins. In other words, the Fe concentration is too low by one to two orders of magnitude to be contributing to our lowest intensity (narrowest) $g\approx2$ signal.

Below are tables detailing an elemental analysis of the PNR batches made from a variety of sources and in a variety of solvents that we use in this study.

	Fe (ppb)	Ni (ppb)	Co (ppb)	Cr (ppb)	Mn (ppb)
2D semiconductors PNRs in NMP	10.1	0.2	<0.1	<0.1	<0.1
Smart Materials PNRs in NMP	8	0.2	<0.1	<0.1	<0.1
Smart Materials PNRs in DMF	5	0.2	<0.1	<0.1	<0.1
DMF solvent	6	0.4	<0.1	<0.1	<0.1
NMP solvent	7	0.2	<0.1	<0.1	<0.1
LiP_8	6.4	0.2	<0.1	<0.1	<0.1
2D semiconductors Black Phosphorous	1.75	0.14	<0.1	<0.1	<0.1

Table S2 Results of the Inductively Coupled Plasma Optical Emission Spectroscopy (ICP-OES) on the PNR solution: ICP-OES of PNRs made from two different black phosphorous precursors (2D semiconductors and Smart Materials), DMF and NMP solvents, LiP_8 intermediates and black phosphorous (2D semiconductors). The instrument resolution is 1 ppb, hence while we report readings between 0.1 and 1 ppb for Ni these likely correspond to negligible concentration values. No Co, Cr and Mn are detected

within our resolution limit either. Based on the magnetisation per unit mass of Fe (the highest trace magnetic impurity) 217.6 emu/g, 9.2×10^{-8} g of Fe would be required to produce our observed M *vs* T SQUID signals. This corresponds to 97.8 ppb. The amount of Fe we detect is well below this amount. This suggests our SQUID results cannot be explained by any magnetic impurities.

Below is a table detailing an elemental analysis of a variety of different PNR batches to ensure reproducibility in quality across the different batches used in our study.

	Fe (ppb)	Ni (ppb)	Co (ppb)	Cr (ppb)	Mn (ppb)
Smart Materials PNRs in DMF Batch 1	12.2	0.2	<0.1	<0.1	<0.1
Smart Materials PNRs in NMP Batch 1	10	0.2	<0.1	<0.1	<0.1
Smart Materials PNRs in DMF Batch 2	9.1	0.1	<0.1	<0.1	<0.1
Smart Materials PNRS in NMP Batch 2	11.2	0.1	<0.1	<0.1	<0.1
Smart Materials PNRs in DMF Batch 3	10.3	0.2	<0.1	<0.1	<0.1
Smart Materials PNRS in NMP Batch 3	10.1	0.2	<0.1	<0.1	<0.1
Smart Materials PNRs in DMF Batch 4	8	0.1	<0.1	<0.1	<0.1
Smart Materials PNRS in NMP Batch 4	7	0.1	<0.1	<0.1	<0.1

Table S3 ICP-OES of PNRs (in DMF and NMP solvents) made from the main black phosphorous precursor used in this work (Smart Materials): The concentration of trace magnetic metals is sufficiently low that they can be discounted for giving rise to the SQUID or EPR signals we observe. We note that the ICP-OES sensitivity is around 1 ppb hence for Ni, Co, Cr and Mn we take the concentration to be below the detection level.

Supplementary Note 14: Low Temperature Phase Transition (optical and magnetic data)

SQUID Magnetisation

As well as in the EPR data there is also a discontinuity suggestive of a phase transition in the SQUID magnetisation data (ZFC/FC; **Figure 1d** of the main text) where we observe an abrupt change in the magnetisation on cooling below 55-70 K. This can be revealed further by plotting M *vs* 1/T as shown below.

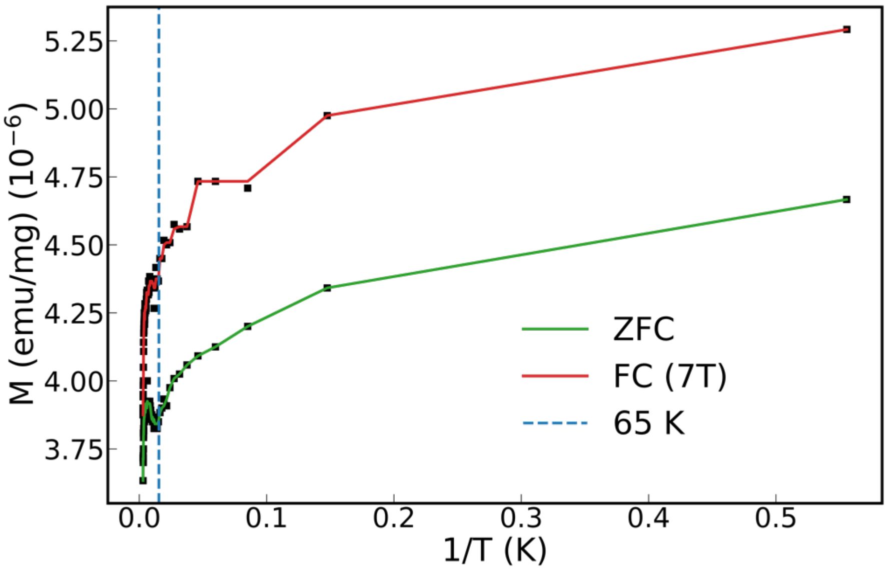

Figure S38 Magnetisation *vs* 1/T for data shown in Figure 1d of the main text for ZFC/FC: The dashed line indicates a clear discontinuity in the behaviour around 55-70 K. This is present in both the ZFC and FC traces but clearest in the former.

Temperature Dependent Absorption

As shown in **Figure S39** the absorption edge of PNRs red shifts ~150 meV on cooling from 300 to 50 K, with some discontinuity observed around 55 to 65 K. We note that the broad tails in the absorption spectra make it challenging to define a band-edge, we hence take the band-edge as when the normalised absorption drops to 0.15 as this is where the gradient of the absorption spectrum is largest. We apply the same analysis as discussed in **Supplementary Note 20** to model the evolution of the absorption edge *vs* temperature fitting the data to an equation of the form $E = [a'\theta/(e^{\frac{\theta}{T}} - 1)] + mT + c$ (**Equation 7**). Where mT is a linear term capturing the evolution of the band gap due to thermal expansion, a is a constant, c is a parameter related to the band gap at T = 0 K (but can be removed by normalising the data) and θ is the effective frequency for the dominant electron-phonon coupling mode[49]. We find a' = +0.10 meV/K, θ = 350 K and m =–0.35 meV/K. This θ value is similar to the B_{1g} edge phonon mode frequency (as we also find when fitting the PL emission energies as a function of temperature). Further discussion of the importance of these values is provided in **Supplementary Note 20**.

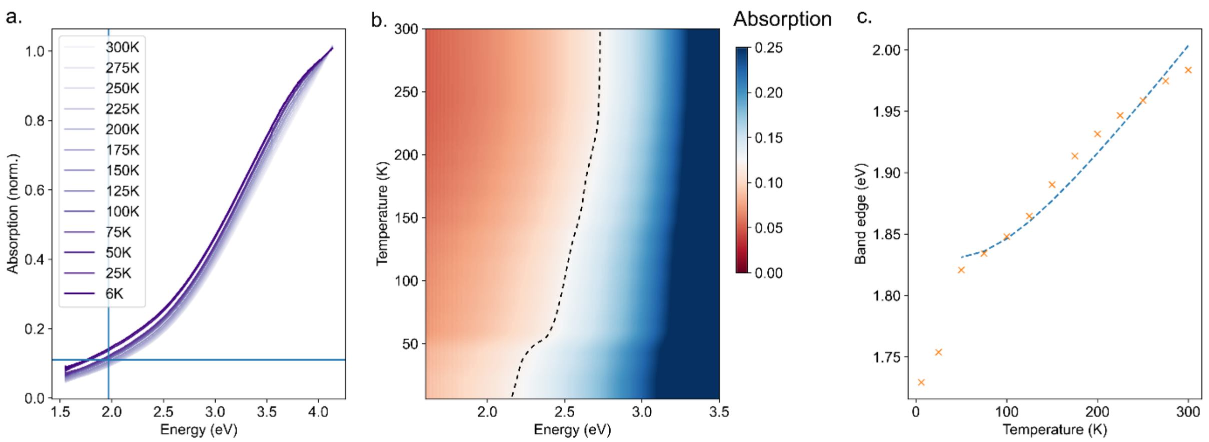

Figure S39 Absorption spectrum of PNRs as a function of temperature: a. Absorption spectra of PNRs as a function of temperature. The horizontal blue line indicates the intensity at which the 'band edge' is extracted from. **b.** Map of absorption spectra as a function of temperature. The map has been saturated to highlight the low temperature discontinuity. The contour is a trace of the intensity at an absorption value of ~0.18. **c.** Position of the 'band edge' as extracted from UV-Vis measurements as a function of temperature (orange crosses). Blue dashed line shows fit to model detailed in **Equation 7**.

<u>Temperature dependent Raman spectroscopy</u>

To understand the influence of thermal disorder on the vibrational properties, which could also contribute to line broadening, we perform temperature dependent Raman measurements. We fit the Raman spectra as a function of temperature to a model of 3 Lorentzian functions that capture the main A_g^2, B_{2g} and A_g^1 symmetry modes in the spectra. Between 70 and 300 K there is limited change in the linewidth (<1 cm^{-1}), position (<2 cm^{-1}) or amplitude of all modes. This suggests again that thermal disorder does not play a significant role on the vibrational properties of PNRs. In other words, at room temperature, the vibrational linewidths are already lifetime limited. Below 55/60 K (albeit with limited resolution) there is an abrupt change in the Raman peak positions, width and amplitude. This is similar to the range where we observe phase transitions in the UV-Vis spectra, PL spectra, EPR and SQUID magnetometry experiments. The fact that the 'phase transition' appears to be present in bulk modes of the PNRs suggests that it is not only the edges which are being modified at low temperatures, but the bulk PNR lattice itself. The behaviour, we note, is also reversible on warming. However, we refrain from commenting on the exact properties of this low temperature phase as we do not know if the broadening of modes, for example, arises from changes in structural disorder or vibrational lifetime changes (the fact that the emission lifetime does not change with temperature suggests the former). We note this avenue of inquiry while interesting is not necessary to support the claims we make in the paper, which do not depend on a detailed knowledge of the 50-60 K phase transition or broadening mechanism of the vibrational linewidths of PNRs, as we focus on room temperature properties of the PNRs.

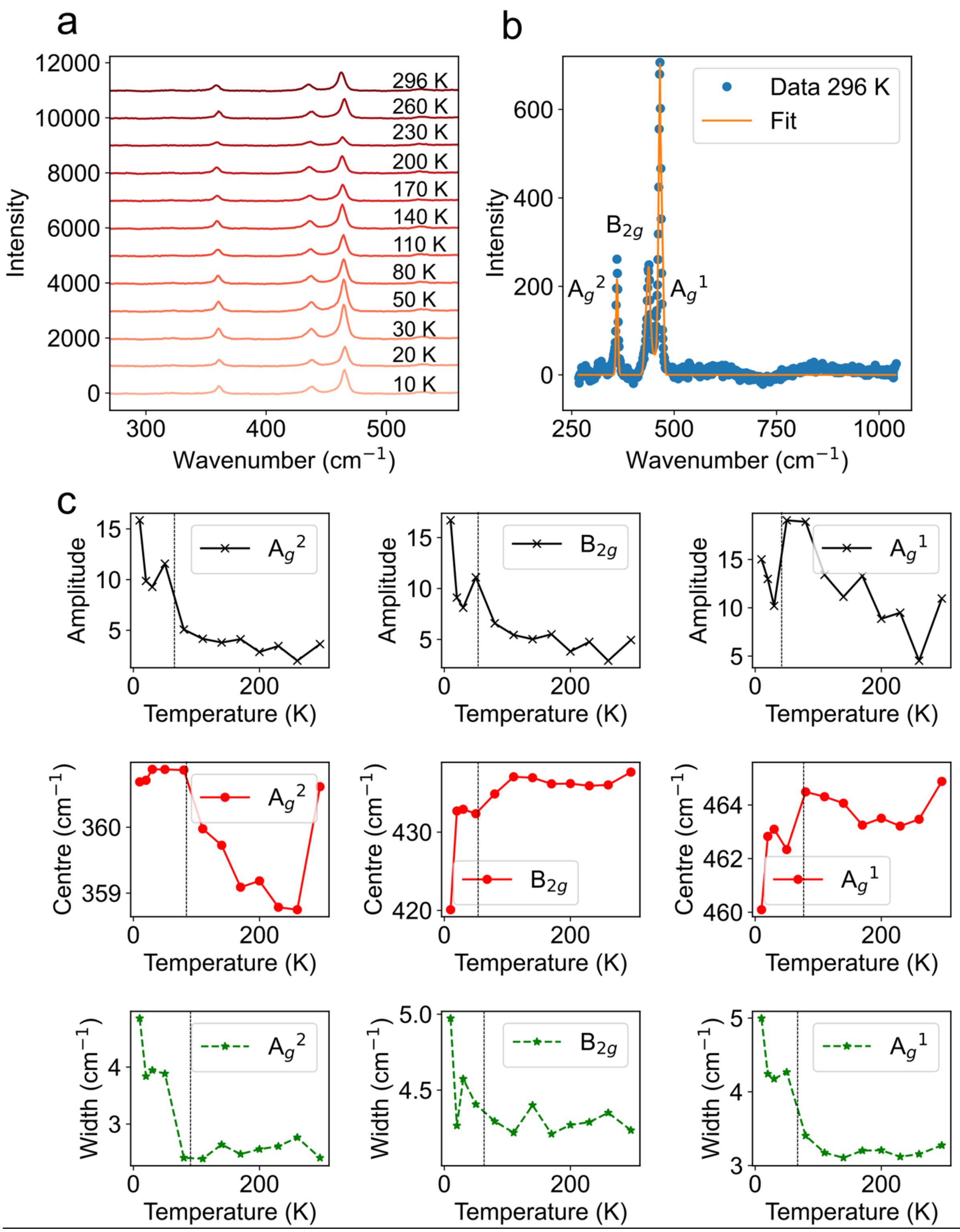

Figure S40 Raman spectrum of PNRs as a function of temperature: a. (Unpolarised) Raman spectra of PNRs between 10 and 296 K in the 260 to 550 cm^{-1} wavenumber regime. **b.** (Unpolarised) Raman spectrum of PNRs at 296 K with the orange line showing a fit of 3 Lorentzians to the spectrum consisting of an A_g^2 at ~370 cm^{-1}, a B_{2g} mode at 450 cm^{-1} and A_g^1 mode at 490 cm^{-1}. The background of the glass substrate is subtracted before fitting. **c.** Amplitude, centre frequency and width of A_g^2, B_{2g} and A_g^1 modes as a function

of temperature. The dashed line indicates where qualitatively the mode properties (amplitude, centre frequency or width) show a discontinuity or change non-monotonically. As this temperature is not the same for every mode property we give a range of temperatures for the ascribed phase transition.

Temperature dependent PL spectroscopy

A detailed discussion of the temperature dependent PL is provided in **Supplementary Note 20**. We note here that the same phase transition at 50-65 K see in other optical and magnetic measurements is seen in the temperature dependent PL (see **Figure S66**).

Supplementary Note 15: Additional EPR magnetometry measurements

Narrow field-range (around the $g \approx 2$ signal) cwEPR measurements were carried out on Setup 1 (see the experimental methods in the main text) as this allowed for higher sensitivity, using a high-quality factor (Q-factor) Bruker ER 4122-SHQE resonator. It also allowed for field monitoring using a Bruker ER 035 M NMR Gaussmeter along with calibration using a standard N@C60 sample with a well-known g-factor of 2.00204 [50].

We find that at room temperature there exists a broad $g \approx 2$ signal, which confirms the presence of unpaired electrons in the inner wall PNR thin film sample. We carry out an orientation study by rotating around the long axis of the EPR tube. In a homogenous sample, there should not be any orientation dependence to the EPR signal. However, it was clear by eye that the inner wall film was highly inhomogeneous with different thicknesses (see also **Supplementary Note 9**). This is confirmed via EPR where we see a strong dependency to both the g-factor and peak to peak linewidth (lwpp) as a function of rotation. We rotate the sample tube though 360° in steps of 10° and find the maximum g-factor of ≈ 2.20/2.21 is reached at angles $\theta \approx$ 40°, 130° (repeating at 220°, 310°) and a minimum g-factor of 2.03 at 80° (repeating at 260°). The maximum determined g-factor of 2.20-2.21 is in line with the calculated out-of-plane g-factor for bilayer/2 layer (2L) back phosphorous[51]. While the minimum g-factor determined to be 2.03 is in line with the calculated in-plane g-factor (the in-plane g-factor is not considered to have a layer dependence). Finally, examining the angle dependence of the g-factor we clearly see that most data points are around the 2.1-2.15 value, and 2.14 is the calculated out-of-plane g-factor for monolayer black phosphorus. Hence, the $g \approx 2$ EPR signal must occur due to predominantly mono- and bi-layers of PNRs and not a greater number of layers. In addition we suggest that the smooth temperature dependence of the g-factor from 300 to 65 K signals a slow spin-canting in the out-of-plane direction due to antiferromagnetic (AF) correlations which lowers the g-factor from the fully out-of-plane polarised value of 2.14 towards the in-plane value of 2.00[52].

θ (°)	*g*-factor	Lorentzian lwpp (mT)
0	2.1239	13.5
10	2.1451	17.9
20	2.1603	19.0
30	2.2070	19.4
40	2.2127	17.4
50	2.1267	17.7
60	2.0670	14.7
70	2.0403	13.6
80	2.0291	14.0
90	2.0411	13.5
100	2.0884	12.1
110	2.1424	13.9
120	2.1901	14.9
130	2.1998	13.0
140	2.1747	13.4
150	2.1361	12.5
160	2.0983	12.6
170	2.0962	11.8
180	2.1182	12.5

190	2.1382	17.6
200	2.1550	19.5
210	2.1910	17.5
220	2.2110	13.5
230	2.1276	18.6
240	2.0702	14.3
250	2.0436	11.8
260	2.0350	13.0
270	2.0426	12.9
280	2.0838	11.9
290	2.1412	14.2
300	2.1914	13.3
310	2.2018	14.2
320	2.1782	14.6
330	2.1382	13.0
340	2.0996	12.5
350	2.1014	12.0
360	2.1260	13.5

Table S4 Extracted *g*-factor and linewidth of the *g*≈2 EPR signal as a function of rotation angle. The data is taken at room temperature.

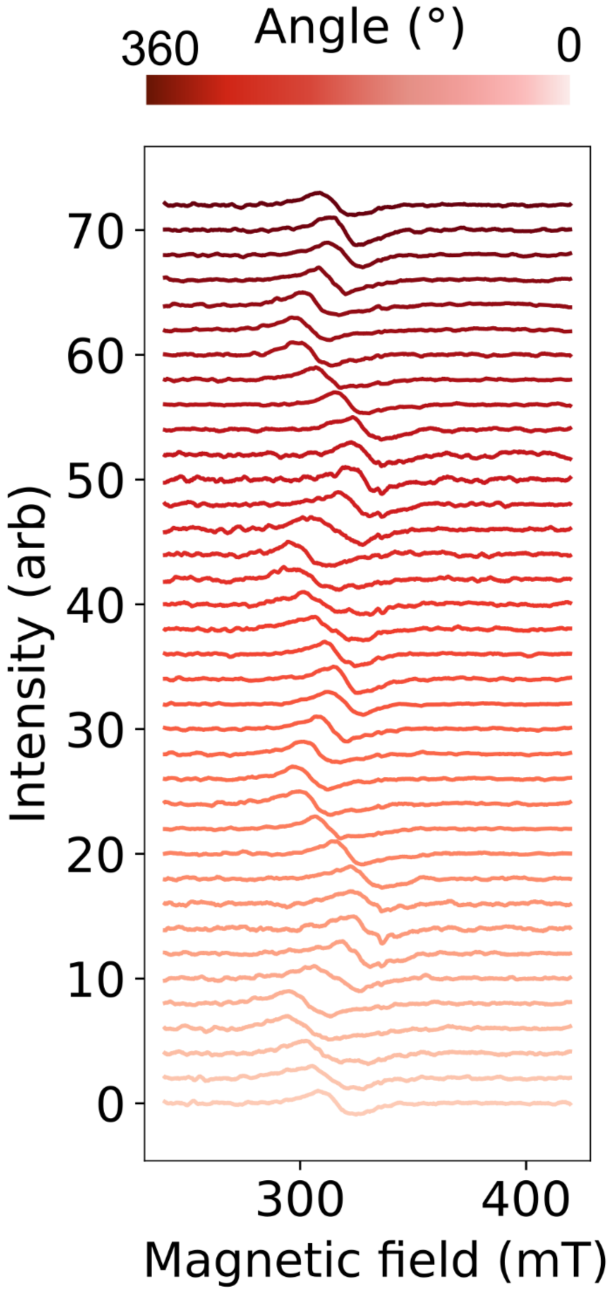

Figure S41 *g*≈2 EPR signal as a function of rotation angle at room temperature. The FMR signal/slope has been removed.

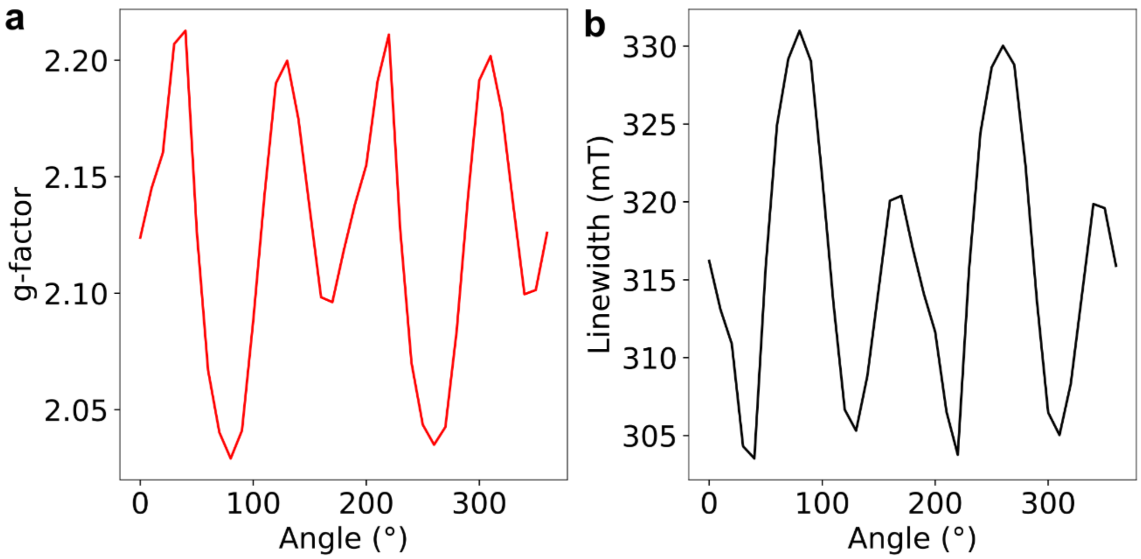

Figure S42 *g*-factor of the *g*≈2 EPR signal and resonance field as a function of tube orientation: a. Extracted *g*-factor of the *g*≈2 EPR signal as a function of rotation angle at room. **b.** Resonance field as a function of angle. Both are at room temperature.

Next, we investigate the cwEPR spectrum as a function of lowering temperature, from 296 K to 15 K by sitting at an angle of θ ≈ 100° (see main text **Figure 2c**). At θ ≈ 100° the *g*-factor is 2.0908, not too far off the out-of-plane monolayer value but far-off from the in-plane value. Most crucially however, at this angle the linewidth is the narrowest, which gives the best opportunity to see changes in the cwEPR spectra as a function of decreasing temperature.

We see that at θ ≈ 100°, lowering the temperature from 296 K down to 195 K, leads to an increasing *g*-factor from 2.0908 to 2.1124 and an increasing lwpp (line-width peak-to-peak) from 12.3 mT to 13.9 mT. From 195 K to 75 K the *g*-factor sees the reverse behaviour where it decreases from 2.1124 to 2.0567, while the linewidth remains much broader than at 296 K. The *g*-factor increases from 75 K to 65 K before vanishing around 55 K. The disappearance of the signal and the increased linewidth either side of this 50-60 K range is in line with observations of a phase transition, around this temperature range, as seen in the SQUID measurements. At 35 K the signal reappears with a *g*-factor of 2.0464 and continues to increase until 15 K reaching 2.0563. The sharper signal which does not change with orientation but is more prominent at lower temperature is a background signal from the resonator cavity (see discussion below).

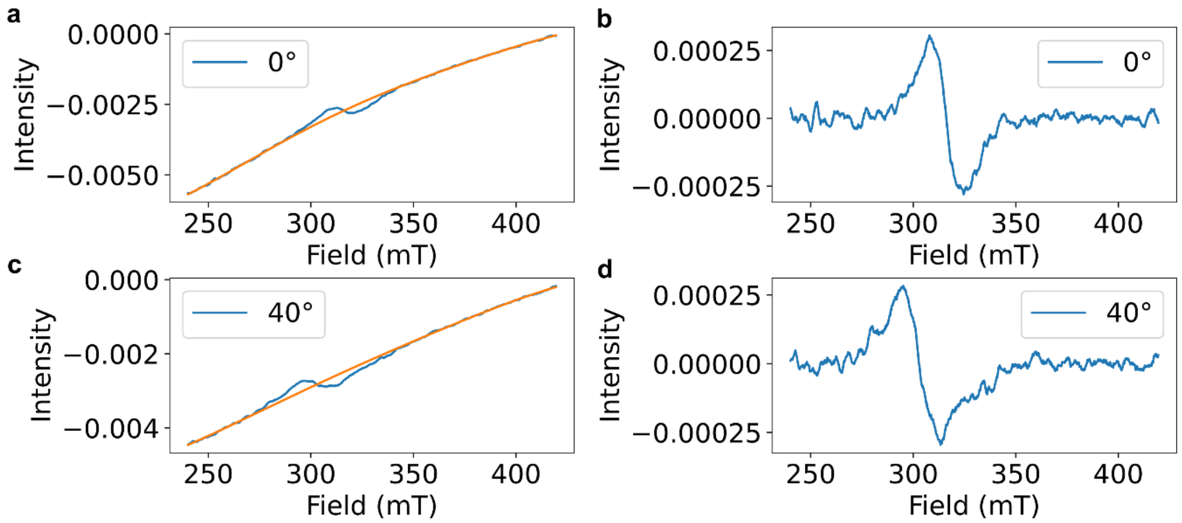

Figure S43 Background subtraction of EPR data: a-b. Raw signal, polynomial fit (a) and corrected signal (b) at 0°. **c-d.** Raw signal, polynomial fit (c) and corrected signal (d) at 40°.

In all the raw narrow scan cwEPR spectra there is a slope which corresponds to the FMR signal (which also has a strong orientation dependency). In order to determine the *g*-factor, lwpp and to clearly see the trend in the EPR signal we removed the FMR signal's slope using a suitable polynomial fit. An example of the raw signal, polynomial fit and corrected signal is given in **Figure S43** for angles denoted 0° and 40°. Similar processing was carried out on all narrow scan cwEPR spectra (rotation study and temperature series) to only show the $g \approx 2$ EPR signal.

Next, we move to Setup 2 (see the experimental methods in the main text), which allows for broader magnetic field scans going from 6 mT to 1.45 T. This board magnetic field range means field calibration is not possible however we do not expect any field offset to be more than 0.4 mT. The wide scan reveals additional signals, apart from the $g \approx 2$ EPR signal, which are much more intense and even broader. We attribute these to ferromagnetic resonance (FMR) signals, which most likely result from domains where there is strong interactions between the PNR layers. The FMR signals exhibit a strong orientation dependency, however, there seems to be more than one domain contributing, with some signals significantly changing with sample tube rotation and some not changing considerably. While the analysis of the FMR signals is increased in complexity by the inner wall film and its inhomogeneity, we attempt to show the possible behaviour of different domains by fitting the data at each angle with four derivative signals. Three corresponding to the FMR signals (two purely Lorentzian and one Voigtian component) and one purely Lorentzian derivative for the $g \approx 2$ EPR signal.

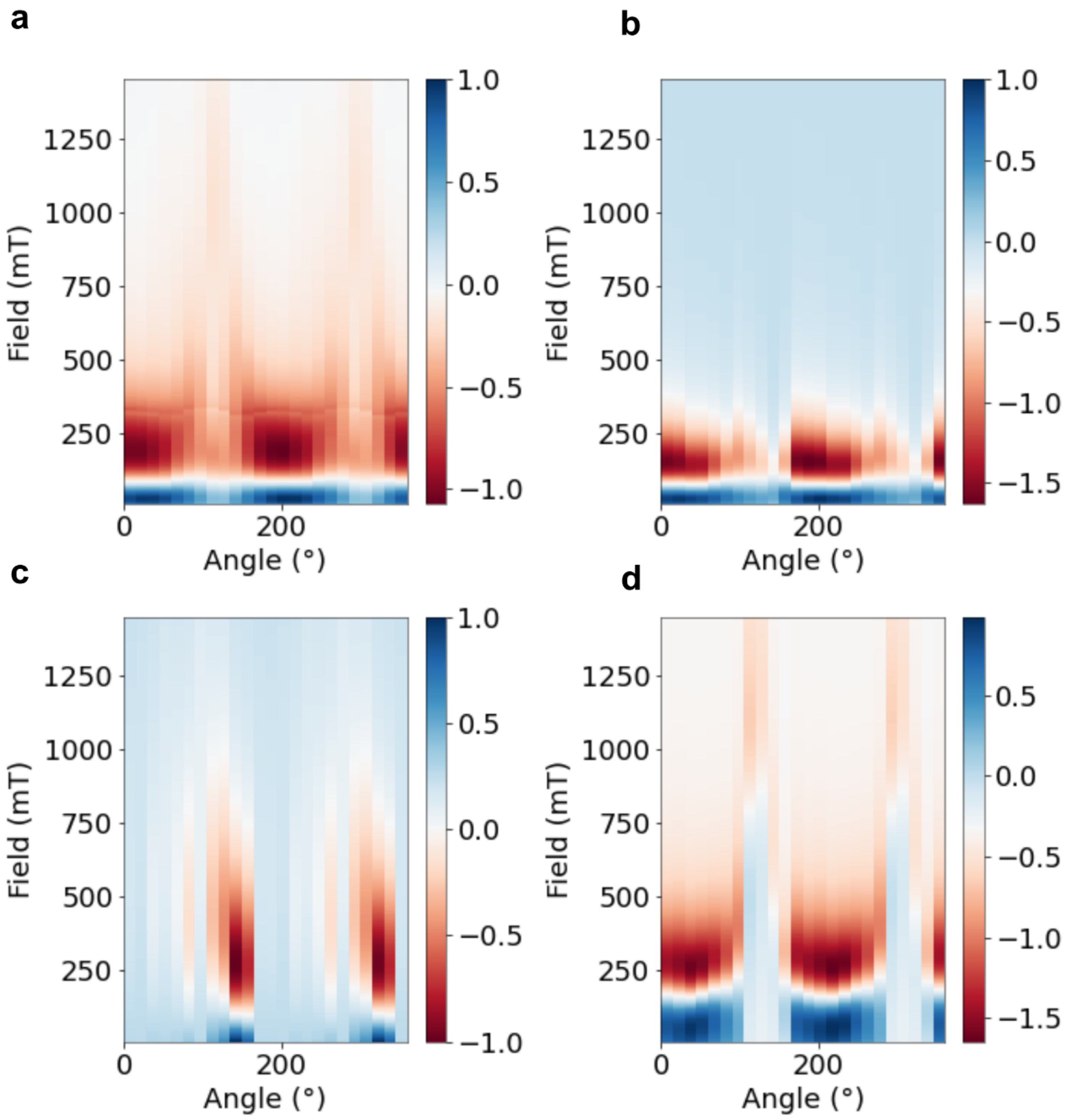

Figure S44 Fitting of EPR/FMR data: a. Overall fit of wide field EPR scan taken using 'Setup 2' (as detailed in the main text) using the MATLAB toolbox EasySpin[53] with four derivative signals at each angle. **b-c.** The fitted signal component 1 (purely Lorentzian; b) and 2 (purely Voigtian; c) remain centred at fields <100 mT and fit the FMR part of the overall widefield EPR data. **d**. Signal 3 (pure Lorentzian component) also fits the FMR component of the data but unlike components 1 and 2 shows large shifts with orientation of the sample tube. A fourth derivative which fits purely the $g\approx2$ signal is not shown.

We fit the spectrum at each angle to give a coarse understanding of the magnitude and distribution of internal fields in different domains of PNRs present in the inner wall film that give rise to the FMR signal. All fits to the EPR and FMR signals were carried out using the MATLAB toolbox EasySpin[53]. For the derivatives that fit the FMR part of the signal we observe that two signal components (signal 1 and 2) remain centred at fields <100 mT (one pure Lorentzian and one Voigtian component), while one signal

(signal 3 pure Lorentzian component) shows large shifts with orientation of the sample tube (see **Figure S44**).

We determine the net internal field of signal components 1 and 2 to be ≈225-265 mT by taking the difference between the field corresponding to the g≈2 signal and the center of signals 1 and 2. Since the effective resonance position of signals 1 and 2 is at lower magnetic fields compared to a g≈2 signal (which would have negligible internal fields) the net internal fields for these domains must be aligned to the external magnetic field direction. Signals 1 and 2 have large linewidths most likely reflecting the large distribution of internal fields.

In signal 3 the net internal fields are determined by taking the difference between the maximum and minimum effective resonance positions. Since the effective resonance position changes from around 850 mT to 240 mT we estimate the net internal field to be ≈600mT. The linewidth of signal 3 is also broad again suggesting a wide distribution of internal fields. We suggest that the effective resonance positions of signal 3 changing significantly with orientation could be due to these PNRs being hard to (re-)magnetize. This results in the FMR signal at ≈850 mT when the net internal field is anti-aligned to the external magnetic field direction, while the FMR signal at ≈240 mT is due to the net internal field being aligned (or close to aligned) to the external magnetic field direction.

We speculate that the difference in the behaviour of the FMR signals which remain centered at fields <100 mT at all orientations and the signal which shows strong shifts in its resonance field could be due to domains with easy and hard axes. However, the above analysis is simply to give an overview on the magnitudes of net internal fields and to show the presence of large distributions in internal fields present in the sample shown in **Figure 2** of the main text. The above analysis may not necessarily be a unique fit or solution to the experimental data and further measurements beyond the scope of this work are required to fully understand the details of the FMR signals.

EPR Background Impurity Contributions

For both the FMR signal and the g≈2 signal in EPR care is needed due to the possibility of impurities/defects from the quartz tube contributing to the spectra. Generally, we note these impurity/defect signals are usually weak in intensity or rather narrow compared to particularly the g≈2 signal, which has a linewidth ~12-20 mT and can be seen even at low microwave powers. Nonetheless, to further confirm that the reported signals arise from the sample we have performed a variety of control measurements.

i) Control Measurement A: EPR Spectrum of the empty EPR tube (g≈2 signal)

Generally, unless stated otherwise samples in the main text consist of PNR films in the inner wall of a 4 mm diameter EPR tube that is held inside a 5 mm diameter EPR tube. The two tubes are used as the 4 mm tube allowed for more efficient sample preparation (when evaporating solution) but was too short to be held in the EPR spectrometer without using a second tube. Having these two tubes also allowed for easier translation between different angles.

Empty 4 mm tube: Measuring the EPR spectrum from a 4 mm EPR tube alone (**Figure S45**) demonstrates a weak background signal also visible in Figure 2 of the main text, but that is also not overlapped with main PNR g≈2 signal. We can hence already rule out impurities from the 4 mm tube as being a source of the signals we observe.

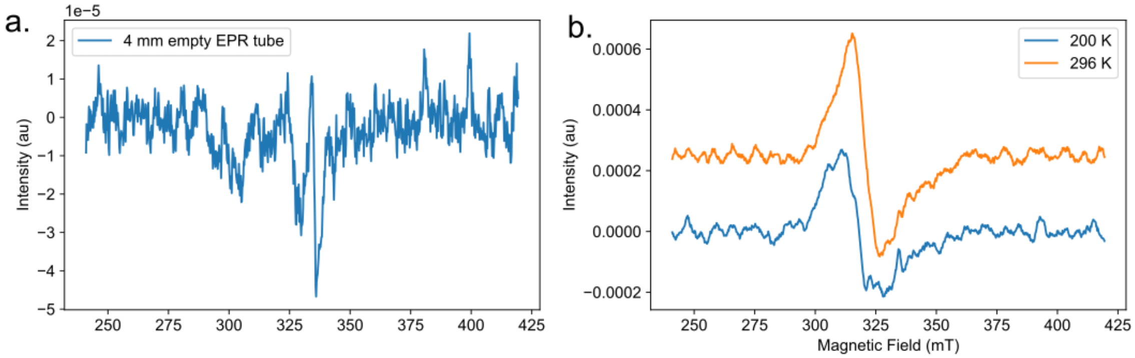

Figure S45 Background signals in EPR 'Setup 1' using a 4 mm diameter EPR tube: a. Background signal from 'Setup 1' (narrow field range) EPR measured using an empty 4 mm EPR tube. **b.** Narrow field range EPR measurement from PNR sample as detailed in Figure 2 of the main text at indicated temperatures. The 'background' signal can be observed around 330 mT and is distinctly narrower than the PNR EPR signal.

Empty 5 mm tube: Comparing the cwEPR spectrum of the sample and the empty 5 mm tube (**Figure S46**) demonstrates a small background in the latter that has a different *g*-factor, intensity and spectral shape to the PNR sample spectra, indicating that the $g\approx2$ signal arises from the sample not the tube. Indeed, as we discuss below these background signals do not arise from the tubes, but the low temperature insert used for EPR measurements.

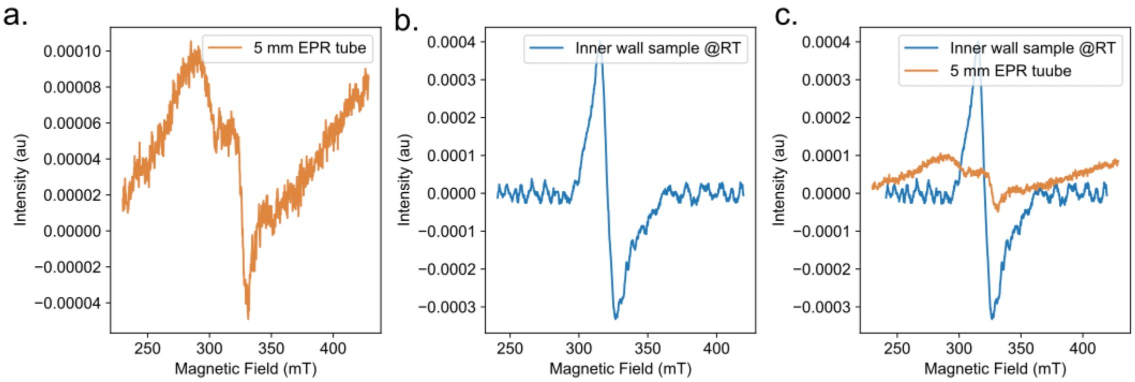

Figure S46 Background signals in EPR 'Setup 1' using a 5 mm diameter EPR tube: a. Background EPR signal from 'Setup 1' (narrow field range) measured using an empty 5 mm EPR tube. **b.** Narrow field range EPR measurement from PNR sample as detailed in **Figure 2** of the main text at room temperature. The sample consists of a PNR inner wall film in a 4 mm tube inside a larger 5 mm tube. **c.** Overlay of spectra in a and b demonstrating the background and $g\approx2$ EPR signal from the sample are spectrally distinct.

ii) Control Measurement B: EPR Spectrum of the Low Temperature Dewar Insert ($g\approx2$ and orientation dependent FMR signal)

Secondly, we perform additional cwEPR measurements using a third setup ('Setup 3') different to that detailed in the main text (narrow ('Setup 1') and wide-field range setups ('Setup 2')). Here, we use a Bruker ELEXSYS E580 spectrometer, with a SHQE X-band cavity. The SHQE cavity is the same type of cavity as used in the narrow field range setup 'Setup 1'. However, this time we use the cavity without the low temperature Dewar insert, as this insert can have background signals. The reason for using the Bruker ELEXSYS E580 is that this allows us to do the full magnetic field sweep using the SHQE resonator. For consistency we perform measurements on the same samples detailed in **Figure 2b** of the main text.

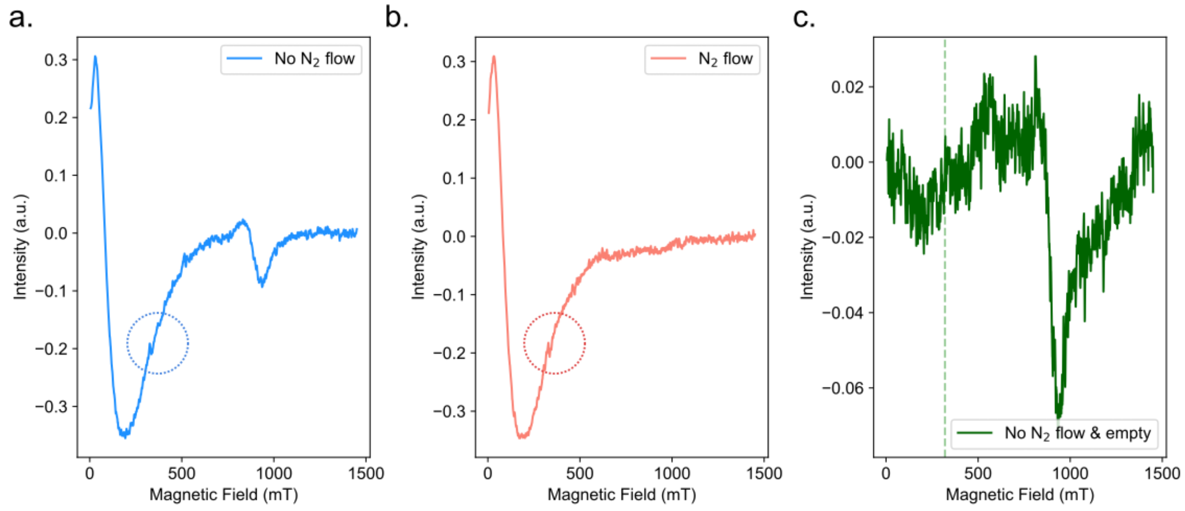

Figure S47 EPR spectrum of inner wall sample with/without inert gas flow: a. EPR spectrum of PNR inner wall sample with no N_2 gas flow inside EPR cavity. **b.** EPR spectrum of PNR inner wall sample with N_2 gas flow inside cavity. In both (a) and (b) the PNRs (inner wall sample) are flame sealed in a 4 mm EPR tube under vacuum. In this setup ('Setup 3'), the omission of the low temperature insert allows direct measurements of the PNR sample in the 4 mm tube (no longer a 4 mm sample tube inside a 5 mm tube). The $g \approx 2$ signal is highlighted with a circle. **c.** cwEPR spectrum of empty 4 mm EPR tube (sealed under 500 mbar Helium atmosphere) without N_2 gas flow through the cavity. Dashed line indicates where the $g \approx 2$ signal of PNRs is. This is clearly not observed in the tube. These measurements are performed in a setup without a low temperature insert, and it is not PNRs that are exposed to air, rather it is the microwave cavity that is either exposed or purged of air. The signal at around 900 mT arises from the presence of air in the microwave cavity.

With this setup we also clearly observe the FMR and $g \approx 2$ signals of the PNR sample (measurements taken at an arbitrary angle) as seen in **Figure S47**. Without N_2 gas flow through the SHQE cavity in this setup (EPR tube flame sealed under vacuum for PNR sample, or helium atmosphere for empty tube) we see a weak signal at ~900 mT not previously seen in the data presented in the manuscript (**Figure S47a**). Upon flowing nitrogen gas through the cavity during the measurement, we find this ~900 mT signal disappears (**Figure S47b**). This shows that the ~900 mT signal arises from the setup when measuring in air.

This is further reinforced by measurements on other PNR 'inner wall' film samples (in 5 mm tubes) and on empty 5 mm tubes. As shown in **Figure S48** (measured using 'Setup 2') the empty tube shows a flat line spectrum, apart from the ~900 mT signal (when the measurement is taken with air inside the microwave resonator). The ~900 mT signal is well separated from the FMR and $g \approx 2$ signals that we discuss in our text and our observations in **Figure S49** and **Figure S50** confirm that both the FMR and $g \approx 2$ signals arise from the PNRs. Furthermore, given that the background signals from 'Setup 1' detailed in **Figure S45** and **Figure S46** are not observed with 'Setup 3' with the only difference being the low temperature insert, our results

in **Figure S47** demonstrate the backgrounds in **Figure S45** and **Figure S46** must arise from the low temperature insert.

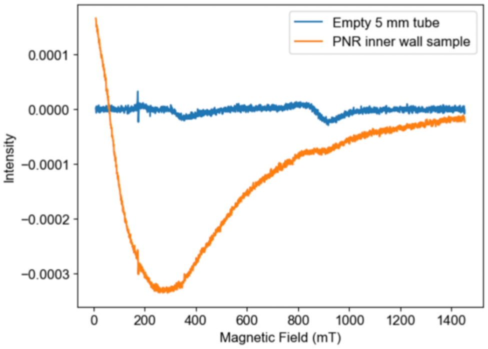

Figure S48 EPR spectrum of empty 5 mm tube compared with inner wall sample: EPR spectrum of a PNR inner wall sample directly made inside a 5 mm EPR tube flame sealed under vacuum (orange) and EPR spectrum of empty 5 mm tube (blue). Both measurements are performed without He flow inside the EPR resonator. Data is unnormalized highlighting the difference in magnitude between signals. This sample is different to that detailed in the main text and does not show a clear $g{\approx}2$ signal, highlighting the sensitivity of this signal to film preparation.

Lastly, we remark that the $g{\approx}2$ signal (predicted for mono/bi-layer PNRs) is highly dependent on the inner wall tube sample preparation. For example, in some (inner wall) PNR samples only the low and high field FMR signal (with a tube orientation dependence) can be observed (see **Figure S49** and **Figure S50**; measurements are done using 'Setup 2').

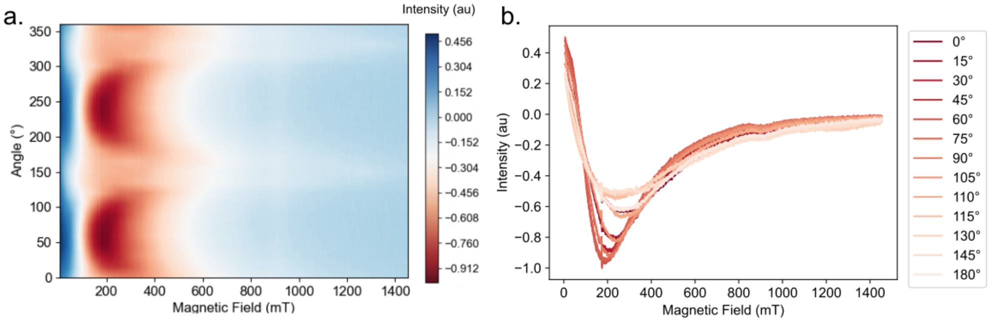

Figure S49 EPR spectrum of inner wall sample as a function of orientation without He flow in resonator: a-b. EPR spectrum of a PNR inner wall sample directly made inside a 5 mm EPR tube flame and sealed under vacuum, *without* He flow inside the EPR resonator. This sample is different to that detailed in the main text and does not show a clear $g{\approx}2$ signal but the FMR signal can be observed. The FMR signal has the same orientation dependence to that in the main text (with maxima/minima at different angles).

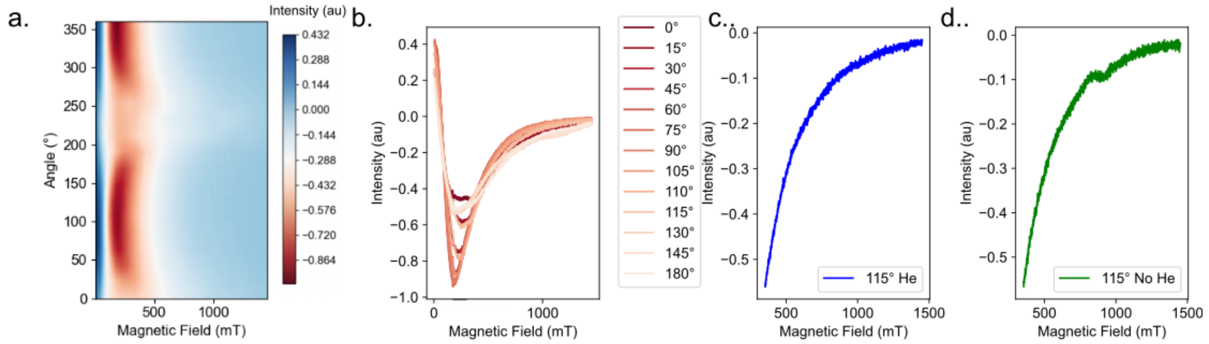

Figure S50 EPR spectrum of inner wall sample as a function of orientation with He flow in resonator: a-b. EPR spectrum of a PNR inner wall sample directly made inside a 5 mm EPR tube flame sealed under vacuum, *with* He flow inside the EPR resonator. This sample is different to that detailed in the main text and does not show a clear $g \approx 2$ signal. The FMR signal has the same orientation dependence to that in the main text (with maxima/minima at different angles). **c-d.** Demonstrating the effect of He flow inside the resonator by zooming into the high field signal.

iii) Control Measurement C: EPR Spectrum of the EPR Tube and MD5 resonator ($g \approx 2$ and orientation dependent FMR signal)

Using 'Setup 2' and performing a wide field scan on an empty 5 mm tube (without He of N_2 gas flow) we find the background signals at 190, 380 and 900 mT (**Figure S51**). The signal at ~190 mT is well known to arise from impurities in the dielectric sapphire ring of the MD5 resonator and can be seen in the original **Figure 2b** dataset and also for the different inner wall sample presented in **Figure S49**. The signal around 380 mT likely arises from the Cu residual within the resonator but we refrain from exact assignment. All the background signals are distinct spectrally from the PNR FMR signal and $g \approx 2$ signal (280-320 mT depending on tube orientation). Furthermore, these background signals have no orientation dependence. Together these observations conclusively demonstrate that the $g \approx 2$ and FMR signals arise from PNRs.

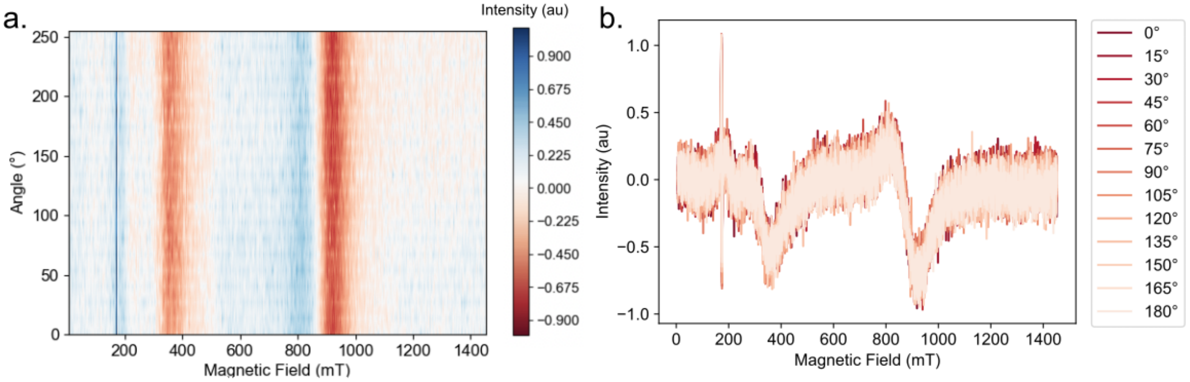

Figure S51 EPR spectrum of empty 5 mm EPR tube as a function of orientation: a-b. Map and line cuts of EPR spectrum of empty 5 mm tube using 'Setup 2' as detailed in the main text. The signals at 190, 380 and 900 mT are distinct spectrally from the PNR FMR signal and $g \approx 2$ signal (280-320 mT depending on tube orientation). Furthermore, these background signals have no orientation dependence unlike that of PNRs.

EPR setup tube and resonator axes

Figure S52 details the two main setups used for EPR measurements and the reference frame of the EPR tube and PNRs as compared to the spectrometer/field axes. The main take away from the rotation dependence is simply to highlight an overall inhomogeneity in inner wall films. This inhomogeneity/anisotropy in the films means that as the tube is rotated along the *y* axis in **Figure S51** some PNR(s) (domains) and their spins (PNR film internal fields) become more or less aligned with respect to the magnetisation (measurement) field direction.

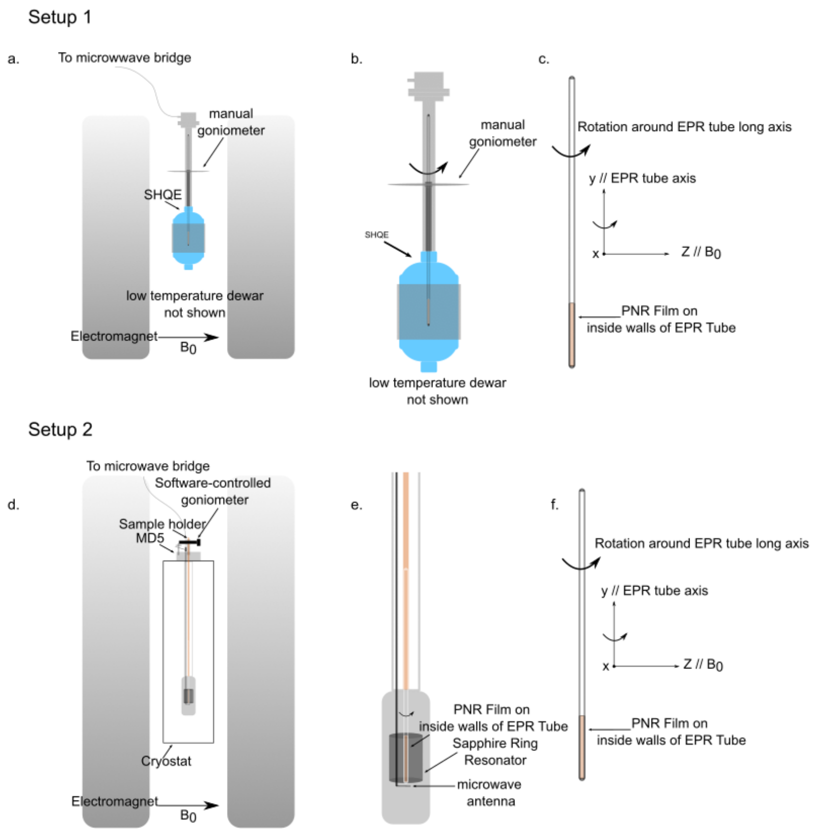

Figure S52 Summary of EPR setups used in the work and tube-resonator orientations: a-f. Cartoon schematic of the EPR setups as described in the methods section of the main text used to measure EPR spectra in narrow and wide magnetic field ranges.

We note that since only part of the EPR signal moves with tube rotation and part of the signal does not (see discussion around **Figure S44**), we suggest the PNR film itself has an easy and hard axis to re-magnetize. If all PNR domains were easy to re-magnetize such that their internal fields are always aligned to the external magnetic field, we would not see an orientation dependency in our measurements. We attempted to further expand on this by holding the same sample as detailed in main text at an orientation of ~105° and magnetic field of ~1.45 T for 1 hr. We then re-ran the cwEPR experiment, but observed no difference in the intensity of the low or high field FMR signals (**Figure S53**).

This indicates that further work is needed to establish domain re-magnetisation in PNR films. We stress that it remains unclear what the nature of domains in edge magnetic systems are as it appears that bulk films of PNRs show properties reminiscent of standard ferromagnetism, but we refrain from overemphasizing such a similarity, which is currently rather superficial and not backed by any theory or models. However, our empirical observations regarding hard and easy axes do remain.

Finally, we comment that the rotation dependence of the PNR EPR signals also allows us to distinguish EPR signals that arise from the PNRs as opposed to any background/impurities which do not have a rotation dependence. Given that we are dealing with an ensemble of PNRs in many of our measurements and trying to be quantitative in our findings it is important that we capture film inhomogeneities, making the rotation dependence important. These measurements allow us to provide upper/lower bounds on the internal fields that we extract for example.

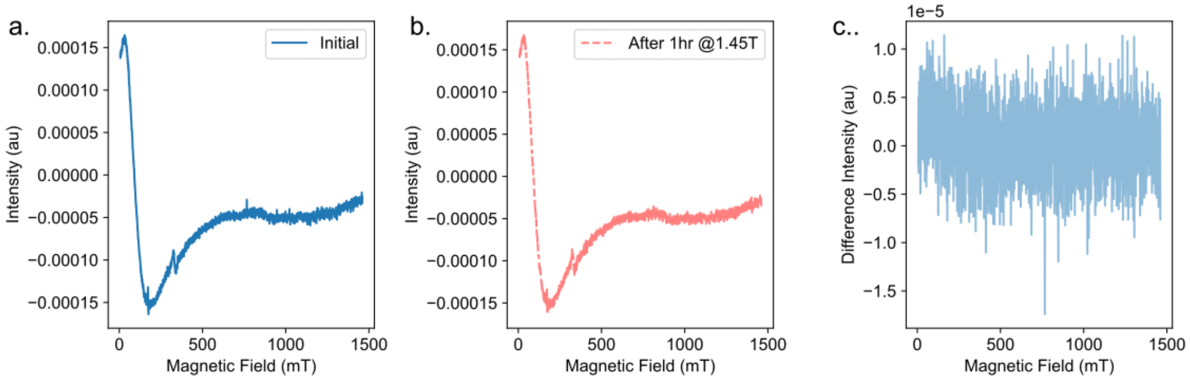

Figure S53 Influence of holding magnetic field on EPR spectrum of PNRs : a-c. CW EPR signal of PNR inner wall sample (sample in main text measured using setup 2), before (a) and after (b) after holding the sample at 1.45 T. **c.** No difference in the EPR signal is observed after holding the sample at 1.45 T for 1 hr.

<u>Effects of overlapping PNRs on the EPR signal</u>

Finally, we note that correlating how the morphology of the PNRs in a given location of the EPR tube links with the signal is challenging. This is due to the curved nature of the EPR tube and the fact that the AFM imaging would require degradation of the sample due to air exposure. We also note that films were prepared by depositing 400 μl of PNRs inside an EPR tube, to see the observed FMR signals. We believe thinner films where PNRs can still stack, would also show a FMR response and magnetic coupling between ribbons, however the strength of the FMR response is strongly dependent on the morphology and stacking of the ribbons. Hence with very thin films FMR signal are likely below the sensitivity of the detector.

However, if we use the thickness of the PNR films prepared in the same manner as for EPR for morphology characterisation (**Figure S20**), we can suggest that the range of internal fields we observe from 220 to 850 mT likely arises due to film thickness increases from of 4.3 to 8 μm. The $g \approx 2$ EPR signal arises from individual PNRs and is also sensitive to the tube orientation as shown in **Figure S41**, and packing/morphology. However, the percentage variation in this signal with orientation is only approximately $\pm 6\%$ of the mean signal. Whereas for the FMR signal this variation is $\pm 50\%$ of the mean signal. If the $g \approx 2$ signal was also influenced by interaction between ribbons we might expect a similar variation as for the FMR signal. Furthermore, our tube orientation dependent g-factors are consistent with

those predicted for mono- and bi-layers of PNRs. The variation of the $g \approx 2$ signal is more in-line with that observed with temperature, which we ascribe to spin canting of PNR edge spins. In other words, it appears that the orientation dependence of the $g \approx 2$ signal is as a consequence of more/less PNRs lying on their side/flat inside the EPR tube and not due to stacking. We hence suggest that the *intrinsic* magnetic properties at the individual PNR level are not influenced by ribbons overlapping with each other.

Supplementary Note 16: EPR spin counting

Estimating the spin polarization length in PNRs is challenging especially from SQUID as we do not observe a clear saturation in our M *vs* H plots when background corrected. We hence rely on our EPR data to quantify the spin population. In EPR the number of spins is determined from the double integral of the (entire) experimental spectrum. Accurate determination of the number of spins requires considering experimental settings, the quality factor of the resonator, and calibration of the setup with a sample of known concentration. It also requires knowledge of the sublevel population/spin polarisation and therefore is usually used only for ground state paramagnetic spin systems as they have a Boltzmann population of the sublevels. Nonetheless, we can perform spin counting of the EPR/FMR measurements taken at room temperature on PNR films as detailed in **Figure 2a** of the main text (105° spectrum). If we take a Boltzmann spin population at room temperature (T = 295 K), we obtain the number of spins contributing to the signal to be ~1.2×10^{20}. However, since we are dealing with a ferromagnetic system, we expect the system to be highly spin polarised. Therefore, to obtain a more realistic estimate of the number of spins we set the Boltzmann population factor to a value corresponding to a lower temperature where the spin polarisation is much greater. As we are dealing with an effectively ferromagnetic system we assume a 100% spin polarization which would be achieved with a Boltzmann factor corresponding to 0.01 K. This then gives the number of spins estimated from cwEPR double integration to be ~4×10^{15} in the entire sample.

In order to estimate the number of spins per PNR in our ensemble distributed sample, we need to make some approximations due to the polydispersity in the PNRs width, height and length. Using the distributions reported in the main text and **Supplementary Note 1**, we can estimate the total 'area' of PNRs in the solution (treating a bilayer as 2 monolayers, etc). Treating the width, height, and length as uncorrelated as done in **Figure 1** of the main text, and using the original ('OG') histograms (see **Supplementary Note 1**), we get a mean area per PNR as A_{OG} = 44330 nm^2. We can compare this value with a naïve mean PNR area by taking the mean magnitude of each PNR dimension (the mean of a product is not the product of the means for parameters with any covariance) which yields, A_{mean} = 11200 nm^2. Finally, we can also determine an average area per PNR from the correlated AFM histograms shown in **Figure S16** which is A_{AFM} = 400538 nm^2. This value is larger than A_{OG} and A_{mean} likely because the lower lateral resolution of AFM/AFM tip convolution effects which potentially bias us towards larger PNRs. Nonetheless, noting that the number of spins per PNR will scale proportionately with the total area for a fixed mass, we can use these various different estimates of the mean PNR areas to set bounds on the expected number of spins per PNR.

With the above in hand we note that the PNR unit cell area is 0.15 nm^2 [54]. From this we can estimate the number of unit cells $NCell_{PNR}$ per PNR using the two areas (A_{AFM} and A_{OG}) as ranging between 2.9×10^5 and 26.5×10^5 unit cells per PNR. Finally noting that each unit cell has 4 P atoms in it and the molar mass of P is Mol_P = 30.97 g/mol, the average PNR molar mass can be estimated as $N_{PNR} \times 4 \times Mol_P$ which ranges from 3.6 x 10^7 to 32.8 x 10^7 to g/mol of PNRs. Hence in the 400 µg PNR sample used in our EPR measurements, using Avagadro's number, we have between 66.3 x 10^{11} and 7.33 x 10^{11} total PNRs. Noting the 400 µg sample mass, we estimate that the number of spins per PNR ranges between 260 and 2400 spins per PNR. Dividing this number by 2 (for 2 long edges) and noting that the zigzag edge-to-edge P atomic distance is 0.32 nm, this yields a value for the average spin polarisation length for PNRs in our ensemble of between 42 and 377 nm. This number comes with several caveats and limitations e.g., estimates on the area of PNRs and incomplete knowledge on the precise spin population giving rise to the signal, etc. However, it does allow us to generate a rough estimate of the number of spins likely involved in the magnetisation.

Supplementary Note 17: Persistence Length Measurements

With regards to the magnetic anisotropy measured in solution, we note that any ribbon bending will naturally influence the interpretation that we make. We can consider the types of deformation that might occur in a PNR from **Figure S54** and quantify the resulting influence from the persistence length. A discussion of the fitting model itself is detailed in **Supplementary Note 6**.

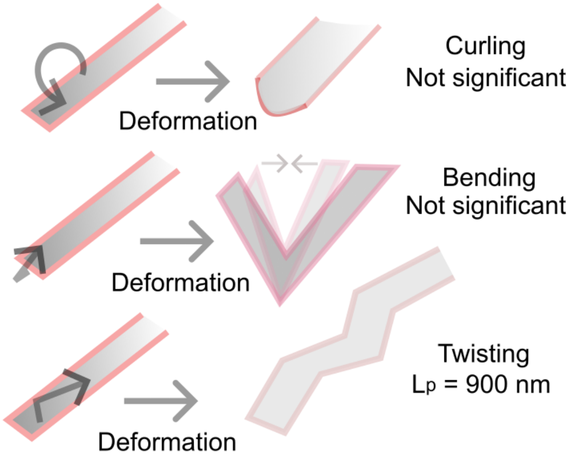

Figure S54 Types of structural deformation that could be imagined to occur to PNRs in solution: Curling and bending of PNRs upon themselves are not found to be significant, but some twisting and deviation from a rigid rod is found. The persistence length (L_p) of a PNR along its long-axis is found to be 900 nm.

From AFM data the persistence length in the long axis of the ribbon is too large to measure. In the layer axis (the height axis of the AFM images) the ribbons are indeed flexible (see **Figure S5**). However, quantifying this is very difficult because ribbons conform essentially perfectly to the HOPG contours and step edges (see **Figure S7**). Even an approximately 10 nm step in the HOPG seems to pose no conformational problem for the ribbon. Indeed, we measured the slope of the step edge on the bare HOPG on which PNRs are cast and compared this to the slope on the ribbon. The two correlate very well (**Figure S8**), implying that the ribbon has no trouble bending to match the underlying HOPG step. When depositing PNRs for AFM however, we find they always lie flat. Although these measurements on substrate are not the same as in solution the fact that the samples deposited from the solution do not show bending or curling effects (as pictorially described in **Figure S54**), means we can as a first approximation suggest they will not be significant.

To estimate the 'twisting' of PNRs (bottom panel of **Figure S54**) we make use of the TEM images of the ribbons from ref[1]. Focussing first on the long axis of a PNR we can estimate roughly from TEM images that they begin to twist after >500 nm. Dividing a PNR into segments of length 3 nm based on the TEM resolution we can calculate the tangent vectors of each segment. Fitting an exponential decay to the autocorrelation in angle between tangent vectors, $\langle \cos(\vartheta_i) \cos(\vartheta_j) \rangle$, the long-axis persistence length can be estimated (L_p). Across PNRs a $\langle L_p \rangle$ of ~900 nm is estimated (**Figure S55**). This is a simple estimate based on a worm-like chain (as also used for graphene nanoribbons[55]). Like AFM, TEM cannot estimate the bending in the layer axis. However, qualitatively in our TEM measurements/images we also find little to no evidence for PNRs bending (or curling). If this was occurring in solution, we would expect to be depositing tube like or folded PNRs onto the TEM grids as opposed to mostly flat PNRs. We consequently

take there to be little to no curling/bending in solution of the PNRs and do not consider this effect on our magnetic calculations, only focussing on L_p which we can estimate.

With all this in mind we can consider the influence of twisting on our magnetic anisotropies using the theory initially designed for polymers in magnetic fields. Generally, if the segment length of a chain like object l_s is longer than the object itself, L, the magnetic anisotropy $\Delta\chi$ should be multiplied by a factor l_s/l_0 where l_0 is the monomer length. This correction accounts for the deviation away from a rigid rod. In terms of persistence lengths Weill has determined[56,57] that the correction factor should be $\frac{2}{3}\frac{L_p}{l_0}[1-\frac{L_p}{3L}(1-e^{-\frac{3L}{L_p}})]$. However, as explained by G. Maret and K. Dransfeld in ref[56,57] this correction only needs to be applied if $L >> L_p$ i.e., the PNRs are longer than their persistence length. As shown by comparing histograms in **Figure S1**, **Figure S54** and **Figure 1c** of the main text the mean length of PNRs we study is ~620 nm which is significantly shorter than the persistence length (900 nm). Consequently, the overall rigid rod approximation we apply is valid.

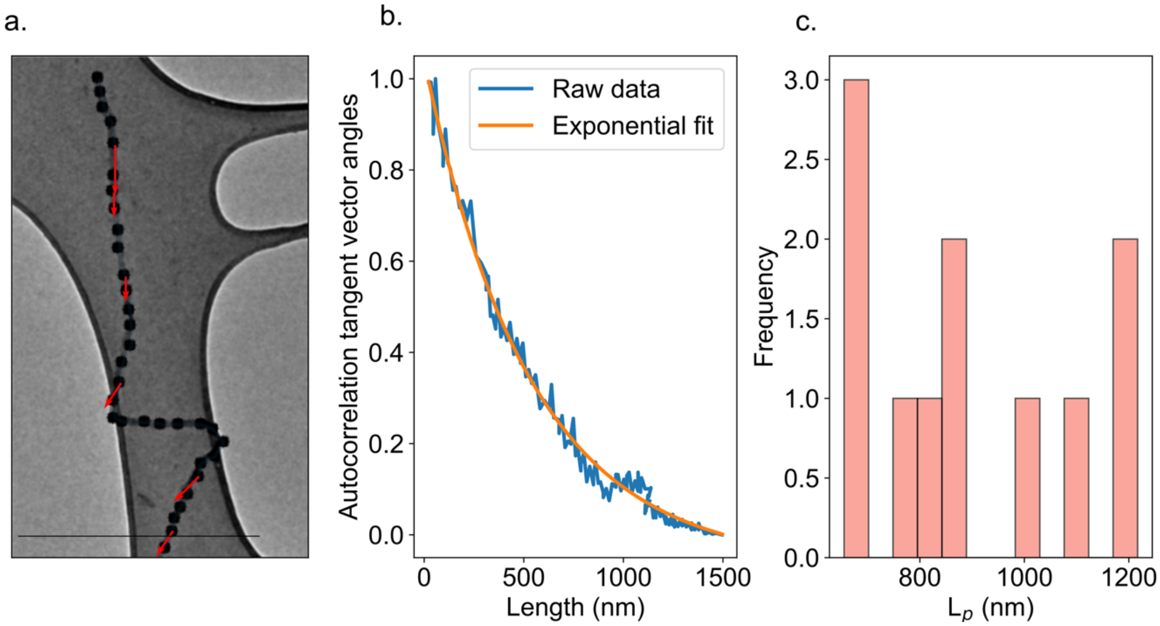

Figure S54 Estimating the persistence length of PNRs:. **a.** TEM image of PNRs from ref[1,2] with sub-set of segments and tangent vectors marked. Scale bar is 900 nm. **b.** Autocorrelation of tangent vector angles along PNR with associated exponential fit. Exponent gives the long-axis persistence length, L_p. **c.** Histogram of PNR L_p values across 11 ribbons. The mean L_p is taken for any subsequent analysis.

Supplementary Note 18: Single ribbon steady-state and transient optical micro-spectroscopy

To make a more robust connection between the ensemble level PNR properties that we report, and the properties of individual nanoribbons (which are typically captured by theory), we conduct a range of microscopic measurements on individual PNRs. We perform high-resolution photoluminescence (PL) microscopy, polarisation resolved pump probe microscopy and Raman microscopy on individual nanoribbons. These measurements provide insight into the magnitude and origin of the line broadening in our optical measurements.

Single Ribbon PL Microscopy

To get some understanding of the link between PNR width and the semiconducting properties we can perform high-resolution photoluminescence imaging and spectroscopy using a spinning disk confocal microscope with a lateral resolution of ~115 nm. In imaging experiments, PL is collected in the 550 to 800 nm spectral range with excitation at 390 nm. We caveat that the optical diffraction limit means we can only apply such methods to a sub-set of PNRs much larger than dominant width present in the ensemble. Indeed, the limited number of such large PNRs within the samples means limited statistics can be obtained from such measurements.

In **Figure S55** we show PL images from individual PNRs 150 to 600 nm in width alongside spectra from a 0.1 μm^2 area of the PNR marked with an asterisk. For each of the individual PNRs we find a doubly peaked spectrum where the low-energy peak is significantly (3-10 times) stronger than the peak at high energies, consistent with ensemble PL measurements detailed in **Supplementary Note 20**. Interestingly as the PNR width increases the centre of the main the emission peak moves to lower energies. This suggests some degree of confinement across the PNR width, with narrower PNRs having higher energy emission at least for the central peak. Indeed, the fact that the PL of the individual PNRs is all red shifted from that of the main ensemble PL peak can be reconciled by the fact that in our single object spectroscopy we are measuring PNRs with larger widths than the average width in the ensemble (~10 nm). The above results are in-line with theory that suggests the bandgap widens with decreasing sizes of PNRs, but it is somewhat surprising to observe this effect at large PNR widths[58,59].

The PL linewidth from the individual (large) PNRs (~0.07 eV) does not seem to be significantly influenced by the PNR width. It can hence be suggested that the linewidth will also be invariant for the narrow sub-15 nm width PNRs which dominate the ensemble. Assuming this to be the case, we suggest the 3.5 times broadening between the ensemble PL spectra **Supplementary Note 20** (~0.25 eV) arises from PNRs of different length or thickness (though we note the sample is dominated by monolayers).

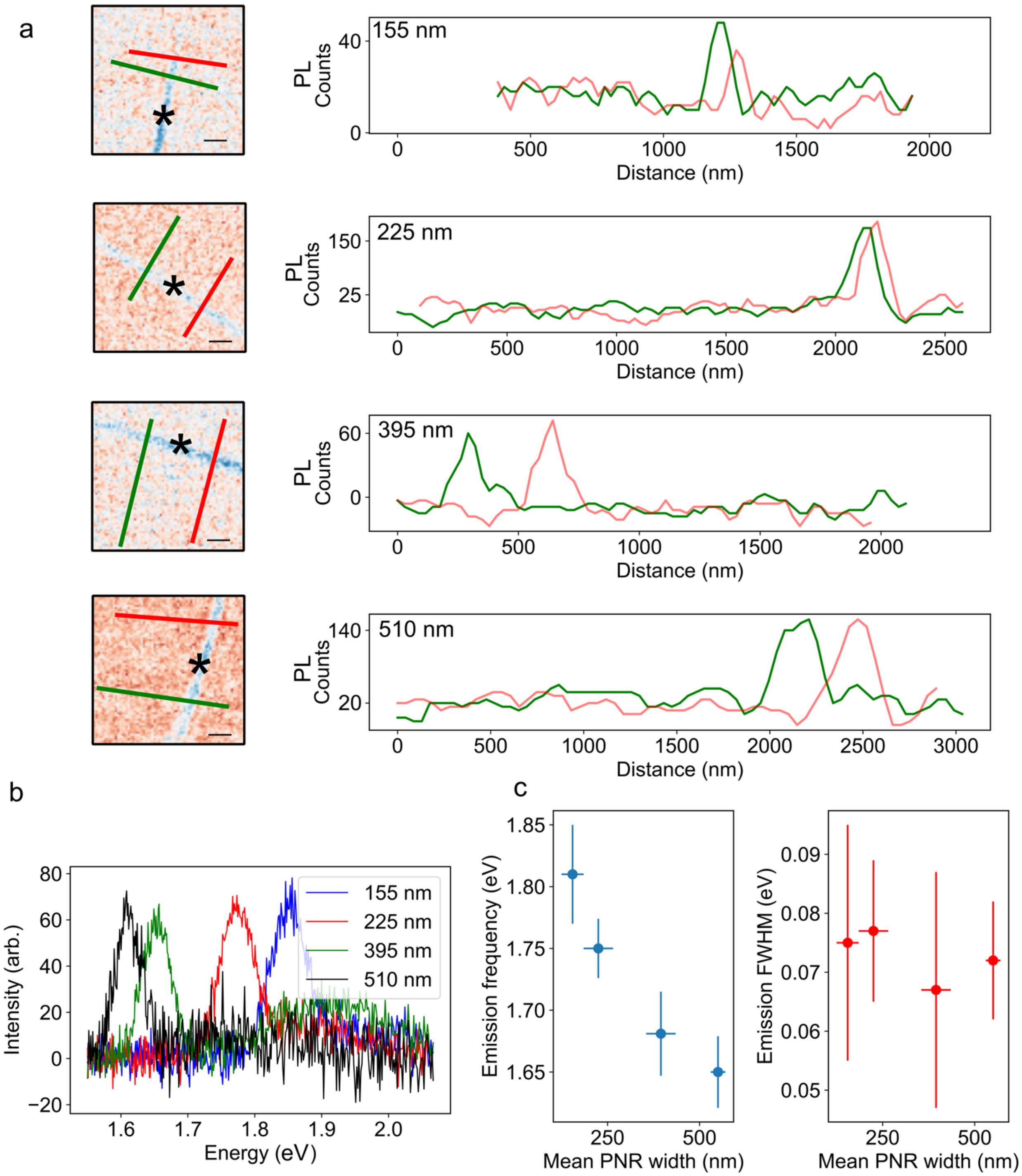

Figure S55 Single PNR emission spectra: a. (Left) Photoluminescence images of PNRs integrating emission between 600 and 800 nm. (Right) Emission line cuts of PL images for lines marked in red and green on images. The nominal width of the PNRs, estimated from the full-width half maximum of the line-cut, increases down the column. Gaussian smoothing is applied to the image before calculation of the line-cuts but in such a way that the estimated error in width does not increase outside the bounds shown in (c). The asterisk marks where spectra are taken from on these PNRs (we note the spectra empirically do not vary within our resolution along the PNR). Scale bars top to bottom are: 300 nm, 400 nm, 500 nm and 600

nm **b.** Emission spectra of PNRs as a function of width (legend). **c.** Centre-frequency of emission and emission linewidth (FHWM) against PNR mean width. Error bars are derived from measurements on between 5 to 7 distinct PNRs each.

Finally, we remark that precisely correlating our ribbon widths with the PL spectra is challenging. This is because samples must be protected from oxygen (by encapsulation prior to imaging) preventing post-PL AFM measurements. Nonetheless, based on the AFM characterisation detailed in **Supplementary Note 1**, we can reasonably assume the PNRs measured in the single object spectroscopy measurements are mono or bilayers.

Individual Ribbon Absorption

Absorption spectroscopy of individual PNRs >300 nm in width was also performed using a customised Zeiss Axio microscope (see **Methods**). A broad distribution of spectra are observed with band-edges between 500 and 800 nm potentially explaining the broad tail observed in the ensemble absorption spectrum. The spectrum is relatively featureless regardless of ribbon. Like for the single PNR PL imaging however we emphasise that we can only image wide PNRs which do not represent well the average PNR width found. Nonetheless, these results demonstrate the absorption linewidth is likely dominated by inhomogeneous broadening effects.

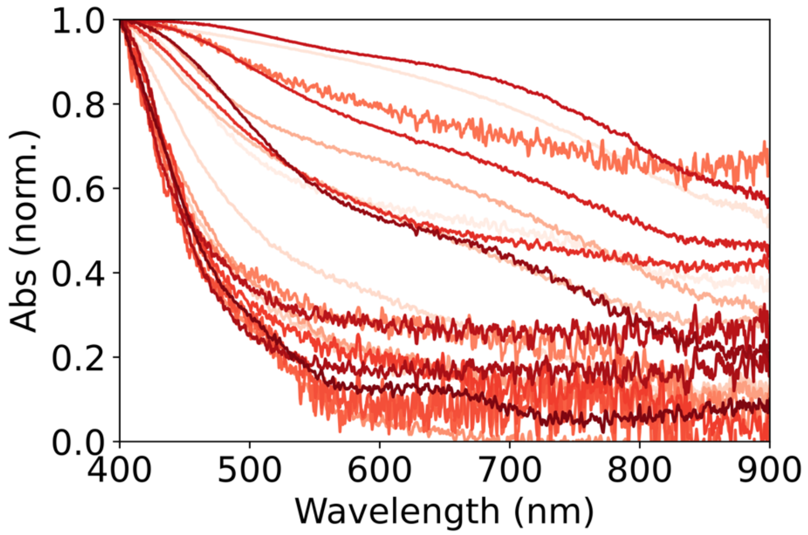

Figure S56 Absorption spectra of individual PNRs: Absorption spectra of individual (large; >300 nm) PNRs.

Single Ribbon Pump-Probe Microscopy

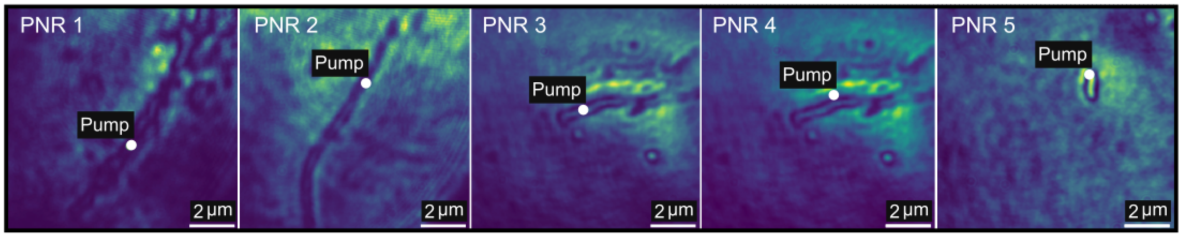

Figure S57 Images of individual ~300 nm wide PNRs measured using pump-probe microscopy: White dot shows where the pump pulse is focussed in experiments.

To understand whether the broad photoinduced absorption features in **Figure 3a** arise from lifetime (homogeneous) broadening or due to a range of widths in the ensemble, we can perform fs pump probe spectroscopy on individual PNRs with widths of ~300 nm as shown in **Figure S57**. To do so we perform sub-15 fs pump-probe microscopy (transmission geometry), where pump and probe pulses are focussed onto PNRs in the locations marked, with the broadband spectra collected (see previous works from ourselves[60,61] and the **Methods**).

All the PNRs we image are around ~300 nm in width hence we do not correlate spectra/dynamics with the PNR widths as we do not have the resolution to do so. Our pump probe microscopy experiment has poorer spectral resolution (2-3 nm *vs* sub-1 nm) than our broadband pump probe experiments, and is limited to the 600-900 nm collection range, i.e., we are mostly sensitive to the dynamics of ESA 1. We note that a significant challenge presented by these measurements is that the measurement time must be reduced to ensure the lateral drift of the microscope is smaller than the ribbon width.

Examining 5 PNRs we find spectra centred at a range of wavelengths between 650 and 800 nm as shown in **Figure S58**, but all spectra have a derivative-like line shape at early times (<200 fs). After ~150 fs the positive derivative feature decays away and there is a negative $\Delta T/T$ feature left. Spectrally, this behaviour matches very well with the ESA 1 feature observed in the ensemble measurements highlighted in **Figure 3a**. The original early time derivative-like feature (which we do not comment upon in the main text) likely arises from energy renormalisation effects as has been well-documented for other 2D semiconductors[62]. Briefly, this is where photoexcitation results in increased electronic screening and renormalisation of both the binding energy and bandgap. The former typically redshifts the optical spectra whereas the latter will blue shifts the spectra. The balance of these two effects will overall determine the absolute shift in the spectra. In any case a blue or red shift in the ground state absorption will result in a derivative-like shape in the transient spectra. Following carrier cooling the derivative-like lineshape will disappear to give rise to signatures of other carrier dynamics in the transient spectra. This behaviour indeed appears to be present from the individual and ensemble PNR transient absorption spectra. The fact that the derivative lineshape has its positive feature at lower energies suggests that binding energy (as opposed to band gap) renormalisation is the dominant effect that occurs following PNR photoexcitation.

Another noteworthy features in the single PNR pump-probe spectra is that in some cases (PNR 4 and 5) there is also/only a positive lobe blue shifted to ESA1 at early times, which also appears in the ensemble pump-probe spectra. This could be from stimulated emission (the emission of PNRs lies in a similar spectral range as shown in **Figure S55**) or other electronic/excitonic absorptions.

After 500 fs the dominant feature in the pump-probe spectra of the individual PNRs is an excited state absorption. The key difference between the ESA 1 feature in the single ribbon pump-probe spectra and that of the ensemble is that for individual PNRs ESA 1 is narrower (200 meV *versus* 600 meV) in the single PNR measurement that the ensemble data. This demonstrates that the broad excited state absorption band in the ensemble may, to an extent, be as a result of some inhomogeneous broadening. Comparing the ESA 1 decay kinetics (fitting a simple mono-exponential decay for the sub-2.5 ps dynamics) for each of the PNRs shows interestingly a range of lifetimes from 500 fs to 2 ps, with the ensemble decay kinetics over this time period lying at the centre of this range. This means that the lifetimes we report from measurements in solution are also likely partially inhomogeneously broadened and reflect an ensemble average. We note that some sub-structure can also be observed in the ESA 1 feature of the individual PNRs, however as this may well be as a result of the decay of the positive derivative feature as opposed to a given state we do not attempt to interpret this at this stage.

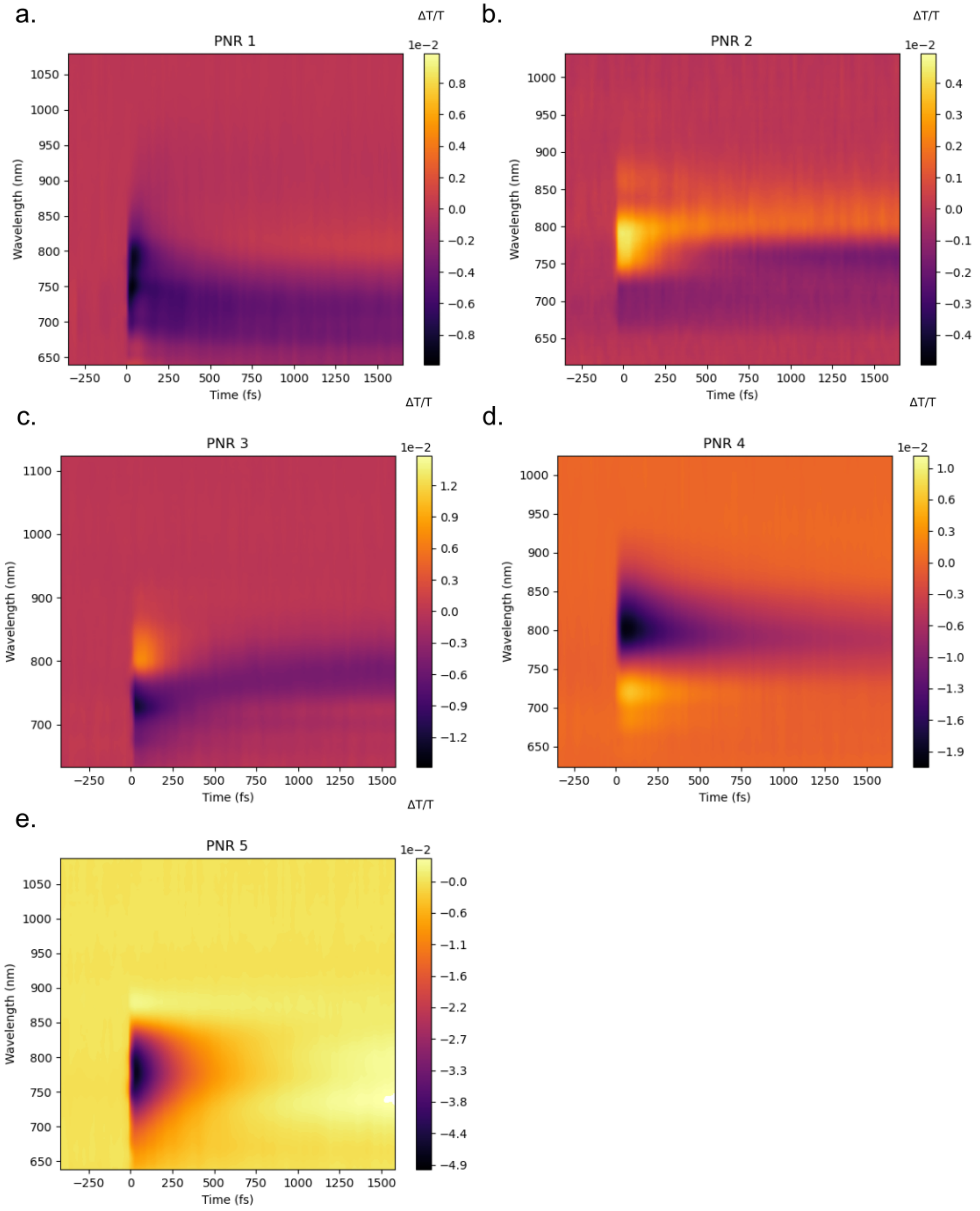

Figure S58 Pump-probe spectra of PNRs 1-5: The number designation corresponds to PNRs in **Figure S57**.

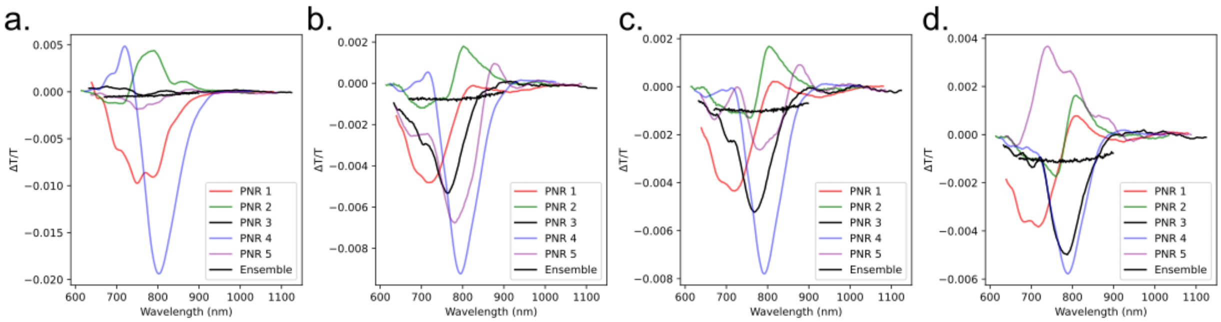

Figure S59 Transient absorption spectra of individual PNRs: a-d. Pump-probe spectra of PNRs 1-5 at time delays of 25 fs (a), 500 fs (b), 800 fs (c) and 1500 fs (d) along with the ensemble (solution) pump-probe spectrum (black) at corresponding time delays.

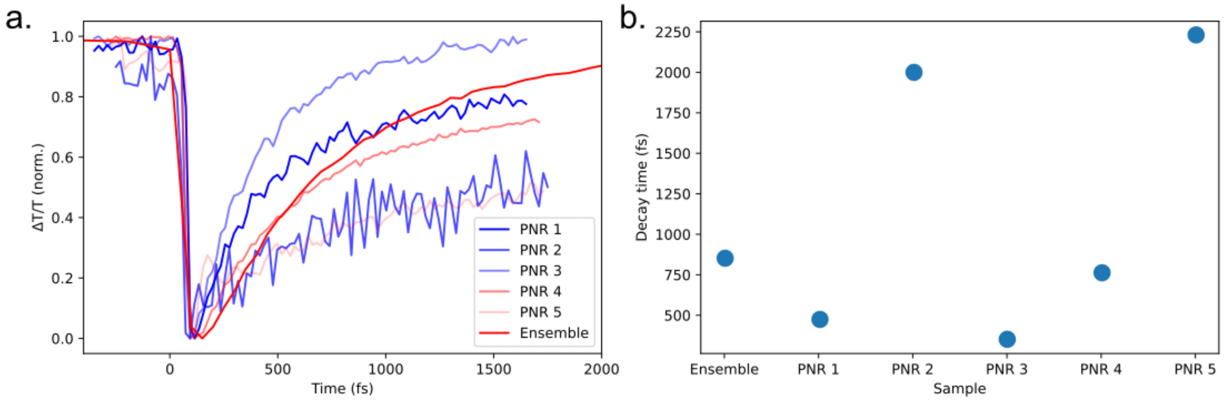

Figure S60 Transient absorption kinetics of individual PNRs: a. Normalised decay kinetics of ESA 1 from PNRs 1-5 along with decay kinetics from ensemble of PNRs in solution. **b.** $t_{50\%}$ for ESA 1 for PNRs 1-5 and the solution. Approximately 15% fitting error on the values.

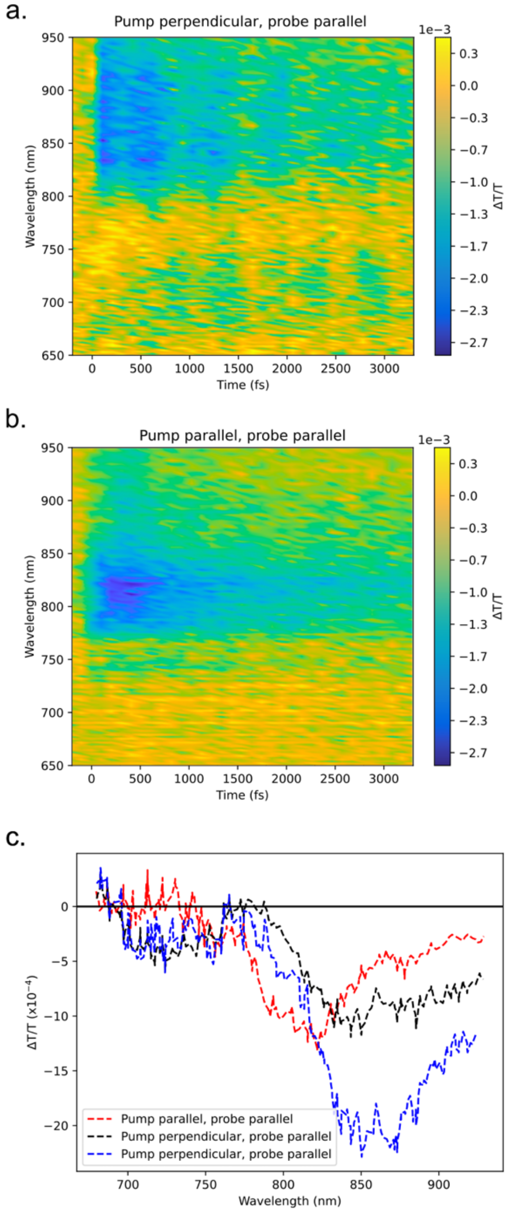

Figure S61 Polarisation resolved transient absorption spectrum of an individual PNR: a-b. Pump-probe spectra from PNR 1 with (a) pump and probe polarised perpendicular to the PNR length (b) pump polarised perpendicular to the PNR length, and polarised parallel to length. **c.** Line cuts of pump-probe spectra from (a) and (b) (red and blue, respectively), alongside the weighted sum of spectra (black).

Finally, we note that the phosphorene lattice is highly anisotropic. At the ensemble solution level in the absence of a magnetic field our pump probe spectroscopy measurements will be insensitive to this i.e., the signals will not depend on the polarisation of light as we demonstrate in **Figure 3** of the main text. However, at the individual PNR level when cast on a substrate, in the absence of a field the spectra will be sensitive to the light polarisation and orientation of the PNR on the substrate. In **Figure S61** the pump probe microscopy measurements are performed with the pump and probe polarised perpendicular to the PNR length. If, however we take PNR 1 and rotate the probe polarisation such that the pump polarisation is

perpendicular to the PNR length and the probe is parallel, ESA 1 both blue shifts and 'splits' to reveal a second weak ESA feature at 700 nm. The width of these ESAs and the weighted sum of the perpendicular-perpendicular and perpendicular-parallel spectra is still narrower than the ensemble ESA (**Figure S60c**), suggesting that inhomogeneous broadening still plays a key role in the broadness of the features in **Figure 3a**. However, additional polarisation dependent transitions which are challenging to detect in the ensemble may also play a role. We note that we are unable to rotate the probe polarization with respect to the ribbon due to limitations of our pump-probe microscope setup. Interestingly, there is limited change in the width of the ESA features (in solution) on application of the field (in which case such polarisation dependent transitions would be revealed), this suggests that they may only be present for a subset of (long and wide) PNRs.

Single Ribbon Raman Microscopy

We cannot resolve individual PNRs on a substrate (or their dimensions), however if the Raman linewidth was inhomogeneously broadened we would expect that measurements over a series of different sample locations would give rise to a range of linewidths. To test this, we have performed Raman measurements across 53 sample locations where the edge mode could be resolved with sufficiently high signal (**Figure S62a**). We find no statistically significant variation in the edge mode (B_{3g}^1) linewidths which across all locations is 4.5 ± 1 cm^{-1} (resolution <0.5 cm^{-1t}) suggesting that this mode is not influenced by the distribution of PNR widths/lengths to within the laser spot size. This is in-line with theoretical calculations which suggest that only at sub-5 nm lengthscales do confinement effects influence phonon modes in phosphorenes[24].

Finally, we can perform Raman spectroscopy along several (6) large PNRs which are greater than 500 nm wide and 1 μm long (likely multilayer). Across 10,000 pixels where the Raman spectrum are measured on these PNRs we find the frequency of the A_g^2 mode varies by only ±15 cm^{-1}. While these measurements have poor spectral resolution (± 2 cm^{-1}) as we also show from our AFM images, e.g., in **Figure 1** of the main text, they highlight that PNRs are exceptionally structurally uniform. Furthermore, the distribution of mode frequencies is non-Gaussian, suggesting the variation might arise from something other than the sampling limitations e.g., underlying strain imposed by the substrate on the PNR.

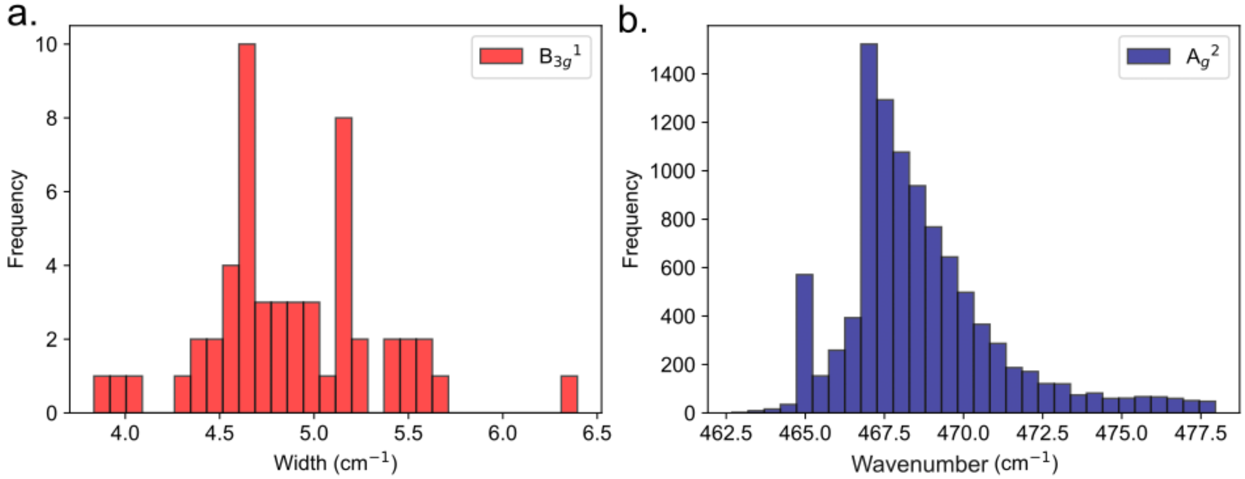

Figure S62 Raman linewidths and centre frequencies of PNR Raman modes samples absorption kinetics of individual PNRs: a. Histogram of width of B_{3g}^1 of modes measured at 53 locations on a film of PNRs drop cast on Si substrate (1 mg/ml concentration solution 50 µl dropped and dried under Ar before being encapsulated with a glass coverslip). **b.** Histogram of A_g^2 Raman mode centre frequency from 6 large

PNRs which are greater than 500 nm wide and 1 μm long (likely multilayer); 10,000 spectra are taken in total to make up the histogram.

Supplementary Note 19: Pressure Dependent Absorption Spectroscopy

Investigating strain on the magnetic properties of ribbons is exceptionally challenging. For example, one might conceive of preparing a pressure cell with which to perform SQUID magnetometery but given the low signals from ribbons and large diamagnetic background any cell would impart, such measurements would be at strong risk from artifacts.

Similarly strain could be measured from our PNRs using scanning area diffraction (SAED) data to find out how much the lattice differs from theory and between different points on the substrate. However, because the degree of strain theoretically suggested to influence the magnetic properties is small[63] and must be applied in specific directions not always accessible inside TEMs used for SAED, this route is not viable. Indeed, such measurements, typically performed on TEM grids, would rely on positioning PNRs on these substrates controllably, an exceptionally challenging task given that PNRs tend to lie around rather than over holes in the grids.

We note that our magnetic measurements are not dependent on any underlying model and we would not go so far as to invalidate our measurements because the samples are more strained than theoretically predicted. Further, and perhaps more importantly, as shown in our SQUID measurements in **Supplementary Note 10**, we observe near uniform magnetic behaviour in frozen solution-based measurements (PNRs frozen in DMF and NMP) as well as on both plastic and quartz substrates. It is reasonable to assume that these different environments have substantially big differences in strain, suggesting that the magnetic phase that we are sensitive to is perhaps more resilient to strain.

One could also estimate the strain along the ribbon length by comparing the shift in Raman mode frequencies at different points along a single PNR (see **Figure S62** and **Supplementary Note 18**). But this requires calculations of the Raman modes and their interaction with substrate, which is beyond the scope of this study.

We therefore focus on the influence of strain on the semi-conducting properties of PNRs. Indeed, in black phosphorous (BP) strain is known to influence the optoelectronic performance strongly[20]. To investigate the influence of strain in PNRs we perform pressure dependent UV-Vis absorption measurements up to 300 MPa (see the methods in the main text). We observe a monotonic blue-shift in the absorption edge of ~300 meV on increasing the pressure from 0 to 300 MPa. The direction of this trend is similar to that of monolayer phosphorene whose band-edge blue shifts on application of ~500 MPa pressures (multi-layer phosphorene and bulk black phosphorous demonstrate a opposite, red-shift of the band-edge on application of pressure[64]). Qualitatively, this behaviour can be rationalised (as was done for phosphorene) by noting that the application of pressure will reduce the P-P bond length, which in-turn will increase the overlap integrals of the valence and conduction band[65]. The energy of the former rises more than the latter causing the blue-shift. In phosphorene and bulk/multilayer BP the shifts in band-edge with pressure are ~80 meV on the application of 500 MPa, whereas in PNRs they are almost 6 times that for a similar amount of pressure. The fact that band-energy change per unit pressure is larger in PNRs, suggests their optical properties are much more readily influenced by strain.

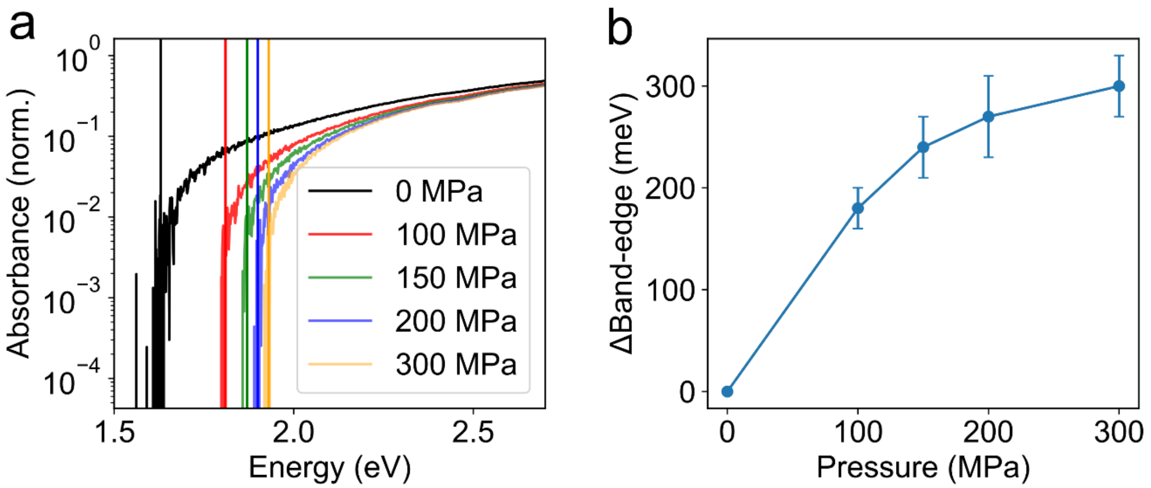

Figure S63 Pressure dependence of PNR absorption: a. Pressure dependent UV-Vis absorption spectra of PNRs as a function of pressure. The vertical lines show the absorption-tail edge from which we can approximate the band-edge. Each spectrum is normalised to the absorption at 3.1 eV. **b.** Shift in band-edge as compared to ambient pressure. The band-edge is inferred from the position at which the gradient of absorbance *vs* energy tends to infinity (error bars represent error in estimation of this quantity). The band-edge shifts drastically with pressure.

We note that while we have provided a qualitative picture of the influence of pressure. To extract parameters such as the in-plane charge hopping parameter, further measurements would be required where PNRs are aligned with respect to the pressure application axis and on multi-layer PNRs. These measurements are extremely challenging because they typically require relatively large (micron area) samples.

We finally remark that the strain behaviour we observe is in line with theoretical predictions for PNRs which indicate that strain should increase the bandgap due to change in the P-P bond length and relative position of bonding and anti-bonding states[63]. These calculations further predict that the ratio of the effective mass between electrons and holes also rapidly increases with strain, providing routes to switching devices. With regards to the topological phase transition that is predicted to occur when PNRs are subject to tensile strain[63,66,67] we do not observe any obvious discontinuities in the absorption *vs* pressure graphs that could be indicative of such a transition. But remark higher pressures and more controlled application of pressure are likely needed. While investigating all these effects experimentally is beyond the scope of this work, our measurements lay the foundation for testing such ideas in future studies and already begin to validate some of these theories.

Supplementary Note 20: Ensemble photoluminescence (PL) spectroscopy

Temperature dependent PL spectroscopy

For ensemble PL measurements PNRs (in DMF) were drop cast on SiO_2 substrates to form an inhomogeneous 1 to 2-micron-thick film. Ensemble measurements were performed with a pulsed laser at 416 nm with a repetition rate of 82 MHz. The laser power at the sample was varied between 5 and 800 μW, with a laser focus of around 1 μm.

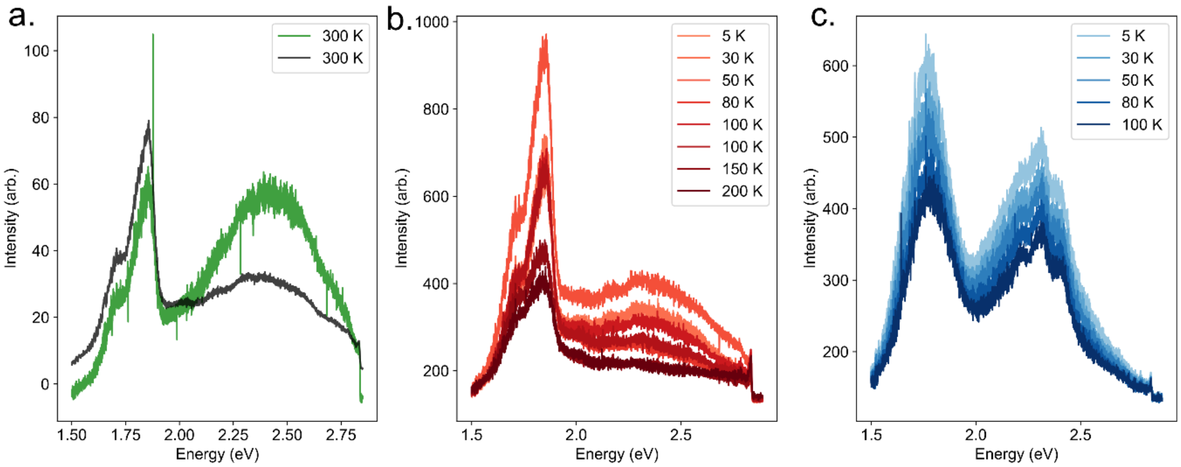

Figure S64 Emission spectra of dropcast PNRs as a function of temperature: a. Characteristic emission spectra of drop cast PNRs at room temperature. A similar shape in the emission spectrum is observed between different locations with variation in the intensity ratio (black and green curves are locations of different sample density). **b-c.** Temperature dependence of spectra from different sample locations. Qualitatively in (b) the density of PNRs is higher and (c) lower.

The PL spectra as shown in **Figure S64** from various locations of the film all have the either one of two characteristic spectra consisting of two broad peaks centred around 1.8 eV and 2.3 eV. Repeat measurements performed over multiple PNR batches and films reveal the same behaviour with the spectra in **Figure S64c** typically from low density regions of PNRs and **Figure S64b** from higher densities. Typically, the lower energy peak has a higher intensity than that at high energies and is narrower. This variation in spectral position of the peaks is potentially due to interaction with the local substrate/environment (other PNRs) which has previously reported to shift the emission energies in mono and few layer black phosphorous (bP)[68] or reabsorption effects. In all cases some sub-structure is resolved within the emission bands which may be arising from an excitonic ladder of states as has been reported for monolayer bP[69], albeit not directly from emission measurements. Indeed, the ensemble emission spectra of PNRs is somewhat similar to that which has been observed for phosphorene. However, in phosphorene the two peaks are centred around 2.0 eV and 1.8 eV, i.e., less separated[70]. In phosphorene the assignment of the peaks remains controversial. The higher energy peak (A) has been assigned to exciton emission whereas the lower energy emission has been assigned to either trions or localised defect states (X). The A:X intensity ratio can vary between 6 and 0.33 depending on the sample and report[71]. As such assigning the spectra we observe for PNRs is non-trivial. Performing power dependent emission measurements shows that: (i) for what would be the equivalent A and X peaks in PNRs the peak intensity ratio remains constant as a function of power; (ii) saturates at the same power; and (iii) follows a linear behaviour up to the saturation turn-on point. This suggests the emission arises from the same state and is less likely to be from trions which would likely show a non-linear power dependence in the emission intensity, i.e., the A and X terminology are likely not appropriate. Exposing the samples to oxygen for 3 hrs, an approach that has been used to engineer

oxygen defects in phosphorene[72], quenches all emission, suggesting that such defects are also not likely responsible for the PNR emission bands.

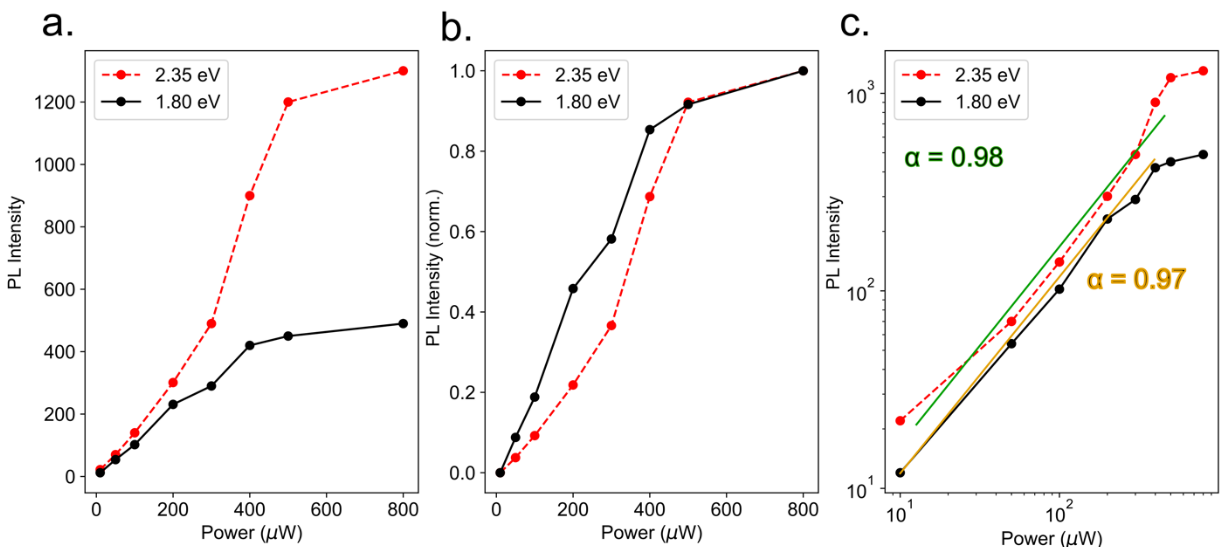

Figure S65 Evolution of PNR emission intensity with temperature and pump power: a. Integrated intensity of PL peaks at 2.35 eV and 1.80 eV as a function of laser power. **b.** Normalised integrated intensity of PL peaks at 2.35 eV and 1.80 eV as a function of laser power. **c.** Log-log plot of integrated intensity of PL peaks at 2.35 eV and 1.80 eV as a function of laser power. A linear relationship between the power and integrated intensity is observed up to the saturation power. A straight line fit (using points before the PL saturation) reveals an almost identical gradient (α) in the power-intensity relationship for the two peaks.

As shown by the histograms in **Figure S1**, the PNR samples we study are not purely monolayer but around 70% monolayer (total of more than 90% mono (ML) or bi-layer (BL)). Hence, it could be argued that the emission bands arise from different thicknesses within the ensemble. However, the quantum yield of ML phosphorene is higher than that of BL, which is red shifted compared to the ML emission[73]. And in addition, our samples are dominated by ML PNRs. Consequently, if the two bands arose from different thicknesses, we would expect the lower energy band to be lower in intensity that the high energy one, which is not the case. Hence, whilst inhomogeneous structural broadening likely plays some role in the emission spectra observed (see discussion below). It is unlikely the specific structure we observe arises from different layer thicknesses in the ensemble.

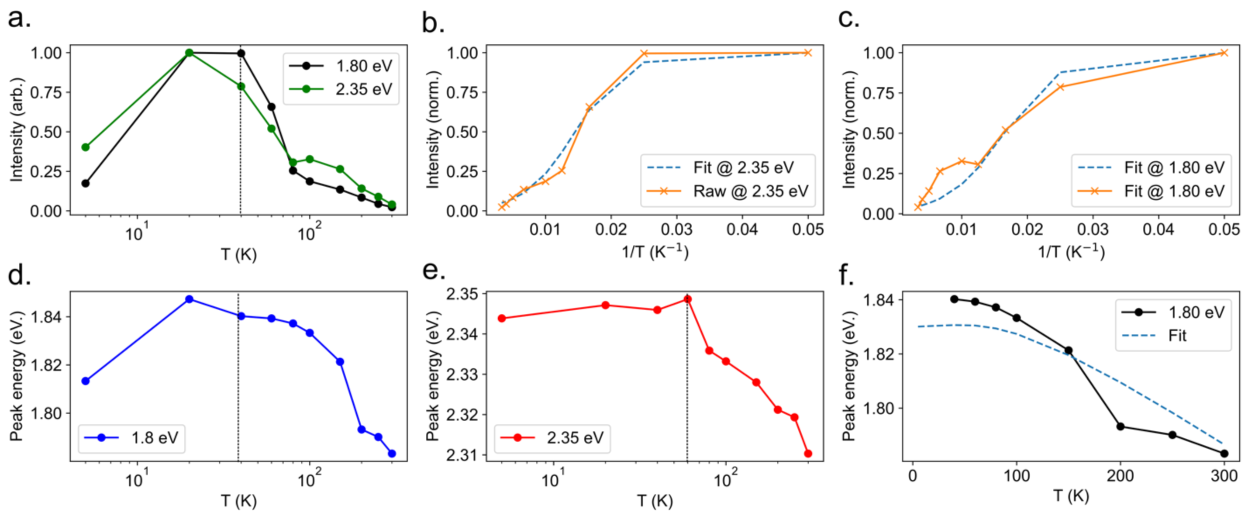

Figure S66 Evolution of PNR emission intensity with temperature and pump power: a. Normalised integrated intensity of PL peaks at 1.80 eV and 2.35 eV as a function of temperature (T). At around 50 K the PL intensity begins to show a non-monotonic variation characteristic of the phase transition that is observed in other measurements around this temperature e.g., UV-Vis and EPR. After this temperature there is a plateau and then drop in the PL intensity, which drops further with increased cooling. This phase transition temperature is marked with black line (also in plots d and e). **b-c.** Fit of PL intensity versus temperature for 2.35 eV and 1.80 eV peaks (up to phase transition) to **Equation 8** described below. **d-e.** Peak energy as a function of temperature for PL peaks at 1.80 eV and 2.35 eV. There is a small red shift in the emission up to ~50 K, before the PL peak begins to blue shift. **f.** Fit of PL peak energy to **Equation 7** described in text as a function of temperature (up to the phase transition).

To investigate the spectra above further, we perform temperature dependent PL measurements. As the temperature is dropped from 200 K to 50 K there is a small red shift of the peaks at 1.8 and 2.35 eV (~40 and ~20 meV, respectively, see **Figure S66**). There is an absolute increase in the intensity of both peaks up to around 50 K, but the overall ratio of intensities between the high and low energy peaks remains approximately constant with decreasing temperature and is independent of the sample location. At ~50 K the PL intensity begins to drop, and PL peaks begin to blue shift. This temperature is consistent with other measurements such as Raman spectroscopy, EPR, UV-Vis and SQUID that suggest a phase transition is occurring around this temperature. We note that because the intensity/position of the various PL peaks does not plateau at the same temperature or show an abrupt change in behaviour we can only approximate the phase transition temperature from these measurements (within the sampling limitations of the measurements also) to somewhere between 45 and 60 K.

Turning to the PL intensity dependence with temperature, the integrated intensity of PNRs shows a T^{-1} dependence (like other semiconductors) because of thermal activation of nonradiative recombination centres as the temperature is raised. To quantify this, the normalised intensity as a function of temperature can be fit with an equation of the form $\frac{1}{(1+ae^{\frac{-E_A}{kT}})}$ (**Equation 8**) where a is related to the ratio of the radiative and non-radiative lifetimes and E_a is the activation energy to escape nonradiative recombination centres. Applying this model to the data in **Figure S66a** and from our fits we find values of 12 ± 3 for a and 21 ± 5 meV for E_a (near identical values for both fitting parameters across both PL transitions). Comparing E_a to the (theoretical) exciton binding energy of PNRs (~1 eV (depending on thickness)[74]), shows that the latter is much higher, supporting the notion that the temperature dependent PL intensity is indeed due to the thermal occupation of nonradiative recombination centres and not exciton dissociation.

The variation in emission peak positions with temperature are harder to ascribe. Generally, the bandgap (and emission) of bulk semiconductors is controlled by a combination of the electron-phonon interactions (which result in renormalisation of the bandgap) and thermal expansion/contraction of the lattice. It is the interplay of these two effects that will determine the peak positions as a function of temperature. Indeed, as we observe **Figure S63** for the pressure dependent absorption, tensile strain/stress of the phosphorene lattice results in an increase in the bandgap (under contraction) i.e., cooling would structurally be expected to drive blue shift the band edge emission. To quantify the above effects, we can fit the small temperature evolution of the band-edge emission to an equation of the form $E = [a'\theta/(e^{\frac{\theta}{T}} - 1)] + mT + c$ (**Equation 7**). Where mT is a linear term capturing the evolution of the band gap due to thermal expansion, a is a constant, c is a parameter related to the band gap at T = 0 K (but can be removed by normalising the data) and θ is the effective frequency for the dominant electron-phonon coupling mode[49]. The assumption we apply when using this model here is that that the emission energy of the low energy band will reflect the evolution of the band gap with temperature and that only a single phonon mode is involved in the carrier relaxation. Using this model we find that a' = +0.003 meV/K , θ = 317 K and m = -0.012 meV/K. Interestingly the θ value we obtain is similar to the B_{1g} edge phonon mode frequency suggesting this mode may play the key role in the electron-phonon interactions in the excited state, further bolstering our claims of a strong coupling of the excited state to an edge phonon.

Finally, we note that PNRs have an underlying anisotropy in their lattice such that we might expect an anisotropic emission response[75]. But this is only expected at the single PNR level and as we measure ensemble films. We hence find the polarisation dependence of the emission signal to be convoluted with the film inhomogeneity and do not explore the polarisation dependence (in either excitation or detection) of the emission.

Transient Temperature Dependent PL

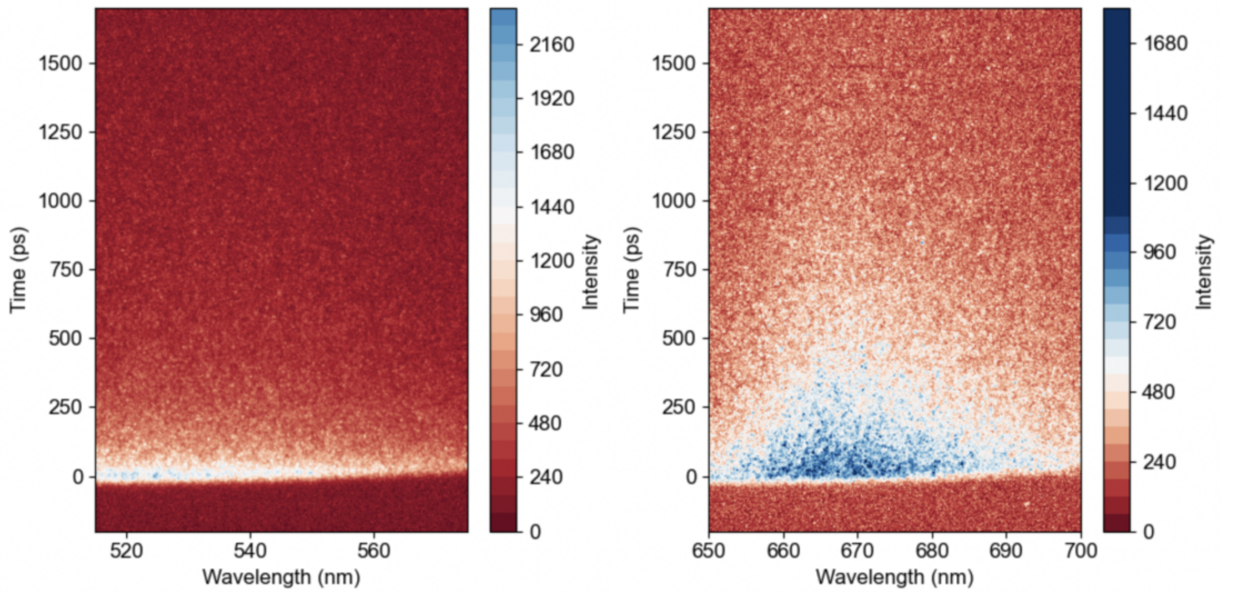

Figure S67 Time resolved emission from PNR film: a-b. Transient (streak camera) emission spectra of a PNR film at room temperature examining low energy (650-700 nm) and high energy (500-600 nm) PL bands.

Having characterised the steady-state PL we perform temperature dependent transient photoluminescence measurements using a streak camera (transient spectra are taken in multiple intervals due to the limited spectral bandwidth of the detector). The transient emission spectra show a broad asymmetric shape, resembling the steady-state PL of PNRs. The transient emission spectrum can be broadly characterised as having a longer-lived band at 1.80 eV and a shorter-lived tail around 2.35 eV. The higher energy band is best fit with a bi-exponential decay of 681 ± 10 ps and 78 ± 10 ps at 200 K, with the lower energy band having a decay of 410 ± 30 ps (fitting, measurement and repeats uncertainty included in error). Interestingly, as shown in **Figure 67b** there is a clear rise to this lower-energy band's signal. While this is too close to the instrument response (22 ps) to accurately resolve, this rise alongside the bi-exponential decay of the high-energy band is in-line with the dipolar relaxation between states we observe in our transient absorption spectra.

Interestingly, there is no detectable change in the emission lifetimes with temperature. Examining the non-normalised absorption spectra of PNRs with temperature in **Figure S39** shows at the laser excitation wavelength of 416 nm there is negligible change in the absorption on cooling (changes are around the band edge). This means that the ~2.3× brightening in the PL spectra in **Figure S66** on cooling from 300 to 50K corresponds to an approximate absolute increase in the PL quantum yield (PLQY). Integrating sphere measurements allow us to measure the room temperature PLQY of PNRs to be 11% ± 5%. This means that the emission yield at 50 K is around 25% ± 7% (accounting for uncertainty in the temperature measurement). The PLQY $= \frac{k_r}{k_r+k_{nr}} = k_r * \tau_e$, where τ_e is the emission lifetime. A constant τ_e with increasing PLQY implies that k_r must increase, whilst k_{nr} drops. In **Figure S68** we show the quantitative evolution of PLQY, τ_e, k_r and k_{nr} with temperature to illustrate this. Because the PLQY is measured across the entire spectrum, and the emission decay has two components with different lifetimes, we take as a first approximation τ_e as simply being the weighted sum of the fitted lifetimes for the emission decay times as above, i.e., we average the lifetime across all spectral bands. From the temperature dependence of the intensity of the emission spectra as we discuss above (see **Equation 8**) we estimated that the k_r/k_{nr} ~ 12. Given that τ_e remains constant, this ratio should also remain constant with temperature, i.e., k_{nr} must drop by the same amount k_r increases. We can use this ratio and estimate k_{nr} as a function of temperature as shown in **Figure S67c**.

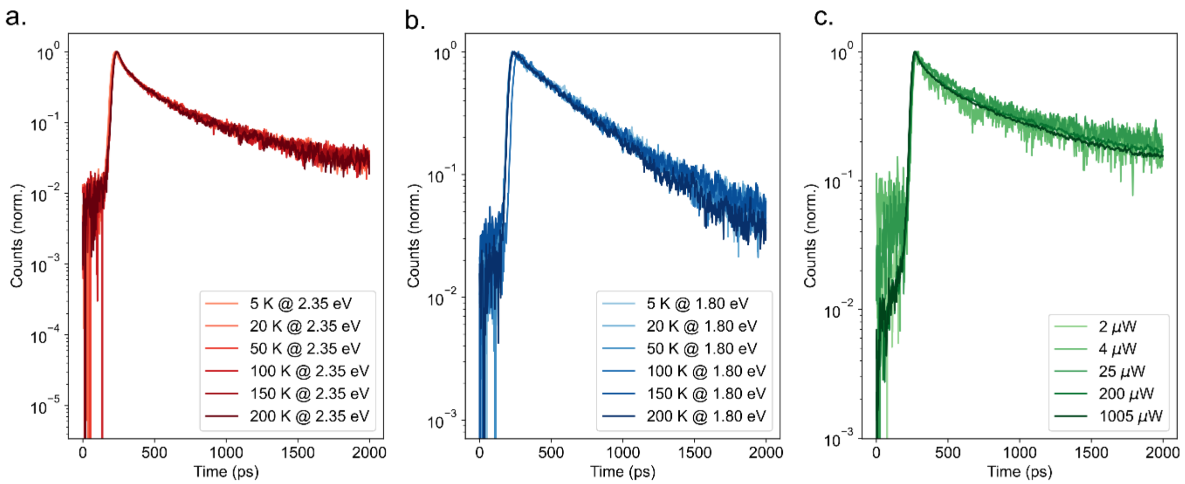

Figure S68 Time resolved emission kinetics from PNR thin film as a function of temperature: a-b. Kinetics of decay from 2.35 eV and 1.80 eV bands as a function of temperature. **c.** Decay from 2.35 eV as a function of laser power. Limited variation is observed with both temperature and power.

We note there is no fluence dependence to τ_e within the explored power range. The former indicates a low Auger coefficient for PNRs and limited annihilation effects, in-keeping with observations also made for ML bP[70]. An assumption of the above fits is that the emission from the two PL bands originates from a similar state.

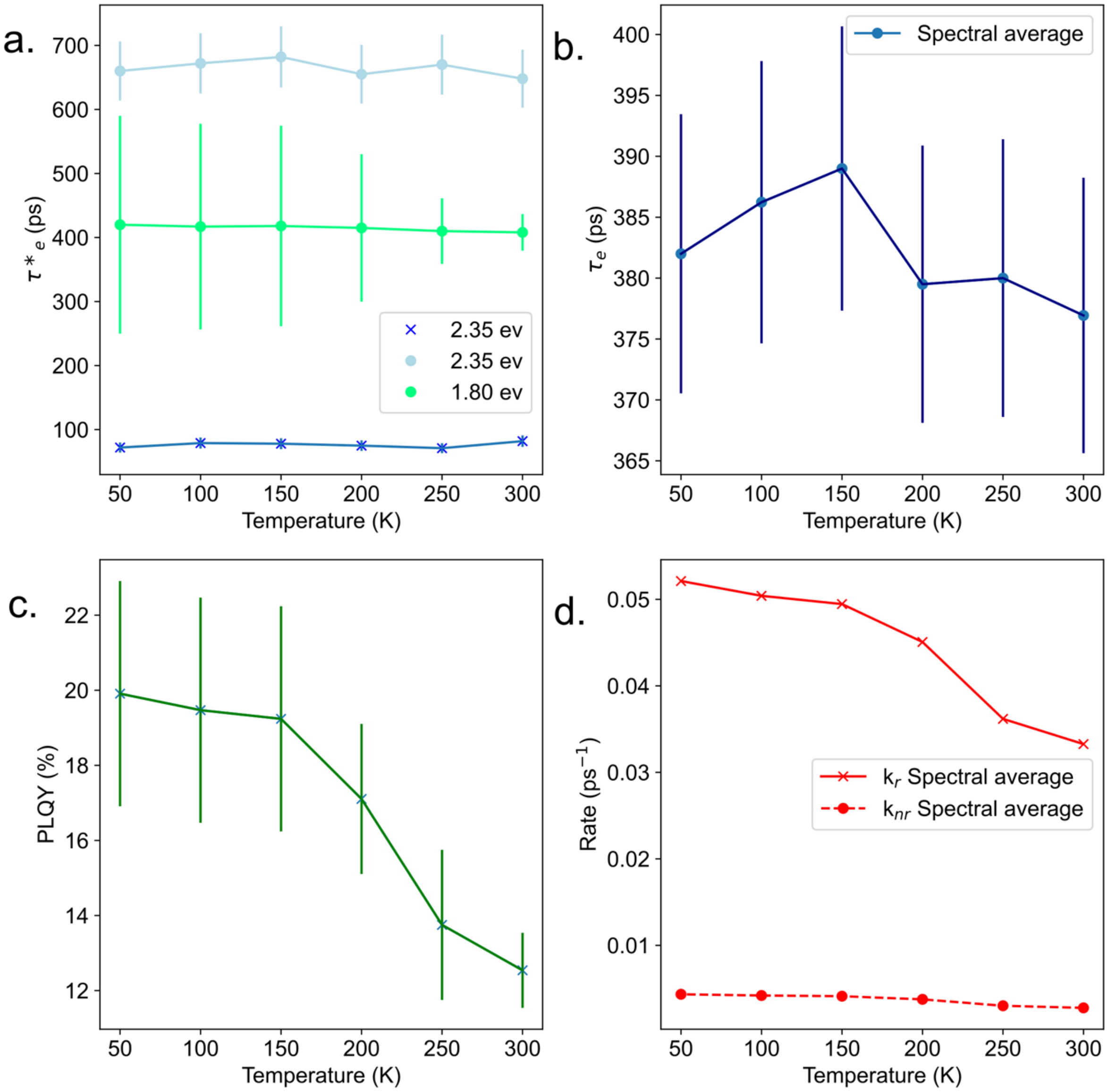

Figure S69 Evolution of PNR emission lifetime, radiative/non-radiative rates and quantum yields as a function of temperature: a. Emission lifetime τ^*_e as a function of temperature for 2.35 eV and 1.80 eV emission bands. The high energy band has two decay components. Error bars capture fitting and measurement error. Instrument response function is 22 ps. **b.** Emission lifetime τ_e following a weighted average across the different decay bands. **c.** PLQY as a function of temperature for PNRs. **d.** Radiative (k_r) and non-radiative (k_{nr}) decay rate of PNRs averaging across emission bands.

Supplementary Note 21: Scaling of pump-probe signal with magnetic field

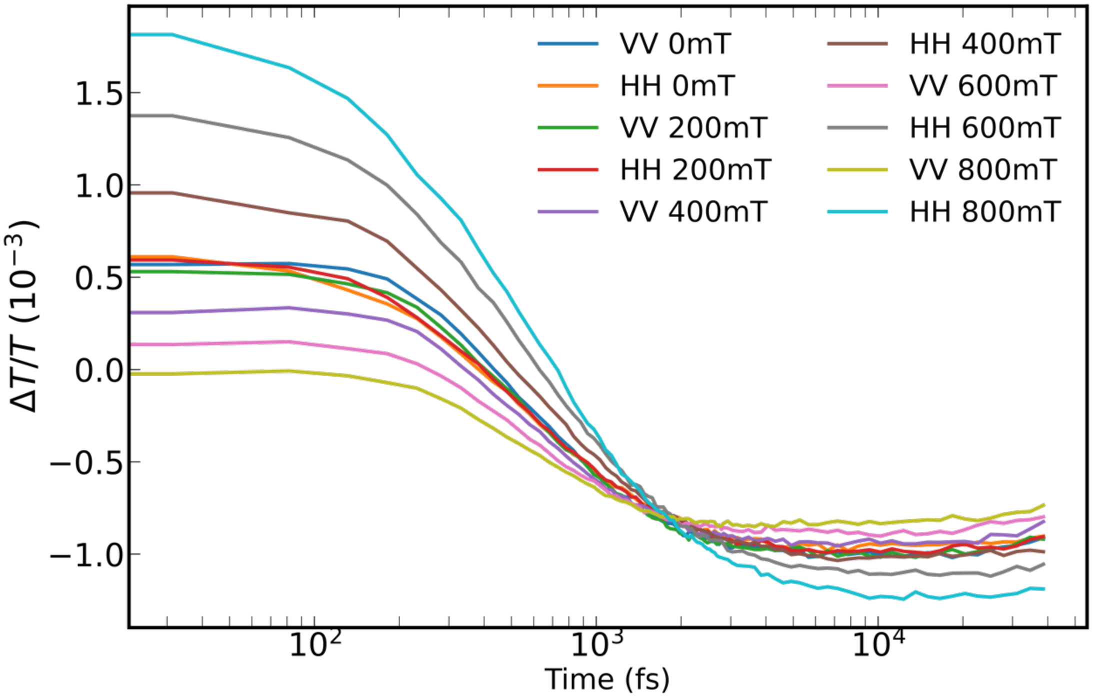

Figure S70 Magnetic field dependence of the pump-probe signal: At intermediate fields from those shown in the main text (200, 400 and 600 mT), the splitting between the HH and VV signal linearly increases as would be expected based on the linear dichroism results shown in **Figure 1b** of the main text.

Supplementary Notes 22: Electroabsorption Measurements

To extract more quantitative information about the electronic states involved in the optical absorption, we perform electroabsorption (EA) measurements. Here, a sandwich device structure is fabricated consisting of (in the following order) Au/Cr (30 nm), Al_2O_3 (150 nm), PNRs (20 nm), Al_2O_3 (150 nm) and ITO/glass (substrate); **Figure S71a**. An alternating voltage (up to 100 V peak-to-peak) and hence field (up to 1 MV/cm) is applied across the device. A monochromated light source passing through the device onto a photodiode is used to measure the resulting change in transmission in the presence of a field. By examining the influence of an electric field on the absorption properties of the material we can extract more clearly transitions in the absorption spectrum of the material and quantitative information such as the difference in ground and excited state dipole moments (Δμ) and polarizability (Δp). **Figure S71b** shows the electroabsorption spectrum of PNRs at 1 MV/cm. The spectrum consists of two derivative-like transitions, one at ~410 nm and another at ~590 nm. The former is of high-intensity and narrow, whilst the latter is broad and of low intensity. Theoretically, the change in absorption of a material is a sum of the zeroth, first and second derivative of the materials absorption spectrum as can be summarised by the following equation:

$$\Delta A(v) = (fF)^2 \left[A_X A(v) + B_X v \frac{d}{dv}\left(\frac{A(v)}{v}\right) + C_X v \frac{d^2}{dv^2}\left(\frac{A(v)}{v}\right)\right],$$ (**Equation 9**)

where f is the internal field factor, F the field magnitude, X polarization direction of electric vector of the absorption light and A_X is a constant. The other terms are defined as follows:

$$B_X = \frac{\Delta p}{2hc}$$ (**Equation 10**)

$$C_X = \frac{\Delta \mu^2}{6h^2c^2}$$ (**Equation 11**)

Where Δp and $\Delta \mu$ are differences in polarisablity and dipole moment between the ground and excited state. This so-called differential method is useful to analyse EA spectra when the absorption peaks are strong and well-separated but for materials like PNRs where the absorption is weak and broad (and the first and second derivatives of the absorption do not strongly capture the EA as shown in **Figure S71c, d**), an integral method is instead preferred. Here, the first derivative term in the above expansion can be represented by the first integral of the EA spectrum whilst the second derivative term in the above equation can be taken from the integral of the integrated EA spectrum (second integral; see refs[76–78] for details). Performing the above analysis, we find that for the ~410 nm transition Δp ~0.3 Å^3 and Δμ ~10D. For the 590 nm transition Δp ~0.9 Å^3 and Δμ ~19D. We note that as we are performing EA on an ensemble of PNRs our values are naturally inhomogenously broadened and we yield an average dipole moment and polarizability change.

The above analysis firstly reveals the presence of two distinct transition within the broad absorption spectrum of PNRs. The transition at lower energies involves excited states with a greater charge-separated character compared to those at higher-energy. Furthermore, the linear scaling of the area under in the EA spectrum with field (**Figure S71e, f**) indicates a built-in field or permanent dipole moments to the system. This is in-keeping with the large negative zeta potential of PNRs in solution at ~-47 mV and the presence of dangling bond/electron rich edges.

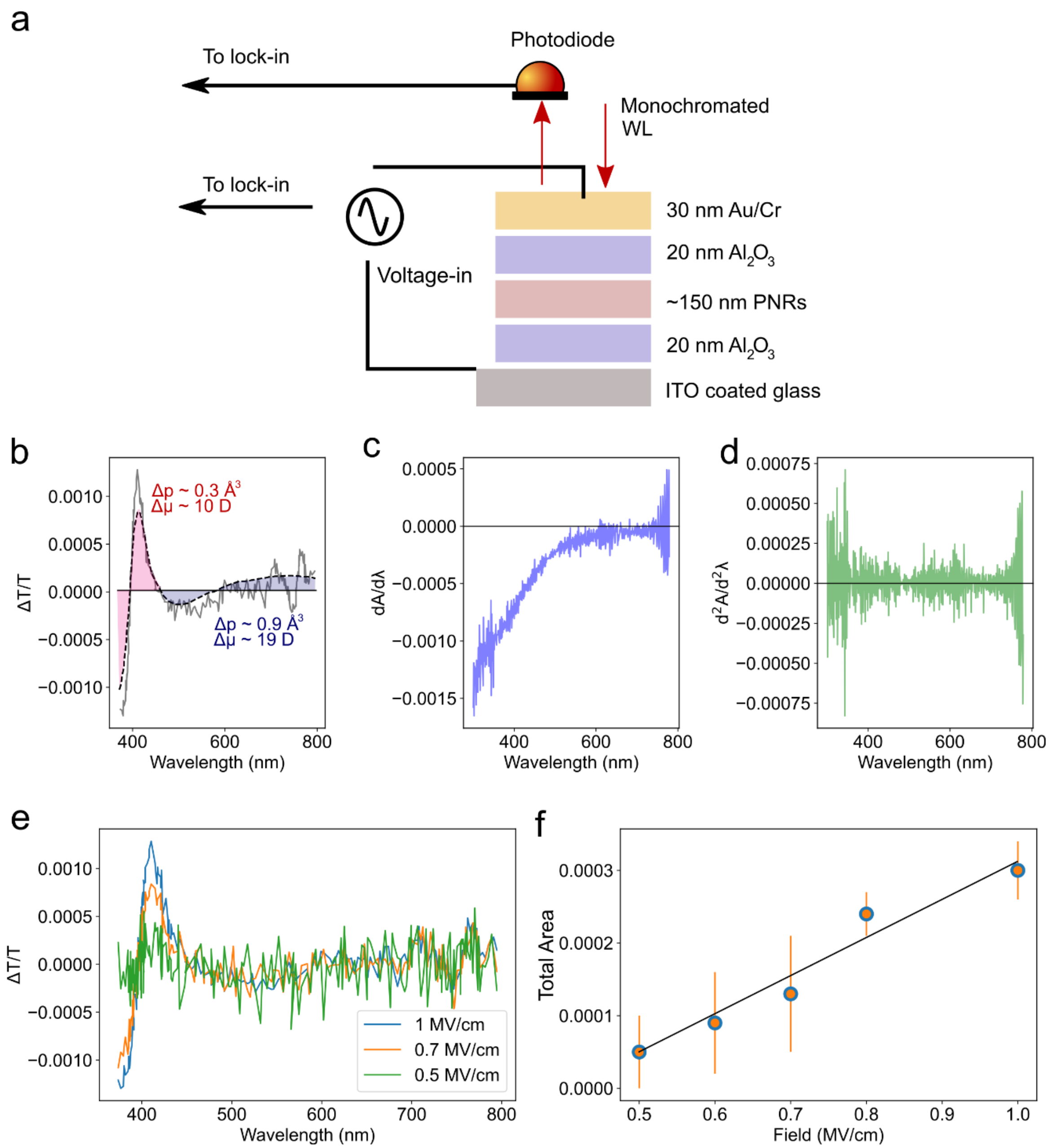

Figure S72 Electroabsorption spectroscopy of PNRs: a. Cartoon schematic of device structure and optical setup used to measure electro-absorption spectra. **b.** Electro-absorption spectrum (at 1 MV/cm field) of a PNR film and associated integral fits shown in red and blue shading. The difference in dipole moment and polarizability between the ground and excited state are shown as insets. **c.** First derivative of absorption spectra of PNRs. **d.** Second derivative of absorption spectra of PNRs. **e.** Electroabsorption spectrum as a function of field strength**. f.** Total integrated area of electro-absorption spectrum as a function of field for PNRs.

Supplementary Note 23: Additional Impulsive Vibrational Spectra

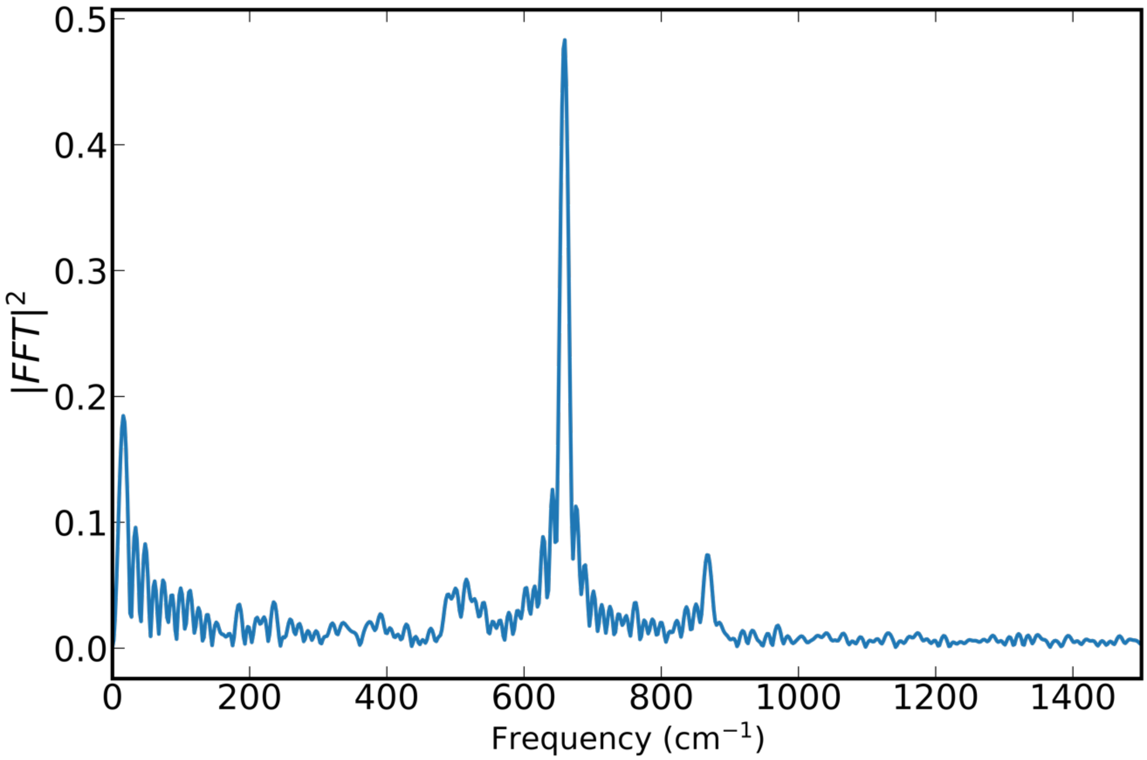

Figure S73 Off-resonant Impulsive Vibrational Spectroscopy of PNRs in DMF: The off-resonant broadband pump pulse on the PNR solution from 700-800 nm does not show the edge phonon mode coupling as compared to the resonant pulse (500-600 nm) shown in the main text **Figure 4**. This is further evidence for the coupling of the edge phonon to the excited state of the PNRs.

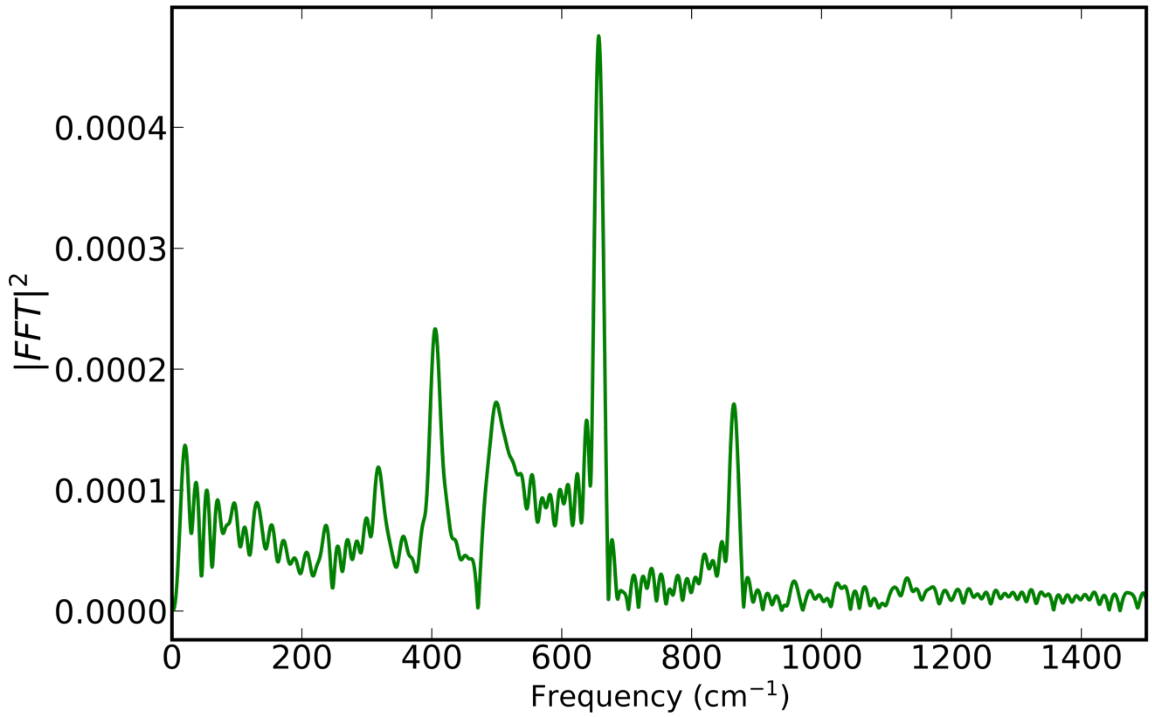

Figure S74 Impulsive Vibrational Spectroscopy of pure DMF: The same resonant broadband pump pulse used on the PNR solution (500-600 nm) is used to study a pure DMF solution to ensure the phonon mode we ascribe to the PNR edge is not arising from the solvent. As can be seen, this mode is not present in the pure DMF solvent. This confirms that is indeed arising from the PNRs.

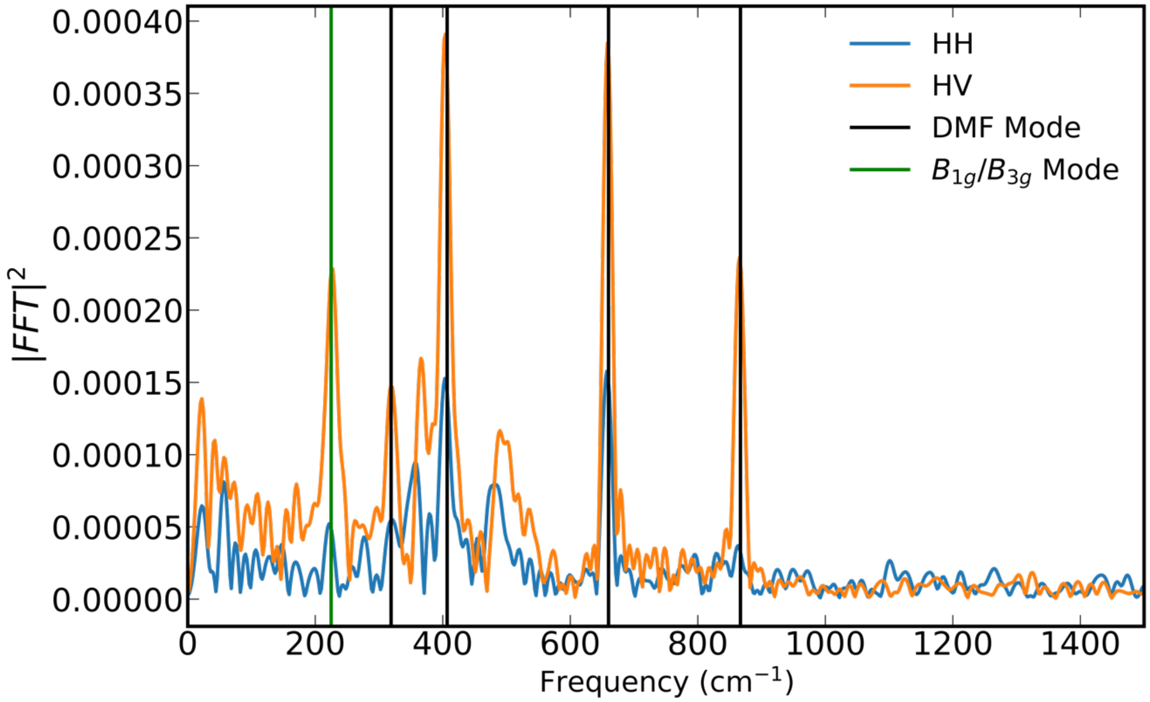

Figure S75 Polarization Dependent Impulsive Vibrational Spectroscopy of the edge mode: The edge phonon mode (green line) is only present when studying the impulsive vibrational spectrum in the orthogonally polarized pump-probe configuration suggesting that the vibration selectively couples to the diploe polarized along the ribbon's long axis. The modes of the DMF solvent are marked with black lines. There is an A_g^1 mode of PNRs at ~360 cm^{-1} which is only (/significantly more strongly) observed in the HV pump and probe polarization configuration. As suggested by Ribiero *et al*.[23] this can be taken as tentative evidence of zigzag terminated edges in PNRs.

Supplementary Note 24: SQUID Sequences

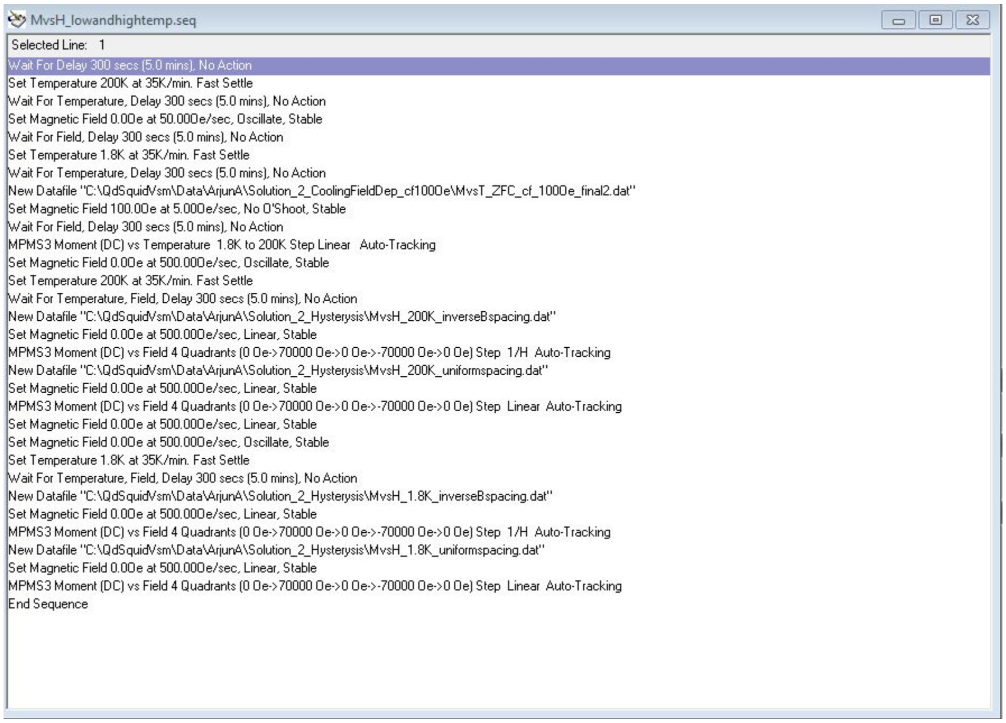

```
MvsH_lowandhightemp.seq
Selected Line:   1
Wait For Delay 300 secs (5.0 mins), No Action
Set Temperature 200K at 35K/min. Fast Settle
Wait For Temperature, Delay 300 secs (5.0 mins), No Action
Set Magnetic Field 0.0Oe at 50.000Oe/sec, Oscillate, Stable
Wait For Field, Delay 300 secs (5.0 mins), No Action
Set Temperature 1.8K at 35K/min. Fast Settle
Wait For Temperature, Delay 300 secs (5.0 mins), No Action
New Datafile "C:\QdSquidVsm\Data\ArjunA\Solution_2_CoolingFieldDep_cf100Oe\MvsT_ZFC_cf_100Oe_final2.dat"
Set Magnetic Field 100.0Oe at 5.000Oe/sec, No O'Shoot, Stable
Wait For Field, Delay 300 secs (5.0 mins), No Action
MPMS3 Moment (DC) vs Temperature  1.8K to 200K Step Linear   Auto-Tracking
Set Magnetic Field 0.0Oe at 500.000Oe/sec, Oscillate, Stable
Set Temperature 200K at 35K/min. Fast Settle
Wait For Temperature, Field, Delay 300 secs (5.0 mins), No Action
New Datafile "C:\QdSquidVsm\Data\ArjunA\Solution_2_Hysterysis\MvsH_200K_inverseBspacing.dat"
Set Magnetic Field 0.0Oe at 500.000Oe/sec, Linear, Stable
MPMS3 Moment (DC) vs Field 4 Quadrants (0 Oe->70000 Oe->0 Oe->-70000 Oe->0 Oe) Step  1/H  Auto-Tracking
New Datafile "C:\QdSquidVsm\Data\ArjunA\Solution_2_Hysterysis\MvsH_200K_uniformspacing.dat"
Set Magnetic Field 0.0Oe at 500.000Oe/sec, Linear, Stable
MPMS3 Moment (DC) vs Field 4 Quadrants (0 Oe->70000 Oe->0 Oe->-70000 Oe->0 Oe) Step  Linear  Auto-Tracking
Set Magnetic Field 0.0Oe at 500.000Oe/sec, Linear, Stable
Set Magnetic Field 0.0Oe at 500.000Oe/sec, Oscillate, Stable
Set Temperature 1.8K at 35K/min. Fast Settle
Wait For Temperature, Field, Delay 300 secs (5.0 mins), No Action
New Datafile "C:\QdSquidVsm\Data\ArjunA\Solution_2_Hysterysis\MvsH_1.8K_inverseBspacing.dat"
Set Magnetic Field 0.0Oe at 500.000Oe/sec, Linear, Stable
MPMS3 Moment (DC) vs Field 4 Quadrants (0 Oe->70000 Oe->0 Oe->-70000 Oe->0 Oe) Step  1/H  Auto-Tracking
New Datafile "C:\QdSquidVsm\Data\ArjunA\Solution_2_Hysterysis\MvsH_1.8K_uniformspacing.dat"
Set Magnetic Field 0.0Oe at 500.000Oe/sec, Linear, Stable
MPMS3 Moment (DC) vs Field 4 Quadrants (0 Oe->70000 Oe->0 Oe->-70000 Oe->0 Oe) Step  Linear  Auto-Tracking
End Sequence
```

Figure S76 Original SQUID sequence files for measurements of the PNRs deposited straight into a plastic straw.

```
20230424_MvsT_ZFCandFC_MvH_300K_1p8K.seq
Selected Line: 5
Set Temperature 300K at 35K/min. Fast Settle
Wait For Temperature, Delay 600 secs (10.0 mins), No Action
Set Magnetic Field 70000.0Oe at 500.00Oe/sec, Linear, Stable
Wait For Field, Delay 600 secs (10.0 mins), No Action
Set Magnetic Field 0.0Oe at 500.00Oe/sec, Oscillate, Stable
Wait For Field, Delay 600 secs (10.0 mins), No Action
Magnet Reset
Wait For Temperature, Field, Delay 1200 secs (20.0 mins), No Action

Set Temperature 350K at 35K/min. Fast Settle
Wait For Temperature, Delay 1200 secs (20.0 mins), No Action
Set Temperature 1.8K at 35K/min. Fast Settle
Wait For Temperature, Delay 1200 secs (20.0 mins), No Action
New Datafile "C:\QdSquidVsm\Data\ArjunA\Review_07052023\Bg\20230507_MvT_ZFC_500Oe_warmup.dat"
Set Magnetic Field 500.0Oe at 50.00Oe/sec, No O'Shoot, Stable
Wait For Field, Delay 600 secs (10.0 mins), No Action
MPMS3 Moment (DC) vs Temperature  1.8K to 350K Step Linear   Auto-Tracking
Wait For Delay 120 secs (2.0 mins), No Action

Wait For Temperature, Delay 1200 secs (20.0 mins), No Action
Set Magnetic Field 50000.0Oe at 500.00Oe/sec, Linear, Stable
Wait For Field, Delay 600 secs (10.0 mins), No Action
Set Temperature 1.8K at 35K/min. Fast Settle
Wait For Temperature, Delay 1200 secs (20.0 mins), No Action
New Datafile "C:\QdSquidVsm\Data\ArjunA\Review_07052023\Bg\20230507_MvT_FC5T_500Oe_warmup.dat"
Set Magnetic Field 500.0Oe at 50.00Oe/sec, No O'Shoot, Stable
Wait For Field, Delay 600 secs (10.0 mins), No Action
MPMS3 Moment (DC) vs Temperature  1.8K to 350K Step Linear   Auto-Tracking
Wait For Delay 120 secs (2.0 mins), No Action
Wait For Temperature, Delay 600 secs (10.0 mins), No Action

Set Temperature 300K at 35K/min. Fast Settle
Wait For Temperature, Delay 600 secs (10.0 mins), No Action
Set Magnetic Field 70000.0Oe at 500.00Oe/sec, Linear, Stable
Wait For Field, Delay 600 secs (10.0 mins), No Action
Set Magnetic Field 0.0Oe at 500.00Oe/sec, Oscillate, Stable
Wait For Field, Delay 600 secs (10.0 mins), No Action
Magnet Reset
Wait For Temperature, Field, Delay 1200 secs (20.0 mins), No Action

New Datafile "C:\QdSquidVsm\Data\ArjunA\Review_07052023\Bg\20230507_MvH_300K.dat"
MPMS3 Moment (DC) vs Field 4 Quadrants (0 Oe->70000 Oe->0 Oe->-70000 Oe->0 Oe) Step  Linear  Auto-Tracking
Wait For Delay 120 secs (2.0 mins), No Action

Set Magnetic Field 70000.0Oe at 500.00Oe/sec, Linear, Stable
Wait For Field, Delay 600 secs (10.0 mins), No Action
Set Magnetic Field 0.0Oe at 500.00Oe/sec, Oscillate, Stable
Wait For Field, Delay 600 secs (10.0 mins), No Action
Magnet Reset
```

```
Magnet Reset
Wait For Temperature, Field, Delay 1200 secs (20.0 mins), No Action
Set Temperature 1.8K at 35K/min. Fast Settle
Wait For Temperature, Delay 1200 secs (20.0 mins), No Action

New Datafile "C:\QdSquidVsm\Data\ArjunA\Review_07052023\Bg\20230507_MvH_1p8K.dat"
MPMS3 Moment (DC) vs Field 4 Quadrants (0 Oe->70000 Oe->0 Oe->-70000 Oe->0 Oe) Step  Linear  Auto-Tracking
Wait For Delay 120 secs (2.0 mins), No Action

Set Temperature 300K at 35K/min. Fast Settle
End Sequence
```

Figure S77 SQUID sequence files for measurements of the PNRs deposited on a quartz rod at room temperature: Here, SQUID measurements involve background subtraction post measurement.

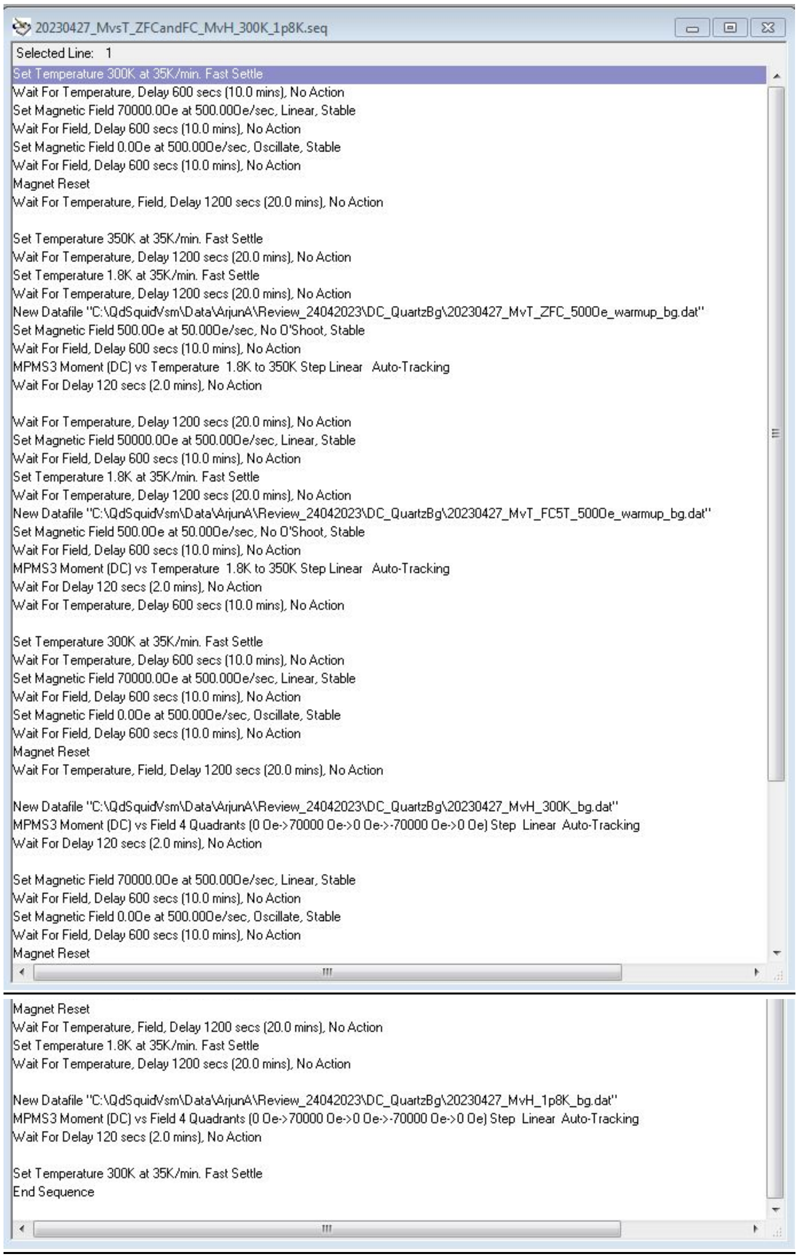

20230427_MvsT_ZFCandFC_MvH_300K_1p8K.seq

Selected Line: 1

Set Temperature 300K at 35K/min. Fast Settle
Wait For Temperature, Delay 600 secs (10.0 mins), No Action
Set Magnetic Field 70000.0Oe at 500.00Oe/sec, Linear, Stable
Wait For Field, Delay 600 secs (10.0 mins), No Action
Set Magnetic Field 0.0Oe at 500.00Oe/sec, Oscillate, Stable
Wait For Field, Delay 600 secs (10.0 mins), No Action
Magnet Reset
Wait For Temperature, Field, Delay 1200 secs (20.0 mins), No Action

Set Temperature 350K at 35K/min. Fast Settle
Wait For Temperature, Delay 1200 secs (20.0 mins), No Action
Set Temperature 1.8K at 35K/min. Fast Settle
Wait For Temperature, Delay 1200 secs (20.0 mins), No Action
New Datafile "C:\QdSquidVsm\Data\ArjunA\Review_24042023\DC_QuartzBg\20230427_MvT_ZFC_500Oe_warmup_bg.dat"
Set Magnetic Field 500.0Oe at 50.00Oe/sec, No O'Shoot, Stable
Wait For Field, Delay 600 secs (10.0 mins), No Action
MPMS3 Moment (DC) vs Temperature 1.8K to 350K Step Linear Auto-Tracking
Wait For Delay 120 secs (2.0 mins), No Action

Wait For Temperature, Delay 1200 secs (20.0 mins), No Action
Set Magnetic Field 50000.0Oe at 500.00Oe/sec, Linear, Stable
Wait For Field, Delay 600 secs (10.0 mins), No Action
Set Temperature 1.8K at 35K/min. Fast Settle
Wait For Temperature, Delay 1200 secs (20.0 mins), No Action
New Datafile "C:\QdSquidVsm\Data\ArjunA\Review_24042023\DC_QuartzBg\20230427_MvT_FC5T_500Oe_warmup_bg.dat"
Set Magnetic Field 500.0Oe at 50.00Oe/sec, No O'Shoot, Stable
Wait For Field, Delay 600 secs (10.0 mins), No Action
MPMS3 Moment (DC) vs Temperature 1.8K to 350K Step Linear Auto-Tracking
Wait For Delay 120 secs (2.0 mins), No Action
Wait For Temperature, Delay 600 secs (10.0 mins), No Action

Set Temperature 300K at 35K/min. Fast Settle
Wait For Temperature, Delay 600 secs (10.0 mins), No Action
Set Magnetic Field 70000.0Oe at 500.00Oe/sec, Linear, Stable
Wait For Field, Delay 600 secs (10.0 mins), No Action
Set Magnetic Field 0.0Oe at 500.00Oe/sec, Oscillate, Stable
Wait For Field, Delay 600 secs (10.0 mins), No Action
Magnet Reset
Wait For Temperature, Field, Delay 1200 secs (20.0 mins), No Action

New Datafile "C:\QdSquidVsm\Data\ArjunA\Review_24042023\DC_QuartzBg\20230427_MvH_300K_bg.dat"
MPMS3 Moment (DC) vs Field 4 Quadrants (0 Oe->70000 Oe->0 Oe->-70000 Oe->0 Oe) Step Linear Auto-Tracking
Wait For Delay 120 secs (2.0 mins), No Action

Set Magnetic Field 70000.0Oe at 500.00Oe/sec, Linear, Stable
Wait For Field, Delay 600 secs (10.0 mins), No Action
Set Magnetic Field 0.0Oe at 500.00Oe/sec, Oscillate, Stable
Wait For Field, Delay 600 secs (10.0 mins), No Action
Magnet Reset

Magnet Reset
Wait For Temperature, Field, Delay 1200 secs (20.0 mins), No Action
Set Temperature 1.8K at 35K/min. Fast Settle
Wait For Temperature, Delay 1200 secs (20.0 mins), No Action

New Datafile "C:\QdSquidVsm\Data\ArjunA\Review_24042023\DC_QuartzBg\20230427_MvH_1p8K_bg.dat"
MPMS3 Moment (DC) vs Field 4 Quadrants (0 Oe->70000 Oe->0 Oe->-70000 Oe->0 Oe) Step Linear Auto-Tracking
Wait For Delay 120 secs (2.0 mins), No Action

Set Temperature 300K at 35K/min. Fast Settle
End Sequence

Figure S78 SQUID sequence files for measurements of the PNRs deposited on a quartz rod at 1.8 K: Here, SQUID measurements involve background subtraction post measurement.

References

1. Watts, M. C. Production and characterisation of phosphorene nanoribbons. *Doctoral thesis, UCL (University College London).* (2020).
2. Watts, M. C. *et al.* Production of phosphorene nanoribbons. *Nature* **568**, 216–220 (2019).
3. Nielsen, F. On a Variational Definition for the Jensen-Shannon Symmetrization of Distances Based on the Information Radius. *Entropy* **23**, 464 (2021).
4. Poljak, M. & Suligoj, T. Immunity of electronic and transport properties of phosphorene nanoribbons to edge defects. *Nano Res* **9**, 1723–1734 (2016).
5. Wang, H. *et al.* Graphene nanoribbons for quantum electronics. *Nat Rev Phys* **3**, 791–802 (2021).
6. Lyu, B. *et al.* Graphene nanoribbons grown in hBN stacks for high-performance electronics. *Nature* **628**, 758–764 (2024).
7. Zhang, J. *et al.* Phosphorene nanoribbon as a promising candidate for thermoelectric applications. *Sci Rep* **4**, 1–8 (2014).
8. Barone, V., Hod, O. & Scuseria, G. E. Electronic structure and stability of semiconducting graphene nanoribbons. *Nano Lett* **6**, 2748–2754 (2006).
9. Wu, Q. *et al.* Electronic and transport properties of phosphorene nanoribbons. *Phys Rev B* **92**, 1–9 (2015).
10. Nourbakhsh, Z. & Asgari, R. Charge transport in doped zigzag phosphorene nanoribbons. *Phys Rev B* **97**, 235406 (2018).
11. Corona-García, C. A., Martínez-Olguín, A. C. & Cocoletzi, G. H. Transport properties of blue phosphorene nanoribbons in the presence of pollutant molecules. *Micro and Nanostruct* **167**, 207257 (2022).
12. Jin, J. *et al.* The electronic and transport properties of the phosphorene nanoribbons. *Mater Res Express* **6**, 096317 (2019).
13. MacDonald, T. J. *et al.* Phosphorene Nanoribbon-Augmented Optoelectronics for Enhanced Hole Extraction. *J Am Chem Soc* **143**, 21549–21559 (2021).
14. Wu, W., Zhang, Z., Lu, P. & Guo, W. Electronic and magnetic properties of zigzag graphene nanoribbons with periodic protruded edges. *Phys Rev B* **82**, 085425 (2010).
15. Rizzo, D. J. *et al.* Inducing metallicity in graphene nanoribbons via zero-mode superlattices. *Science* 6511 **369**, (2020).
16. Kulish, V. V., Malyi, O. I., Persson, C. & Wu, P. Phosphorene as an anode material for Na-ion batteries: a first-principles study. *Phys Chem Chem Phys* **17**, 13921–13928 (2015).
17. Yu, W. *et al.* Facile Production of Phosphorene Nanoribbons towards Application in Lithium Metal Battery. *Advanced Materials* **33**, 2102083 (2021).
18. De Abajo, F. J. G. Graphene plasmonics: Challenges and opportunities. *ACS Photonics* **1**, 133–152 (2014).
19. Hu, W., Lin, L., Zhang, R., Yang, C. & Yang, J. Highly Efficient Photocatalytic Water Splitting over Edge-Modified Phosphorene Nanoribbons. *J Am Chem Soc* **139**, 15429–15436 (2017).
20. Kim, H. *et al.* Actively variable-spectrum optoelectronics with black phosphorus. *Nature 2021 596:7871* **596**, 232–237 (2021).
21. Abellµn, G. *et al.* Exploring the Formation of Black Phosphorus Intercalation Compounds with Alkali Metals. *Angew Chem Int Ed* **56**, 15267–15273 (2017).

22. Liu, Z. *et al.* Unzipping of black phosphorus to form zigzag-phosphorene nanobelts. *Nat Commun* **11**, 1–10 (2020).
23. Ribeiro, H. B. *et al.* Edge phonons in black phosphorus. *Nat Commun* **7**, 1–7 (2016).
24. Lin, T., Cong, X., Lin, M. L., Liu, X. L. & Tan, P. H. The phonon confinement effect in two-dimensional nanocrystals of black phosphorus with anisotropic phonon dispersions. *Nanoscale* **10**, 8704–8711 (2018).
25. Mao, N. *et al.* Direct Observation of Symmetry-Dependent Electron-Phonon Coupling in Black Phosphorus. *J Am Chem Soc* **141**, 18994–19001 (2019).
26. Vahedi, J. & Peters, R. Edge magnetic properties of black phosphorene nanoribbons. *Phys Rev B* **103**, 75108 (2021).
27. Zhu, Z. *et al.* Magnetism of zigzag edge phosphorene nanoribbons. *Appl Phys Lett* **105**, (2014).
28. Yang, G., Xu, S., Zhang, W., Ma, T. & Wu, C. Room-temperature magnetism on the zigzag edges of phosphorene nanoribbons. *Phys Rev B* **94**, 1–5 (2016).
29. Golor, M., Wessel, S. & Schmidt, M. J. Quantum nature of edge magnetism in graphene. *Phys Rev Lett* **112**, 1–5 (2014).
30. Wang, X. *et al.* Highly anisotropic and robust excitons in monolayer black phosphorus. *Nat Nanotechnol* **10**, 517–521 (2015).
31. Nečas, D., Valtr, M. & Klapetek, P. How levelling and scan line corrections ruin roughness measurement and how to prevent it. *Sci Rep* **10**, 1–15 (2020).
32. Tiwari, A. & Persson, B. N. J. Cylinder-Flat Contact Mechanics with Surface Roughness. *Tribol Lett* **69**, 1–7 (2021).
33. Sumaiya, S. A., Martini, A. & Baykara, M. Z. Improving the reliability of conductive atomic force microscopy-based electrical contact resistance measurements. *Nano Express* **1**, 030023 (2020).
34. Trought, M. & Perrine, K. A. Investigating the relationship between adhesion forces and surface functionalization using atomic force microscopy. *J Chem Educ* **98**, (2021).
35. Liang, L. *et al.* Electronic bandgap and edge reconstruction in phosphorene materials. *Nano Lett* **14**, 6400–6406 (2014).
36. Taghizadeh Sisakht, E., Zare, M. H. & Fazileh, F. Scaling laws of band gaps of phosphorene nanoribbons: A tight-binding calculation. *Phys Rev B* **91**, 085409 (2015).
37. Xia, M. *et al.* Robust charge spatial separation and linearly tunable band gap of low-energy tube-edge phosphorene nanoribbon. *Nanoscale Adv* **3**, 4416–4423 (2021).
38. Shukla, A., Sisodia, M. & Pathak, A. Complete characterization of the directly implementable quantum gates used in the IBM quantum processors. *Phys Lett A* **384**, 126387 (2020).
39. Ci, J.-W., Tu, W.-C., Uen, W.-Y., Khan, I. & Hong, J. Manipulation of magnetic state in phosphorene layer by non-magnetic impurity doping. *New J Phys* **17**, 023056 (2015).
40. Cai, J. *et al.* Atomically precise bottom-up fabrication of graphene nanoribbons. *Nature* **466**, 470–473 (2010).
41. Tao, C. *et al.* Spatially resolving edge states of chiral graphene nanoribbons. *Nat Phys* **7**, 616–620 (2011).
42. Rizzo, D. J. *et al.* Topological band engineering of graphene nanoribbons. *Nature* **560**, 204–208 (2018).
43. Sofer, Z. *et al.* Layered Black Phosphorus: Strongly Anisotropic Magnetic, Electronic, and Electron-Transfer Properties. *Angew Chem Int Ed* **55**, 3382–3386 (2016).

44. Niu, W. *et al.* Exceptionally clean single-electron transistors from solutions of molecular graphene nanoribbons. *Nat Mater* **22**, 180–185 (2023).
45. Hulst, H. C. van de (Hendrik C. Light scattering by small particles. 470 (1981).
46. Coak, M. J. *et al.* SquidLab - A user-friendly program for background subtraction and fitting of magnetization data. *Rev Sci Instr* **91**, 23901 (2020).
47. Hamaguchi, H. O., Tasumi, M., Yoshifuji, M. & Inamoto, N. The P=P Stretching Frequency Observed in the Resonance Raman Spectrum of Bis(2,4,6-tri-tert-butylphenyl)diphosphene. *J Am Chem Soc* **106**, 508–509 (1984).
48. Ozhukil Valappil, M., Ahlawat, M., Pillai, V. K. & Alwarappan, S. A single-step, electrochemical synthesis of nitrogen doped blue luminescent phosphorene quantum dots. *Chem Comm* **54**, 11733–11736 (2018).
49. Huang, S. *et al.* From Anomalous to Normal: Temperature Dependence of the Band Gap in Two-Dimensional Black Phosphorus. *Phys Rev Lett* **125**, 156802 (2020).
50. Wittmann, J. J. *et al.* High-precision measurement of the electron spin g factor of trapped atomic nitrogen in the endohedral fullerene N@C60. *J Mag Res* **290**, 12–17 (2018).
51. Zhou, X. *et al.* Effective g factor in black phosphorus thin films. *Phys Rev B* **95**, 1–8 (2017).
52. Mills, D. L. Surface anisotropy and surface spin canting in the semi-infinite ferromagnet. *Phys Rev B* **39**, 306–307 (1989).
53. Stoll, S. & Schweiger, A. EasySpin, a comprehensive software package for spectral simulation and analysis in EPR. *J Mag Res* **178**, 42–55 (2006).
54. Shukla, V., Kumawat, R. L., Jena, N. K., Pathak, B. & Ahuja, R. Electronic and Transport Properties of Bilayer Phosphorene Nanojunction: Effect of Paired Substitution Doping. *ACS Appl Electron Mater* **3**, 733–742 (2021).
55. Wijeratne, S. S. *et al.* Detecting the Biopolymer Behavior of Graphene Nanoribbons in Aqueous Solution. *Sci Rep* **6**, 1–6 (2016).
56. Appell, J., Porte, G. & Poggi, Y. Quantitative estimate of the orientational persistence length of flexible elongated micelles of cetylpyridinium bromide. *J Colloid Interface Sci* **87**, 492–499 (1982).
57. Maret, G. & Dransfeld, K. Biomolecules and Polymers in High Steady Magnetic Fields. 143–204 (1985) doi:10.1007/3-540-13504-9_10.
58. Hanamura, E., Nagaosa, N., Kumagai, M. & Takagahara, T. Quantum wells with enhanced exciton effects and optical non-linearity. *Mat Sci Eng: B* **1**, 255–258 (1988).
59. Han, X., Morgan Stewart, H., Shevlin, S. A., Catlow, C. R. A. & Guo, Z. X. Strain and orientation modulated bandgaps and effective masses of phosphorene nanoribbons. *Nano Lett* **14**, 4607–4614 (2014).
60. Sung, J. *et al.* Long-Range Ballistic Propagation of Carriers in Methylammonium Lead Iodide Perovskite Thin Films. *Nat Phys* **16**, 171–176 (2019).
61. Ashoka, A. *et al.* Direct observation of ultrafast singlet exciton fission in three dimensions. *Nat Commun* **13**, 1–8 (2022).
62. Pogna, E. A. A. *et al.* Photo-induced bandgap renormalization governs the ultrafast response of single-layer MoS2. *ACS Nano* **10**, 1182–1188 (2016).
63. Taghizadeh Sisakht, E., Fazileh, F., Zare, M. H., Zarenia, M. & Peeters, F. M. Strain-induced topological phase transition in phosphorene and in phosphorene nanoribbons. *Phys Rev B* **94**, 085417 (2016).

64. Huang, S. *et al.* Layer-Dependent Pressure Effect on the Electronic Structure of 2D Black Phosphorus. *Phys Rev Lett* **127**, 186401 (2021).
65. Rudenko, A. N. & Katsnelson, M. I. Quasiparticle band structure and tight-binding model for single- and bilayer black phosphorus. *Phys Rev B* **89**, 201408 (2014).
66. Han, X., Morgan Stewart, H., Shevlin, S. A., Catlow, C. R. A. & Guo, Z. X. Strain and orientation modulated bandgaps and effective masses of phosphorene nanoribbons. *Nano Lett* **14**, 4607–4614 (2014).
67. Vlček, V., Rabani, E., Baer, R. & Neuhauser, D. Nonmonotonic band gap evolution in bent phosphorene nanosheets. *Phys Rev Mater* **3**, 064601 (2019).
68. Molas, M. R. *et al.* Photoluminescence as a probe of phosphorene properties. *NPJ 2D Mater Appl* **5**, 1–24 (2021).
69. Tian, R. *et al.* Observation of excitonic series in monolayer and few-layer black phosphorus. *Phys Rev B* **101**, 235407 (2020).
70. Surrente, A. *et al.* Excitons in atomically thin black phosphorus. *Phys Rev B* **93**, 121405 (2016).
71. Xu, R. *et al.* Exciton Brightening in Monolayer Phosphorene via Dimensionality Modification. *Adv Mat* **28**, 3493–3498 (2016).
72. Pei, J. *et al.* Producing air-stable monolayers of phosphorene and their defect engineering. *Nat Commun* **7**, 1–8 (2016).
73. Higashitarumizu, N. *et al.* Anomalous thickness dependence of photoluminescence quantum yield in black phosphorous. *Nat Nanotechnol* **18**, 507–513 (2023).
74. Nourbakhsh, Z. & Asgari, R. Excitons and optical spectra of phosphorene nanoribbons. *Phys Rev B* **94**, 1–9 (2016).
75. Wang, J. *et al.* Mid-infrared polarized emission from black phosphorus light-emitting diodes. *Nano Lett* **20**, 3651–3655 (2020).
76. Liess, M., Jeglinski, S., Lane, P. A. & Vardeny, Z. V. A three essential states model for electroabsorption in nonluminescent pi-conjugated polymers. *Synth Met* **84**, 891–892 (1997).
77. Ziffer, M. E., Mohammed, J. C. & Ginger, D. S. Electroabsorption Spectroscopy Measurements of the Exciton Binding Energy, Electron-Hole Reduced Effective Mass, and Band Gap in the Perovskite CH3NH3PbI3. *ACS Photonics* **3**, 1060–1068 (2016).
78. Ziffer, M. E., Mohammed, J. C. & Ginger, D. S. Electroabsorption Spectroscopy Measurements of the Exciton Binding Energy, Electron-Hole Reduced Effective Mass, and Band Gap in the Perovskite CH3NH3PbI3. *ACS Photonics* **3**, 1060–1068 (2016).
